# Can large language models reason about medical questions?

**DOI:** 10.1016/j.patter.2024.100943

**Published:** 2024-03-01

**Authors:** Valentin Liévin, Christoffer Egeberg Hother, Andreas Geert Motzfeldt, Ole Winther

**Affiliations:** 1 Section for Cognitive Systems, Technical University of Denmark, Anker Engelunds Vej 101, 2800 Kongens Lyngby, Denmark; 2 FindZebra, Rådvadsvej 36, 2400 Copenhagen, Denmark; 3 Department of Clinical Immunology, Copenhagen University Hospital, Rigshospitalet, Inge Lehmanns Vej 107, 2100 Copenhagen, Denmark; 4 Center for Genomic Medicine, Copenhagen University Hospital, Rigshospitalet, Ørestads Boulevard 5, 2300 Copenhagen, Denmark; 5 Bioinformatics Centre, Department of Biology, University of Copenhagen, Ole Maaløes Vej 5, 2200 Copenhagen, Denmark

**Keywords:** large language models, question answering, medical, GPT-3.5, Llama 2, open source, MedQA, prompt engineering, uncertainty quantification, machine learning

## Abstract

Although large language models often produce impressive outputs, it remains unclear how they perform in real-world scenarios requiring strong reasoning skills and expert domain knowledge. We set out to investigate whether closed- and open-source models (GPT-3.5, Llama 2, etc.) can be applied to answer and reason about difficult real-world-based questions. We focus on three popular medical benchmarks (MedQA-US Medical Licensing Examination [USMLE], MedMCQA, and PubMedQA) and multiple prompting scenarios: chain of thought (CoT; think step by step), few shot, and retrieval augmentation. Based on an expert annotation of the generated CoTs, we found that InstructGPT can often read, reason, and recall expert knowledge. Last, by leveraging advances in prompt engineering (few-shot and ensemble methods), we demonstrated that GPT-3.5 not only yields calibrated predictive distributions but also reaches the passing score on three datasets: MedQA-USMLE (60.2%), MedMCQA (62.7%), and PubMedQA (78.2%). Open-source models are closing the gap: Llama 2 70B also passed the MedQA-USMLE with 62.5% accuracy.

## Highlights

• InstructGPT can mobilize expert medical knowledge and reasoning skills
• With adequate prompting, both closed- and open-source models pass the USMLE (MedQA)
• LLMs can quantify uncertainty when applied to medical exam multiple-choice questions
• LLMs are affected by the order of the answer options (positional bias)

## The bigger picture

Foundation models have changed the way machine learning is practiced. Foundation models applied to text, so-called large language models (LLMs), have proven to be a disruptive technology. They might radically change the way we interact with computers.

In early 2022, it was clear that generalist LLMs can outperform domain-specific approaches in many domains. Benchmarks that reflect real-world scenarios were still needed, and today, it remains unclear how to best use and evaluate these models. This paper probes the capabilities of LLMs using medical exam questions (e.g., USMLE). Based on detailed step-by-step answers generated by LLMs, we show that LLMs can often answer challenging medical questions by mobilizing expert knowledge and advanced reasoning capabilities. Our results support that future LLMs might be applicable to critical real-world applications such as supporting healthcare professionals.

## Graphical abstract

**Figure:**
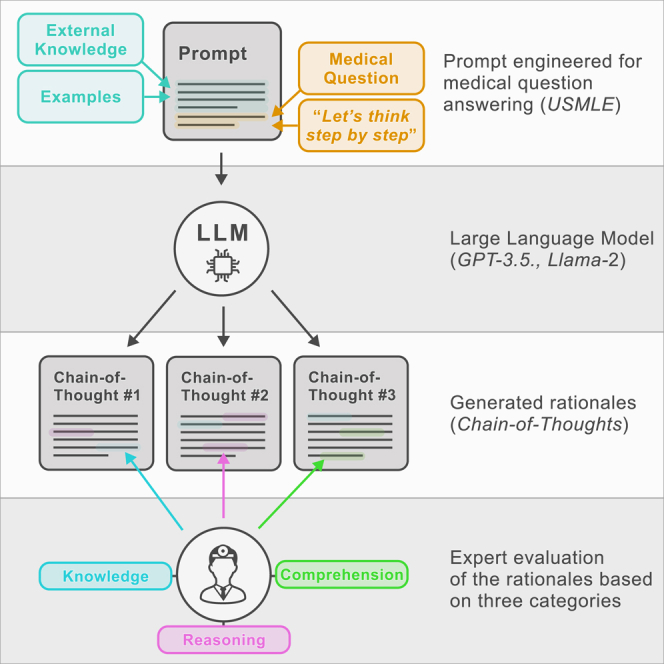

## Teaser

Large language models (LLMs) are powerful but their limits are unknown. This paper probes the capabilities of LLMs using medical exam questions (e.g., USMLE). Based on detailed answers generated by LLMs, we show that LLMs can often answer challenging medical questions by mobilizing expert knowledge and advanced reasoning capabilities. Both closed- and open-source LLMs can pass the USMLE (>60% accuracy). Our results support that future LLMs might be applicable to critical real-world applications such as supporting healthcare professionals.

## Introduction

Self-supervised pre-training promises to turn vast quantity of raw data (e.g., text, images, audio) into general-purpose models. Language representations have transformed the field of natural language processing from simple word vectors to deep contextualized representations,^1^^,^^2^^,^^3^^,^^4^^,^^5^^,^^6^ and language models are now ubiquitous in natural language processing. Notably, this ubiquity is thanks to the Transformer architecture and its compatibility with massively parallel computation hardware.^4^

### Large language models (LLMs)

In recent years, tremendous resources have been allocated to scale Transformer-based language models to using hundreds of billions of parameters and to training on gigabytes of text.^7^^,^^8^^,^^9^^,^^10^^,^^11^^,^^12^^,^^13^^,^^14^^,^^15^^,^^16^ This has so far translated to sustained gains^17^ and enabled new ways to interact with language models. This progress made many of the past benchmarks obsolete and sparked a general interest for designing difficult enough benchmarks (e.g., BIG-bench).^18^ Pre-train, prompt, and predict^19^ is an emerging paradigm for applying LLMs to new problems without fine-tuning the weights on the task. Prompt-based learning consists of augmenting the problem with instructions such that the model’s completion of the prompt will correspond to a solution. This allows for LLMs to learn from a few examples (coined shots), which are simply incorporated into the prompts.^7^

### Chain-of-thought (CoT) prompting

**Figure 1:**
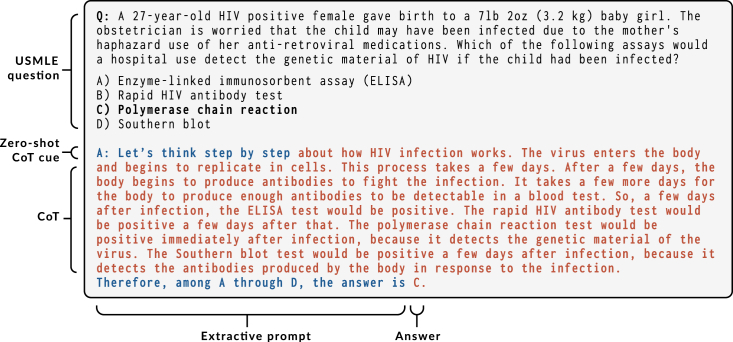
Answering a USMLE (US Medical Licensing Examination) question using zero-shot CoT prompting “Let’s think step by step” and InstructGPT Selected example.

Initially, scaling language models up appeared to benefit more knowledge-intensive tasks than the reasoning-heavy ones.^8^ Nevertheless, it was demonstrated that LLMs could be applied to system 2 problems by prompting the model to generate step-by-step solutions, coined CoT.^20^ CoT prompting led to substantial improvements on many reasoning-intensive tasks,^20^^,^^21^^,^^22^^,^^23^ allowing us to bridge the gap with human-level performances for most of the hard BIG-bench tasks.^24^ As an alternative to writing reference step-by-step solutions, zero-shot CoT allows for generating CoTs using a single and domain-agnostic cue: “Let’s think step by step”^25^ (see example generated by InstructGPT^26^ in Figure 1). The CoTs that result from that prompt not only appear to expose valid reasoning but also translate into superior zero-shot performances (see example in Figure 1).

### LLMs and medical applications

Applying LLMs to real-life scenarios will require implementing additional safeguards. Language models may amplify the social biases present in the training data, may hallucinate incorrect facts, and may lack robustness,^27^ for instance to adversarial attacks.^28^ Therefore, deploying LLMs into sensitive areas such as healthcare must be operated with great care.^29^^,^^30^ Nonetheless, LLMs are powerful tools and therefore have the potential to transform the field of machine intelligence. At the dawn of this research work, although LLMs had been tested on large benchmarks (MMLU,^31^ BIG-bench^18^), studies applied to the medical domain were still needed. Specialized datasets such as the MedQA-US Medical Licensing Examination (USMLE) enable assessing the capabilities of LLMs in realistic clinical scenarios requiring specialized medical knowledge, advanced reasoning capabilities, and human-level reading comprehension skills.^32^

### Related work

This article—written in three stages (v.1: July 2022, v.2: December 2022, and v.3: September 2023)—evolved along with the remaining of the field. December 2022 was a turning point in machine learning history; new records were achieved on medical benchmarks by the domain-specific Med-PaLM,^33^^,^^34^ ChatGPT, and GPT-4.^35^ ChatGPT sparked the interest of the public and the research community, which hastened to benchmark it against USMLE questions,^36^^,^^37^ turning to self-curated data instead of the peer-reviewed MedQA benchmark. Involving human experts to evaluate the generated explanations on USMLE questions has also been explored in concurrent work.^33^^,^^37^ Throughout the development of this research, significant progress happened in the open-source world (Llama 2^38^), and recently, there has been an investigation on both generalist and fine-tuned open-source LLMs applied to medical benchmarks.^39^ CoT prompting and ensemble methods are now commonplace in the literature, whereas retrieval augmentation (grounding) remains less common.^33^^,^^34^^,^^35^^,^^39^^,^^40^^,^^41^

### Contributions

This paper investigates the performances, interpretability, and limitations of CoT prompting for medical question answering. We utilized the GPT-3.5 series (InstructGPT and Codex). This research was conducted in three rounds; first, using InstructGPT, we investigated variations of zero-shot CoT prompting for medical reasoning (domain-specific CoT cues, retrieval augmentation), looking both at the answering performances and the limitations based on an expert evaluation. In the second round, thanks to the Codex beta program, we investigated how scaling inference-time compute could be applied to challenge both the human baseline and to quantify uncertainty. Last, we benchmarked a range of open-source models. Our contributions are as follows.

• We assess how GPT-3.5 performs on multiple-choice medical board exam question datasets (MedQA-USMLE and MedMCQA) and a medical reading comprehension dataset (PubMedQA) using prompt engineering. We explore zero-/few-shot, direct/CoT, domain-specific CoT cues and retrieval augmentation.
• We propose an evaluation protocol for evaluating generated CoTs (three main categories: reasoning, knowledge, and reading comprehension). A medical expert annotated a subset of CoTs generated by zero-shot InstructGPT and supports that InstructGPT, in many cases, can reason and exploit memorized expert knowledge.
• We demonstrate that scaling inference-time compute enables Codex 5-shot CoT to be well calibrated and to reach passing scores on the three medical datasets.
• We benchmark open-source LLMs on the MedQA-USMLE and MedMCQA.

### Development

This article has evolved over three distinct versions, each exploring different facets of LLMs:

• v.1, July 2022: investigated InstructGPT (expert evaluation and benchmarking prompting strategies).
• v.2, December 2022: scaled experiments and passed the MedQA-USMLE using Codex.
• v.3, September 2023: evaluated open-source models Llama 2, Vicuna, Guanaco, Falcon, etc.

## Method

**Figure 2:**
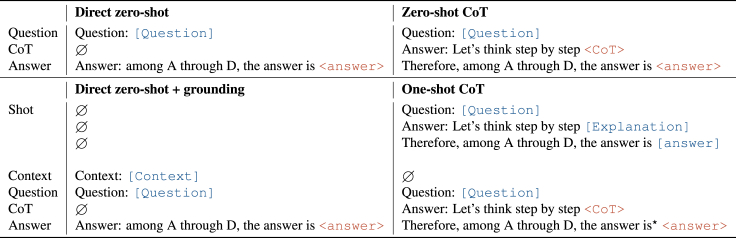
Prompt templates In the table, we use typewriter style and brackets to represent [provided data] such as the question, additional context, or the answer and <completions> generated by GPT-3. The symbol ∅ represents an empty string.

This paper explores variations of prompt engineering for medical question answering. The prompt templates are summarized in Figure 2.

### Zero shot

We studied two classes of prompts: the direct prompt and zero-shot CoT. The direct prompt triggers the model to generate the answer using a single completion step (i.e., “The answer is”), whereas when applying the zero-shot CoT framework, we use a two-step prompting scheme: first, an initial reasoning prompt with a CoT cue (e.g., “Let’s think step by step”), the completion of which is the CoT, and second, an extractive prompt, the completion of which is the answer (e.g., “Therefore the answer is”). In the zero-shot CoT setting, this corresponds to the setup described in Kojima et al.,^25^ and the direct setting corresponds to Brown et al.^7^

### Few-shot

We experimented with inserting examplars (or shots) of question-answer pairs and question-explanation-answers triplets in the prompts. We built each shot using the zero-shot template, replacing the output with the reference explanations and answers. In the few-shot CoT setting, our setup matches the one from Wei et al.^20^

### Answer likelihood

**Figure 3:**
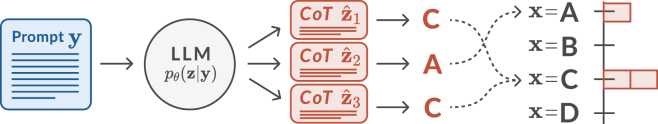
Generative process and answer likelihood (ensemble model, i.e., self-consistency)

We denote x the answer string, y a prompt, and z a completion generated from an LLM denoted $p_{\theta }$. In the zero-shot setting, sampling $\hat{z}∼p_{\theta }\left(z|y\right)$ is a two-step process (first generate the CoT, then extract the answer) pictured in Table 2. Using a sampling temperature τ, *k* completions ${\hat{z}}_{1},\ldots ,{\hat{z}}_{k}$ can be sampled from the generative LLMs. We aggregate the completions and estimate the marginal answer likelihood as (Figure 3)^42^:

(Equation 1) $$p_{\theta }\left(x|y\right)\approx \frac{1}{k}\sum_{i=1}^{k}1\left[x\in {\hat{z}}_{i}\right],\;\;{\hat{z}}_{1},\ldots ,{\hat{z}}_{k}∼p_{\theta }\left(z|y\right)$$

where $1\left[x\in {\hat{z}}_{i}\right]$ takes value one when the answer x can be matched in the completion $\hat{z}$ and otherwise takes zero. Sampling multiple completions may allow exploring multiple hypotheses. Combining multiple sampled CoTs (also known as self-consistency) has also been explored in past work, and showed improvements over single-sample CoT methods.^42^^,^^43^

**Table 1:** Answering accuracy of leading models against human performance on USMLE (test), MedMCQA (validation), and PubMedQA (test) datasets

Model	Date	USMLE	MedMCQA	PubMedQA
Codex 5-shot CoT^a^	2022	60.2	59.7	78.2
Llama 2 5-shot CoT^a^	2023	62.5	53.6	–
Fine-tuned SOTA	2022	50.3	52.9	78.2
GPT-4	2023	86.1	**73.7**	**81.2**
MedPalm v.2	2023	**86.5**	72.3	77.4
Human (passing score)	–	60.0	50.0	–
Human (expert score)	–	87.0	90.0	78.0

Find an overview of our results in supplemental information section A.

a Our best methods.

**Table 2:** Summary of the medical question answering datasets

	MedQA-USMLE^32^	MedMCQA^44^	PubMedQA^45^
Answer options	A/B/C/D	A/B/C/D	yes/no/maybe
Questions (train/valid./test)	10,200/1,300/1,300	182,800/4,200/6,100	450/50/500
Words/question	116.6	12.7	253.3
Source (questions)	national medical board examination (US)	AIIMS and NEET PG entrance exams	expert-annotated PubMed abstracts
Words/explanation	41.6	66.2	43.2
Source (explanations)	5 human-written CoTs (sourced from MMLU^46^)	detailed explanations (original dataset)	long answer (original dataset)

valid., validation.

### Retrieval augmentation

LLMs memorize part of the knowledge embedded into the training data; nonetheless, models might fail to reuse this knowledge effectively during prediction. Conditioning the predictions on a knowledge base is an alternative research direction for improving language models.^47^^,^^48^^,^^49^

We investigated whether grounding the model with additional context could improve the answering accuracy. We experimented with a simple BM25 retriever and used Wikipedia as a knowledge base. Read more details in supplemental information section G.

## Experiments

This section is separated into three parts: (1) introducing the datasets and the GPT-3.5 models, (2) investigating zero-shot medical reasoning with InstructGPT, and (3) scaling inference-time compute with Codex (using longer few-shot prompts and sampling many completions per question).

### Resources availability

#### Lead contact

Further information and requests for code and data should be directed to and will be fulfilled by the lead contact, Valentin Liévin (valentin.lievin@gmail.com).

#### Materials availability

This study did not generate new unique materials or reagents.

#### Data and code availability

Our source code is available on Github (https://github.com/vlievin/medical-reasoning).^50^ A collection of generated CoTs, reusable for downstream tasks, are accessible through ToughtSource.^51^ All our benchmark results are summarized in supplemental information section A and Table S2.

### Datasets and models

#### Datasets

This study is centered around three medical multiple-choice question answering datasets: USMLE, which includes difficult real-world medical questions targeting medical professionals^32^; the MedMCQA, which gathers questions from medical school entrance exams^44^; and the PubMedQA, which includes reading comprehension questions about PubMed abstracts.^45^ The three datasets are summarized in Table 2. For each dataset, we gathered questions with explanations (long answer), which we used as reference CoTs in few-shot learning scenarios. We present the three datasets in further details in supplemental information section C. Furthermore, we compare the MedQA-USMLE with the MMLU-USMLE dataset in supplemental information section D; we found the MedQA questions to be more challenging than the MMLU ones.^31^

#### Models

We study a collection of closed- and open-source models. The 175-billion parameter GPT-3.5 series: the human-aligned GPT-3 (InstructGPT, text-davinci-002^26^) and the code-fine-tuned GPT-3 (Codex, code-davinci-002).^52^ A collection of open-source models ranging from 7 to 70 billion parameters: Llama 2,^38^ Vicuna,^53^ Guanaco,^54^ Falcon,^55^ MPT,^56^ and GPT-NeoX.^57^ We used greedy decoding (temperature τ=0) with k=1 sample unless specified otherwise (e.g., ensemble methods).

In supplemental information section E, we report the test USMLE accuracy for four GPT-3 versions: a small GPT-3, the largest GPT-3 trained without human alignment, InstructGPT, and Codex. The smaller model text-curie-002 delivered close to random performances, with a maximum accuracy of 27.9%. The non-aligned largest GPT-3 text-davinci-001 scored 40.2%, whereas the largest code pre-trained Codex scored 52.9%, and the code-pre-trained and human-aligned InstructGPT scored 47.1%.

### Investigating zero-shot reasoning with InstructGPT

In this section, we investigate whether the good generative capabilities of LLMs can be applied to answer medical questions in a zero-shot setting. We investigate variations of the zero-shot CoT framework: using domain-specific CoT cues and augmenting the prompt with Wikipedia passages.

In addition to the original zero-shot CoT cue “Let’s think step by step,” we tested 29 other domain-specific variations such as “Let’s think step by step like a medical expert.” The study is available in supplemental information section B. We selected five CoT cues displayed in Table 3. In supplemental information section I, we display CoT samples for more exotic cues such as “Let’s follow a Bayesian step by step approach,” “Let’s work by elimination,” and “Let’s reflect on each answer option.”

**Table 3:** Selected domain-specific CoT cues

#1 – *Let’s think step by step*
#2 – *Let’s think step by step like a medical expert*
#3 – *Let’s use step by step inductive reasoning, given the medical nature of the question*
#4 – *Let’s differentiate using step by step reasoning like a medical expert*
#5 – *Let’s derive the differential diagnosis*

### Zero-shot benchmark

In Table 4, we report the performances of InstructGPT for the direct prompt and the aggregated performances for the five domain-specific CoT cues (Table 3). We explored augmenting the prompts with retrieved Wikipedia passages (grounding) and report the performances of an ensemble model with majority voting.^42^

**Table 4:** Zero-shot answering accuracy of InstructGPT (text-davinci-002) on the MedQA-USMLE (test), MedMCQA (valid), and PubMedQA (test) datasets

Model	Grounding	Prompt	USMLE	MedMCQA	PubMedQA
InstructGPT	∅	direct	46.0	44.0	**73.2**
InstructGPT	∅	CoT #1–#5	46.1 ± 0.7	40.4 ± 2.2	59.9 ± 3.5
InstructGPT	BM25	direct	47.3	46.7	–
InstructGPT	BM25	CoT #1–#5	46.4 ± 0.7	42.5 ± 1.7	–
InstructGPT	∅	ensemble (n = 6)	50.0	42.4	70.4
InstructGPT	BM25	ensemble (n = 6)	49.3	**48.8**	–
InstructGPT	∅ + BM25	ensemble (n = 12)	**53.1**	47.6	–
Fine-tuned BERT	BM25, DPR, ∅	–	44.6	43.0	72.2
Human (passing score)	–	–	60.0	50.0	–
Human (expert score)	–	–	87.0	90.0	78.0

We report the best fine-tuned BERT-based methods. We tested 5 domain-specific CoT cues (#1–#5) and report the mean performances with standard deviations. Fine-tuned BERT, BioLinkBERT^58^; DPR, dense passage retrieval.^59^ When multiple results are aggregated, we report the mean and standard deviation (±).

#### Zero-shot direct

InstructGPT outperformed the domain-specific and fine-tuned BERT baselines on the three datasets. Without BM25 grounding, InstructGPT scored +1.4% on the USMLE questions, +1.0% on the MedMCQA exam questions, and +1.1% on PubMedQA over the best BERT methods.

#### Zero-shot CoT

Without BM25 grounding, the direct prompt remained, on average, a better alternative to the CoT prompts. Performances were lower for each of the considered CoT cues, except in the case of the USMLE dataset, for which half of the CoT prompts resulted in small improvements over the direct prompt (+1.1% using CoT prompt #1 vs. using the direct prompt). Nonetheless, the domain-specific CoT prompts #2–#5 did not significantly outperform the original CoT prompt #1.

#### Knowledge grounding

In an attempt to exploit the good reading comprehension skills of InstructGPT, we explored conditioning the completions on Wikipedia passages. When using the direct prompt, we recorded gains on the USMLE (+1.3%) and on the MedMCQA (+2.7%) datasets, suggesting that retrieval augmentation might be beneficial.

#### Ensemble

Combining the predictions of multiple prompts outperformed the single-prompt predictions, except in the case of the PubMedQA dataset, for which the direct prompt performed exceptionally well. The best performances on the USMLE and MedMCQA datasets were obtained by combining retrieval-augmented prompts and setting a maximum of 53.1% accuracy on the USMLE dataset and 48.8% validation accuracy on the MedMCQA dataset.

### Expert evaluation of the generated CoTs

#### Protocol

InstructGPT delivered strong performances using zero-shot CoT prompting. In this section, we investigate whether the CoTs are sound and seek to understand better how the model fails and succeeds. We considered three general skills that we expect are required to be mastered to answer medical questions: (1) the ability to perform non-trivial reasoning steps, (2) the ability to recall knowledge that is not provided in the context, and (3) the ability to comprehend the question and the context. Based on the three skills, we defined three success patterns (A, B, C) and three failure patterns (D, E, F).

A subset of 50 CoTs generated based on USMLE questions were annotated by a medical expert (C.E.H.) using the six categories. For each category and each CoT, we reported a match if the pattern could be observed at least once. This means that a CoT can be labeled with both a correct and an incorrect pattern for the same skill. We showcase thirty annotated CoTs (three in Figure 9 and 27 in supplemental information section I).

#### Analysis

We report the frequencies of occurrence for the six patterns in Table 5. We found that most of the questions answered incorrectly triggered generating CoTs that contained reasoning errors (pattern D, 86%) and that exhibited a lack of knowledge (pattern E, 74%). Misunderstanding of the questions or the context was less frequently observed (pattern F, 50%). We observed that CoTs leading to questions answered correctly could still show failure patterns but we also observed that the CoTs leading to incorrect answers were not entirely incorrect, as 59% contained at least one correct reasoning step and 65% showed proper recall of knowledge. Furthermore, inspecting the CoTs leading to incorrect answers more closely, we found that 47% of those were inconclusive: the model could not narrow down the prediction to a single answer.

**Table 5:** Frequency of observed patterns (A, B, C, D, E, F) identified among 50 CoTs generated by InstructGPT with temperature τ = 0

	Pattern	Correct, % (16)	Incorrect, % (34)	Total, % (50)
**A**	correct reasoning step	94 (15)	59 (20)	**70** (35)
**B**	correct recall of knowledge	87 (14)	65 (22)	**72** (36)
**C**	correct reading comprehension	100 (16)	85 (29)	**90** (45)
**D**	incorrect reasoning step	12 (2)	86 (29)	**62** (31)
**E**	incorrect or insufficient knowledge	25 (4)	74 (25)	**58** (29)
**F**	Incorrect reading comprehension	6 (1)	50 (17)	**36** (18)

The CoTs are generated based on USMLE questions and using the CoT prompts #1–#5 (Table 3). We report the frequencies of CoTs leading to correct and incorrect predictions along with the total.

#### Answering bias

**Figure 4:**
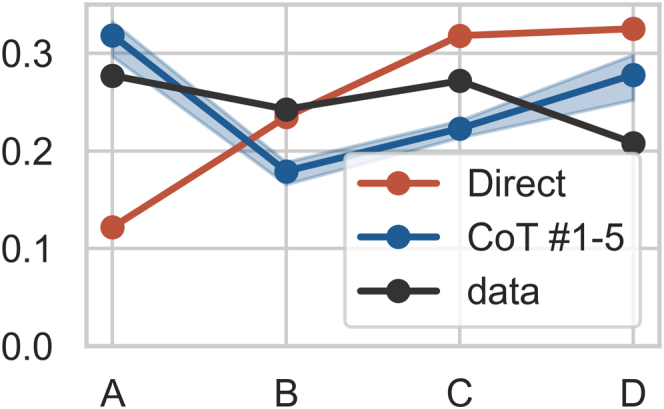
Frequencies of USMLE answers and InstructGPT (text-davinci-002) predictions for direct and CoT prompts (no grounding, zero-shot)

In Figure 4, we report the frequencies of the USMLE answers and the frequencies of predicted labels (zero-shot InstructGPT) for the direct and CoT prompts. Both prompting schemes led to biased predictive frequencies. Direct prompting led to overestimating labels C and D while underestimating label A. CoT prompting led to underestimating B and C while overestimating label D. We repeated the experiment using randomly permuted labels and observed similar patterns (see supplemental information section F).

#### Scaling inference-time compute with codex

In the second round of experiments, we investigated whether using more inference-time compute, thanks to the Codex beta program, could be utilized to obtain better performances and more interpretable outputs. Codex enables using longer prompts, so we used 5-shot prompts and experimented with sampling k=100 completions with temperature τ=0.5 for each question. We report question-answering performances and results on uncertainty quantification.

#### Codex 5-shot CoT: Sampling and combining multiple CoTs

**Figure 5:**
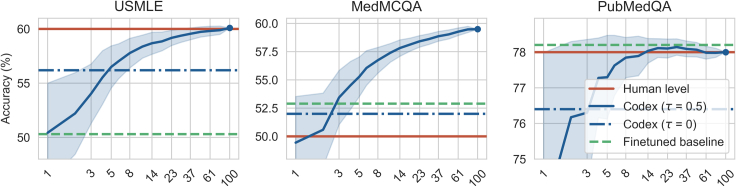
Sampling and combining multiple CoTs Answering accuracy of Codex 5-shot CoT (code-davinci-002) on the USMLE (test), the MedMCQA (validatuin), and the PubMedQA (test) datasets for 100 CoTs sampled with temperature τ∈{0,0.5}. We report the average accuracy for ensemble models evaluated using random subsets of $k^{\prime }=1\ldots 100$ CoTs. We report the mean and standard deviation. We display the performances of the best fine-tuned methods along with the lower human baselines.

In Figure 5, we report the performances of Codex 5-shot CoT given subsets of $k^{\prime }<k$ CoTs. We report the best fine-tuned models and the human baseline. Increasing the budget of samples yields better results.^42^ Using an ensemble of the *k* samples, Codex 5-shot CoT reaches the passing score on the three tasks (see Table 1): the USMLE dataset (60.2% ≥ 60%), the MedMCQA dataset (62.7% ≥ 50%), and the PubMedQA dataset (78.2% ≥ 78%). Additional results, including performances in zero-shot settings, are available in Table S2 (supplemental information section A). Although Codex performed exceptionally well with 5 shots, Codex yield feeble performances with zero-shot CoT; inspecting the generated CoTs revealed lesser-quality samples (supplemental information section I).

#### Uncertainty quantification

**Figure 6:**
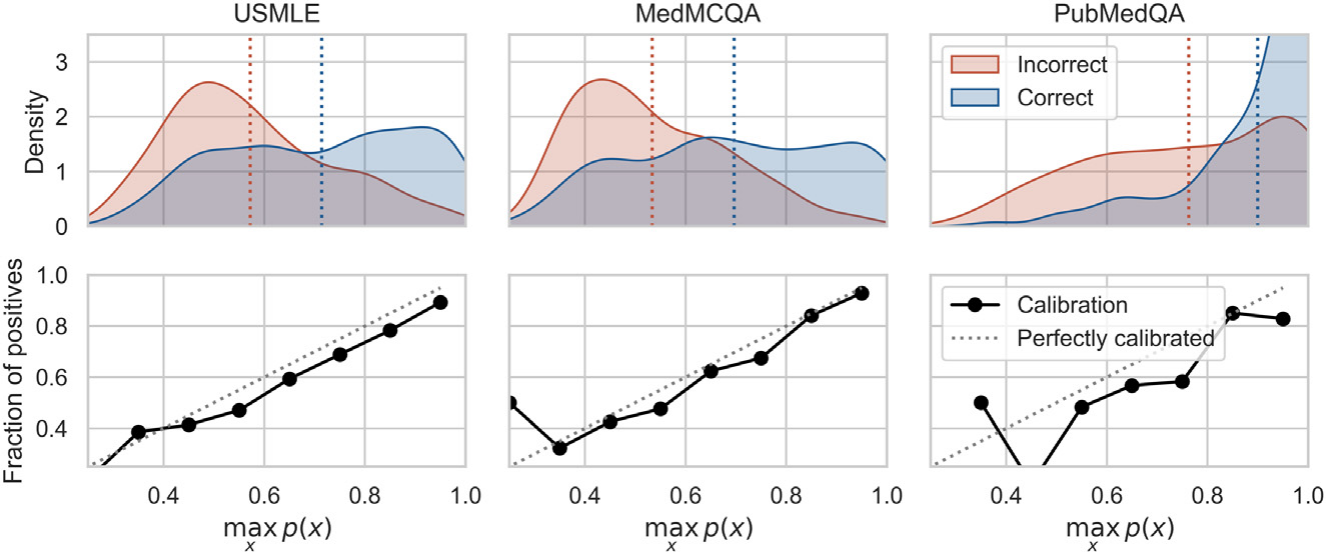
Uncertainty quantification First row: distribution of the probability assigned to the correct label for correct predictions and incorrect predictions (see Equation 1). Second row: calibration plot. The probabilities are obtained using Codex 5-shot CoT and an ensemble of k=100 predictions sampled with temperature τ=0.5.

We investigate the answering likelihood Equation 1 given by Codex 5-shot CoT with k=100 samples. In Figure 6, we report the maximum probability assigned by the model for correctly vs. incorrectly answered questions along with the calibration plots for the three datasets. Codex 5-shot CoT appears to be overall calibrated, although the calibration is worse for the PubMedQA dataset.

#### Benchmarking open-source models

**Figure 7:**
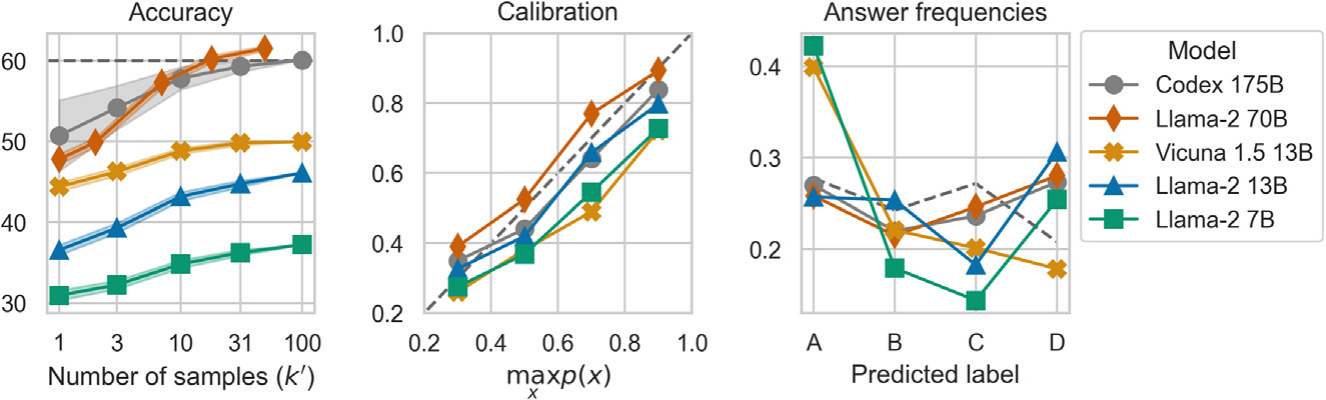
Comparing open-source LLMs against the closed-source Codex on the MedQA-USMLE benchmark (τ=0.9, up to k=100 samples) We report answering accuracy, model calibration, and answering bias.

In the rapidly evolving landscape of LLMs, a prevalent question is the performance gap between open-source and closed-source models. Our study focused on the capabilities of InstructGPT and Codex. Given a budget of 2.000 GPU hours (NVIDIA A100), we benchmarked a range of open-source LLMs, with parameter sizes ranging from 7 to 70 billion, against the 175-billion-parameter Codex. In Figure 7, we report the predictive performances, calibration plots, and biases for Llama 2, Vicuna 1.5, and Codex using up to k=100 CoT samples. We provided additional results in Figure 8 in supplemental information section H (zero- and 5-shot, MedQA-USMLE, and MedMCQA).

**Figure 8:**
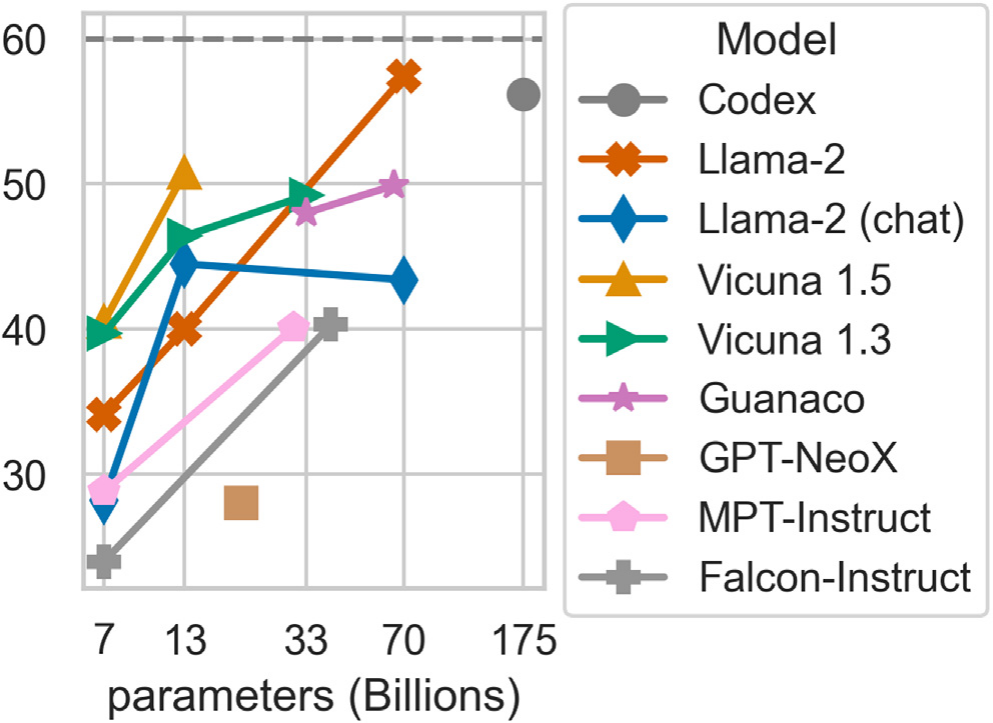
MedQA-USMLE accuracy vs. model size All experiments were performed using a 5-shot CoT prompting strategy and greedy decoding (τ=0). Llama 2 70B outperforms Codex 175B (proprietary).

**Figure 9:**
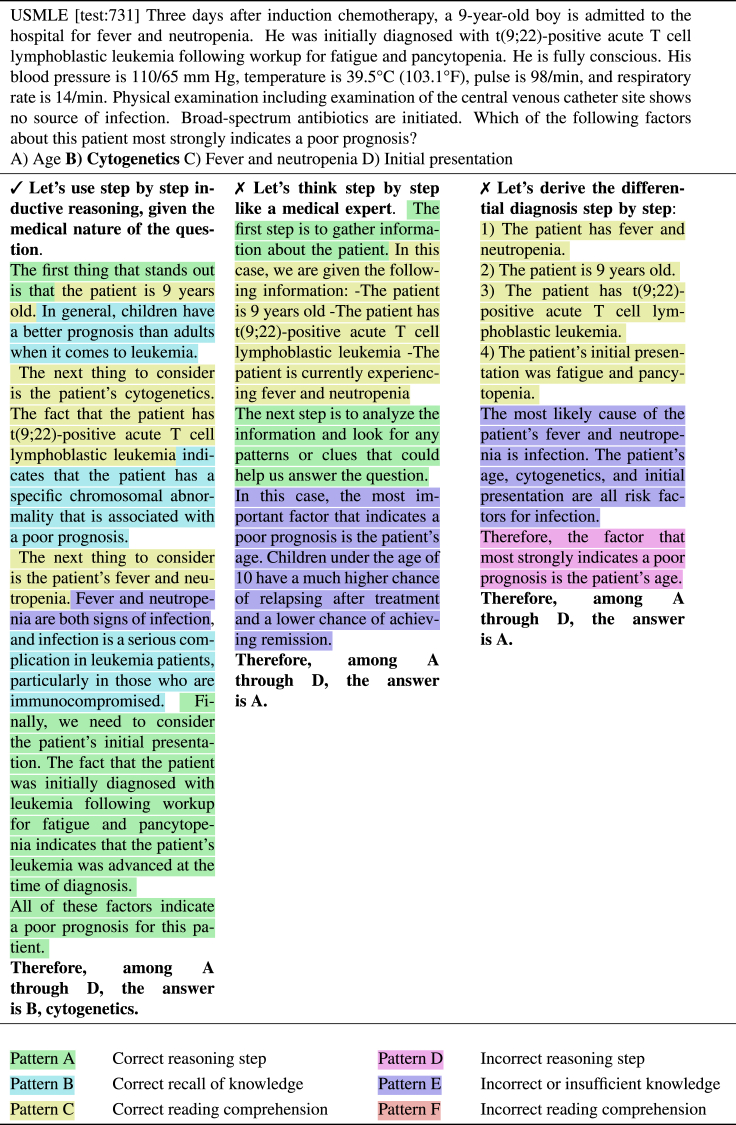
(Sample 1) Generated zero-shot CoT from InstructGPT text-davinci-002 for three CoT prompts on a sample for the MedQA-USMLE test set

## Discussion

### Zero-shot LLMs outperform fine-tuned BERT

Zero-shot InstructGPT and Codex outperformed fine-tuned BERT models on three challenging question-answering datasets (see zero-shot benchmark and supplemental information section A). In the case of the USMLE and the MedMCQA datasets, the retrieval-augmented BERT baselines were outperformed by several LLMs, regardless of augmenting the prompts with Wikipedia passages. This suggests that LLMs, without fine-tuning, can mobilize medical knowledge and problem-solving skills.

### Zero-shot CoT prompting often yields sound and interpretable step-by-step solutions

For both InstructGPT and Codex, single-sample CoT prompting was not found to be competitive with direct prompting (see zero-shot benchmark and supplemental information section A). Nevertheless, CoTs are human readable and therefore interpretable. Our expert evaluation (see expert evaluation of the generated CoTs) revealed that CoTs are often sound: even though InstructGPT still does make mistakes, it was often able to reason, recall medical knowledge, and comprehend the given problem. In the section investigating zero-shot reasoning with InstructGPT and supplemental information section B, we explored domain-specific CoTs cues such as “Let’s think step by step like a medical expert.” Although such prompts, taken separately, did not outperform the original zero-shot CoT prompt (see Table S2 in supplemental information section A), more specific prompts appeared to trigger alternative strategies such as working by elimination or manipulating equations (see supplemental information sections B and I). Investigating whether a task-specific prompt could help solve specific tasks will be left for future research. A collection of generated CoT samples are presented in supplemental information section I, and many more samples are available on our GitHub page.

### LLMs memorize some expert knowledge

The expert evaluation of the generated CoTs (see expert evaluation of the generated CoTs) and the good results obtained on the medical exam questions (see Table S2; supplemental information section A) suggest that GPT-3.5 memorizes domain knowledge. Nevertheless, despite the simplicity of the BM25 retriever and the small number of retrieved documents prepended in each prompt, grounding InstructGPT resulted in slight improvements (see Table 4). This suggests that InstructGPT is not omniscient, and so (1) using stronger retrievers such as commercial search engines or dense retrievers,^49^ (2) using a more complete knowledge base,^48^ or (3) leveraging inference-time compute by retrieving, reranking, and processing more passages^49^ might improve performances.

### Bias

In the section answering bias, we exposed the biases induced by the use of direct and CoT prompts. In the case of the direct prompt, answer D was most often selected, which might be due to its proximity to the generated answer. In the case of the CoT prompts, labels A and D were selected more often, which might be a result of often beginning CoTs with content related to option A. Based on an inspection of the CoTs, we speculate that GPT-3 defaults to this behavior when it cannot answer but still attempts to complete the prompt with a default answer (D or A). Shuffling the answer options might be one way to overcome this limitation; however, other forms of biases might still be present.

### Generating and combining many CoTs bridges the gap with human-level performances

CoTs can be combined and/or filtered using human or automated feedback.^42^^,^^60^ In the section scaling inference-time compute with Codex, we showed that sampling and combining up to k=100 completions using Codex or Llama 2 with 5-shot CoT prompts was sufficient to pass both the MedMCQA and the challenging USMLE, although a large gap remains between our models and the human experts.

### 5-Shot CoT-prompted LLMs are close to well calibrated

In the sections uncertainty quantification and benchmarking open-source models, we looked at the probability assigned to correct and incorrect predictions using the ensemble model from Equation 1. We found Codex and Llama 2 to be close to well calibrated, corroborating the results that “language models (mostly) know what they know.”^61^

### Scale, code pre-training, human-alignment, and few-shot learning

In supplemental information section E, we compared multiple GPT-3 models in the zero-shot setting. Best performances are obtained using Codex, outperforming the human-aligned InstructGPT, which is a fine-tuned version of Codex. Human alignment might impair performances; Codex (without alignment) was not as robust as InstructGPT (with alignment) in zero-shot CoT setting (see performances in Table S2 in supplemental information section A and see CoT samples in supplemental information section I). Nevertheless, 5-shot prompting allowed us to bypass the zero-shot limitations of Codex. We observed a similar pattern when comparing the versions of LLama-2 70b: the base version outperformed the chat version (supplemental information section H). Instruction-fine-tuned models might lose in-context learning abilities.

### Open-source models narrow the gap with proprietary counterparts

Open-source models, despite having fewer parameters, are approaching the performance of proprietary ones (Figures 7 and 8). For instance, Llama 2 outperforms Codex with just half the parameters.

Instruction-fine-tuned LLMs like Guanaco and Vicuna performed exceptionally well (Figure 8). Surprisingly, Vicuna 1.5 13B’s superior performance to both Llama 2 versions underscores the significance of high-quality datasets for instruction-based fine-tuning.^62^

### Conclusion

We applied zero-shot, few-shot direct, and CoT prompting to medical question answering with and without retrieval augmentation. Zero-shot InstructGPT significantly outperformed the fine-tuned BERT baselines. CoT prompting proved to be a powerful tool leading to better performances and more interpretable predictions. Our expert evaluation suggests that LLMs can mostly comprehend complex medical questions, can often recall expert-domain knowledge, and can often perform non-trivial reasoning steps.

Although InstructGPT and Codex still make mistakes, we found that scaling inference-time compute by sampling many CoTs per question could overcome part of these limitations. With 100 samples, Codex 5-shot CoT delivered unprecedented performances on the three datasets, bridging the gap with human-level performances and virtually passing the USMLE by 0.2% points. Our exploration into open-source LLMs indicated their competitive stance in medical benchmarks. Llama 2 outperformed Codex by 2 points on the USMLE in spite of a much smaller parameter footprint.

However, deploying LLMs in real-life clinical scenarios will require the development of more robust techniques. We exposed one form of bias (ordering of the answer options affects the predictions), but many more might affect predictions, including those hidden in the training data (e.g., gender, race, …). Nevertheless, a lack of knowledge might be more easily compensated; our experiment with BM25, albeit limited, suggests that augmenting the prompt with factual data improves performances.

Since the completion of v.2 of this work, both GPT-4 and MedPalm 2 have achieved performance on USMLE around 85%.^35^^,^^63^ This is not unexpected given the evolution the LLM field has witnessed recently. Although benchmark contamination in training sets for both proprietary and open-source LLMs is a valid concern, these results indicate that both open- and closed-source LLMs hold great potential for assisting human decision-making in medicine and beyond.

## Supplemental information

Document S1. Supplemental information, Figures S1–S14, and Tables S1–S5

Document S2. Article plus supplemental information

## Footnotes

Supplemental information can be found online at https://doi.org/10.1016/j.patter.2024.100943.
