# Supplementary material for: Can large language models reason about medical questions?
Source: Patterns (N Y). 2024 Mar 1;5(3):100943. doi: 10.1016/j.patter.2024.100943 (PMC10935498; doi:10.1016/j.patter.2024.100943)
Supplement: Document S1. Supplemental information, Figures S1–S14, and Tables S1–S5 [file mmc1.pdf]

Patterns, Volume *5*

Supplemental information

Can large language models reason about medical questions?

Valentin Liévin, Christoffer Egeberg Hother, Andreas Geert Motzfeldt, and Ole Winther

A Summary of the Results

In Table S2, we summarize the performances of InstructGPT and Codex on the MMLU-USMLE, MedQA-USMLE, MedMCQA and PubMedQA datasets in zero-shot, few-shot, with and without grounding. All our results on the validation set of the MedMCQA are estimated using 1k samples. The results of the MedMCQA test set require submitting an official submission. We used a sampling temperature of $\tau = 0$ for all experiments except when drawing $k > 0$ samples and using majority voting (MV). For the majority voting model, we used $k = 100$ samples and $\tau = 0.5$ for Codex, $\tau = 0.9$ for Vicuna.

B Domain-specific CoT cues

Table S1: Validation performances for 30 CoT cues on a subset of 100 validation USMLE questions.

	CoT cue	Accuracy	F1	CoT length
0	*Let's derive the differential diagnosis step by step*	**<u>48.0</u>**	48.0	170
1	*Let's use step by step inductive reasoning, given the medical nature of the question*	**<u>48.0</u>**	**<u>48.2</u>**	157
2	*Let's differentiate using step by step reasoning like a medical expert*	47.0	46.3	183
3	*Let's think step by step using deductive reasoning*	47.0	46.4	148
4	*Let's differentiate using step by step reasoning*	45.0	45.0	166
5	*Let's think step by step to arrive at one of the options*	45.0	45.0	158
6	*Let's break the problem into multiple steps*	45.0	44.2	165
7	*Let's use step by step deductive reasoning, given the medical nature of the question*	44.0	44.0	174
8	*Let's think step by step like a doctor*	43.0	43.3	162
9	*Let's think step by step like a medical expert*	43.0	42.8	171
10	*Let's summarize the facts step by step*	42.0	42.1	183
11	*Let's think step by step using inductive reasoning*	42.0	42.6	143
12	*Let's think step by step using deductive reasoning like a medical expert*	42.0	42.3	173
13	*Let's be concise and think step by step*	42.0	42.4	130
14	*Let's differentiate using step by step deductive reasoning like a medical expert*	42.0	41.9	173
15	*Let's argue step by step*	42.0	42.2	149
16	*Let's think step by step like a clinician*	41.0	41.3	164
17	*Let's think step by step*	40.0	40.4	129
18	*Let's reflect on each answer option step by step*	40.0	37.2	194
19	*Let's reason and differentiate options step by step like a medical expert*	40.0	38.1	180
20	*Let's differentiate using step by step inductive reasoning like a medical expert*	40.0	39.5	161
21	$\varnothing$ *(Direct)*	39.0	38.4	0
22	*Let's think step by step given every option equal consideration*	39.0	39.2	177
23	*Let's think step by step like a scientist*	39.0	39.2	166
24	*Let's use step by step inductive reasoning*	37.0	36.1	165
25	*Let's work by elimination step by step*	36.0	35.2	154
26	*Let's use step by step deductive reasoning*	34.0	33.9	165
27	*Let's follow a Bayesian step by step approach*	33.0	31.4	193
28	*Let's reflect on each option from the least likely to the most likely*	31.0	27.9	166
29	*Let's use step by step Bayesian reasoning, given the medical nature of the question*	31.0	30.7	216

We composed an initial set of 30 zero-shot CoT prompt variations. In Table S1, we report the accuracy for each of the 30 prompts based on a subset of 100 USMLE validation questions. Given an estimated accuracy uncertainty of 5% (see the paragraph "uncertainty estimation" below), we concluded that the first half of the results are all reasonable candidates for the study.

Selected prompts For the remaining of this paper, we selected 5 prompts: the original "*Let's think step by step*", the medical variation "*Let's think step by step like a medical expert*" and the top-three CoT cues reported in Table S1.

Prompt diversity and agreement In Figure S1, we report the agreement rate for all 30 prompts on the 100 validation questions. Whereas most of the prompts followed a rather consistent pattern, with an agreement rate superior to 50%, a minority of the prompts seemed to agree less with the majority of the prompts, such as “Let’s reflect on each answer option step by step”, “Let’s follow a Bayesian step by step approach” or “Let’s work by elimination”. In Figure S4, we showcase four chain-of-thoughts selected to highlight the diversity of the completions and the ability of InstructGPT to adopt diverse problem-solving strategies. Yet, strategies are not always executed correctly: in Figure S4, example 2, GPT-3 ultimately finds the correct answer (Missense mutation) but identified the wrong diagnostic (the 6-year-old boy suffers from sickle cell disease).

Uncertainty estimation We model the outcome of answering a question using a $\mathrm{Bernoulli}$ model with parameter θ where 1 corresponds to the correct predicted answer, and 0 corresponds to predicting the wrong answer. The accuracy of the model corresponds to the mean outcome of the $\mathrm{Bernoulli}$ model ($\mathbb{E}\left[\mathrm{Bernoulli}(\theta)\right] = \theta$) that we approximate as $\theta = 0.5$. Given N=100 data points, the uncertainty of the accuracy estimate is about 5%, as given by the standard deviation of the mean estimator:

$$\sqrt{\mathrm{Var}_N\left[\mathrm{Bernoulli}(\theta)\right]} = \sqrt{\frac{\theta(1-\theta)}{N}} = 0.5^2/100 = 0.05\ (5\%)\ .$$

Figure S1: Rate of agreement for the 30 evaluated CoT prompts evaluated in Table S1.

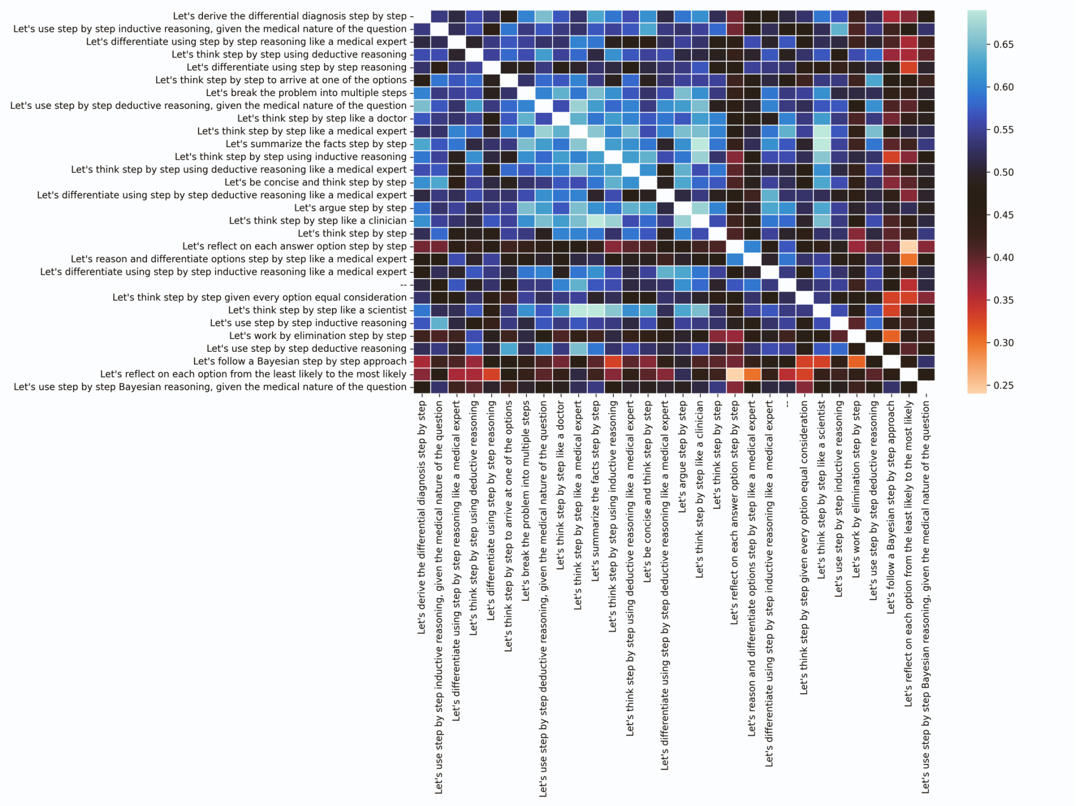

Table S2: Question answering accuracy on the MedQA-USMLE, MedMCQA (valid. 1k samples/test), and PubMedQA datasets. The CoTs cues #1–5 are defined in Table 3 (e.g., #2 = *Let's think step by step like a medical expert*). Results marked with ⋆ represent the pinnacles of our observations.

Model	Shots	Grounding	Prompt	MMLU⋆	USMLE	MedMCQA	PubMedQA
InstructGPT (175B)	0	✗	Direct	–/ –	46.0	44.0/ –	73.2
InstructGPT (175B)	0	✗	CoT #1	–/ –	47.1	40.8/ –	60.0
InstructGPT (175B)	0	✗	CoT #2	–/ –	46.8	43.3/ –	59.8
InstructGPT (175B)	0	✗	CoT #3	–/ –	46.0	38.8/ –	66.2
InstructGPT (175B)	0	✗	CoT #4	–/ –	45.6	37.1/ –	58.0
InstructGPT (175B)	0	✗	CoT #5	–/ –	45.1	42.1/ –	55.6
InstructGPT (175B)	0	✓	Direct	–/ –	47.3	46.7/ 49.0	–
InstructGPT (175B)	0	✓	CoT #1	–/ –	45.9	42.2/ 46.0	–
InstructGPT (175B)	0	✓	CoT #2	–/ –	47.0	45.8/ 46.0	–
InstructGPT (175B)	0	✓	CoT #3	–/ –	45.6	41.6/ 43.3	–
InstructGPT (175B)	0	✓	CoT #4	–/ –	45.9	41.3/ 45.0	–
InstructGPT (175B)	0	✓	CoT #5	–/ –	47.4	41.8/ 46.5	–
InstructGPT (175B)	0	✗	Ensemble (n=6)	–/ –	50.0	42.4/ –	70.4
InstructGPT (175B)	0	✓	Ensemble (n=6)	–/ –	49.3	48.8/ 49.5	–
InstructGPT (175B)	0	✗ + ✓	Ensemble (n=12)	–/ –	53.1	47.6/ –	–
Codex (external results)[1]	0	✗	Direct	–/ –	–	–/ 54.4	–
Codex (175B)	0	✗	Direct	74.2/ 70.6	52.5	50.9/ 51.1	73.2
Codex (175B)	0	✗	CoT #1	64.5/ 60.7	52.9	46.8/ –	53.4
Codex (175B)	0	✓	Direct	64.5/ 68.7	52.5	50.8/ 52.7	–
Codex (175B)	0	✓	CoT #1	77.4/ 55.1	47.2	43.9/ –	–
Codex (175B)	5	✗	Direct	77.2/ 70.1	56.6	56.6/ 56.9	73.0
Codex (175B)	5	✗	CoT #1	⋆80.6/ 68.4	56.2	52.0/ –	76.4
Codex (175B)	5	✗	CoT #1 + MV(k=20)▽	74.2/ ⋆**76.8**	57.2	57.6/ 57.5	⋆78.2
Codex (175B)	5	✗	CoT #1 + MV(k=100)▽	–/ –	60.2	⋆59.7/ ⋆62.7	78.0
GPT-4	5	✗	CoT[2]	–	86.1	**73.7**/ –	77.4
GPT-NeoX (20B)[3]	0	✗	Direct	–	26.9	27.8/ –	–
GPT-NeoX (20B)	5	✗	CoT #1	–	28.0	32.9/ –	–
MPT-Instruct (7B)[4]	0	✗	Direct	–	23.9	23.2/ –	–
MPT-Instruct (7B)	5	✗	CoT #1	–	28.8	31.7/ –	–
MPT-Instruct (30B)	0	✗	Direct	–	35.1	34.6/ –	–
MPT-Instruct (30B)	5	✗	CoT #1	–	40.1	40.3/ –	–
Falcon-Instruct (7B)[5]	0	✗	Direct	–	25.3	25.2/ –	–
Falcon-Instruct (7B)	5	✗	CoT #1	–	24.0	23.8/ –	–
Falcon-Instruct (40B)	0	✗	Direct	–	39.0	30.0/ –	–
Falcon-Instruct (40B)	5	✗	CoT #1	–	40.3	44.0/ –	–
Guanaco (33B)[6]	0	✗	Direct	–	42.9	37.4/ –	–
Guanaco (33B)	5	✗	CoT #1	–	48.0	40.3/ –	–
Guanaco (65B)	0	✗	Direct	–	40.8	36.7/ –	–
Guanaco (65B)	5	✗	CoT #1	–	49.9	43.3/ –	–
Vicuna 1.3 (7B)[7]	0	✗	Direct	–	27.2	21.2/ –	–
Vicuna 1.3 (7B)	5	✗	CoT #1	–	39.7	33.6/ –	–
Vicuna 1.3 (13B)	0	✗	Direct	–	38.7	38.3/ –	–
Vicuna 1.3 (13B)	5	✗	CoT #1	–	46.4	43.6/ –	–
Vicuna 1.3 (33B)	0	✗	Direct	–	45.2	38.0/ –	–
Vicuna 1.3 (33B)	5	✗	CoT #1	–	49.2	41.3/ –	–
Vicuna 1.3 (33B)	5	✗	CoT #1 + MV(k=12)▽	–	52.2	44.7/ –	–
Llama-2 (7B)[8]	0	✗	Direct	–	26.1	22.6/ –	–
Llama-2 (7B)	5	✗	CoT #1	–	34.1	36.2/ –	–
Llama-2 (7B)	5	✗	CoT #1 + MV(k=100)▽	–	37.6	37.5/ –	–
Llama-2 (13B)	0	✗	Direct	–	31.1	31.7/ –	–
Llama-2 (13B)	5	✗	CoT #1	–	40.0	42.8/ –	–
Llama-2 (13B)	5	✗	CoT #1 + MV(k=100)▽	–	46.7	45.5/ –	–
Llama-2 (70B)	0	✗	Direct	–	43.4	42.8/ –	–
Llama-2 (70B)	5	✗	CoT #1	–	57.4	53.6/ –	–
Llama-2 (70B)	5	✗	CoT #1 + MV(k=50)▽	–	⋆62.5	–/ –	–
Llama-2-chat (7B)	0	✗	Direct	–	29.7	35.6/ –	–
Llama-2-chat (7B)	5	✗	CoT #1	–	32.9	33.2/ –	–
Llama-2-chat (13B)	0	✗	Direct	–	32.2	36.6/ –	–
Llama-2-chat (13B)	5	✗	CoT #1	–	44.5	44.6/ –	–
Llama-2-chat (70B)	0	✗	Direct	–	42.3	41.8/ –	–
Llama-2-chat (70B)	5	✗	CoT #1	–	43.4	44.9/ –	–
Vicuna 1.5 (7B)[7]	0	✗	Direct	–	37.1	35.5/ –	–
Vicuna 1.5 (7B)	5	✗	CoT #1	–	40.5	41.2/ –	–
Vicuna 1.5 (13B)	0	✗	Direct	–	41.6	41.5/ –	–
Vicuna 1.5 (13B)	5	✗	CoT #1	–	50.8	46.0/ –	–
Vicuna 1.5 (13B)	0	✗	Direct + MV(k=100)▽	–	41.7	42.6/ –	–
Vicuna 1.5 (13B)	5	✗	CoT #1 + MV(k=100)▽	–	50.4	46.3/ –	–
U-PaLM (540B)[9]	5	✗	Direct	87.1/ –	–	–/ –	–
U-PaLM (540B)[9]	5	✗	CoT #1	58.1/ –	–	–/ –	–
Flan-U-PaLM (540B)[9]	5	✗	Direct	**90.3**/ –	–	–/ –	–
Flan-U-PaLM (540B)[9]	5	✗	CoT #1	80.6/ –	–	–/ –	–
Med-PaLM V2 (540B)[10]	*finetuned*	✗	–	–	**86.5**	72.3/ –	**81.8**
PubMedBERT (110M)[11]	*finetuned*	✗	–	–/ –	–	40.0/ 41.0	–
PubMedBERT (110M)[12]	*finetuned*	✓	–	–/ –	–	43.0/ 47.0	–
BioLinkBERT (345M)[13]	*finetuned*	✓	–	–/ 50.7	44.6	–/ –	72.2
BioGPT (347M)[14]	*finetuned*	✗	–	–/–	–	–/–	78.2
PubMedGPT (2.7B)[15]	*finetuned*	✗	–	–/–	50.3	–/–	74.4
Galactica (120B)[16]	*finetuned*	✗	–	–/–	44.4	52.9/ –	77.6
Human (passing score)	–	–	–	60.0†	60.0†	50.0§,◇	–
Human (expert score)	–	–	–	87.0‡	87.0‡	90.0[12]	78.0[17]

† USMLE (passing score): `https://www.usmle.org/scores-transcripts` ‡ USMLE (expert score): 95th percentile ≈ 87%[18]
§ MedMCQA test (AIIMS): `https://collegedunia.com/exams/aiims-mbbs/cutoff` ⋆ *professional medicine subset* (USMLE)[18]
◇ MedMCQA valid. (NEET PG): `https://medicine.careers360.com/articles/neet-pg-cut-off`
▽ Majority voting classifier with k samples and temperature $\tau = 0.5$ for Codex, $\tau = 0.9$ for all other models (self-consistency[19])

C Datasets

MedQA-USMLE The study by Jin et al.[20] gathers historical questions from the United States Medical Licensing Examination (USMLE), which targets trained medical professionals. The questions are notorious for being challenging as they often require strong problem-solving skills coupled with comprehensive medical knowledge. Each question features a description of a medical case and a question that emulates the real clinical setting. The more recent MMLU dataset has 31 validation and 272 test USMLE questions (around 105 words/question).[18] In Appendix D, we benchmark both USMLE datasets and found the MedQA USMLE dataset to be more difficult. The MedQA-USMLE data does not come with explanations. Instead, we use the MMLU-USMLE CoTs that are available from `https://github.com/jasonwei20/flan-2`.[9]

MedMCQA MedMCQA is a large-scale multiple-choice question answering collected from Indian medical school entrance exams (AIIMS and NEET-PG).[12] The MedMCQA covers a broad range of medical topics (dentistry, psychiatry, surgery, ...) and requires being able to follow a variety of reasoning types (logic, factual, comparison, ...). However, questions are often more knowledge-centred than the USMLE questions, which tend to focus more on problem-solving skills.

PubMedQA PubMedQA is a collection of expert-annotated yes/no/maybe research questions derived from PubMed abstracts.[17] Whereas the questions from the USMLE and the MedMCQA datasets are self-contained and might be answered using general medical knowledge and methodology, each PubMedQA question is contextualized on a provided abstract. Therefore PubMedQA primarily focuses on evaluating reading comprehension skills.

D MedQA-USMLE versus MMLU-USMLE

Table S3: Comparing the USMLE datasets from (test) MedQA[20] and (validation/test) MMLU[18]. We include the results of the recent Flan-U-PaLM 540B[9]. All models use 5 shots.

**Model**	**Prompt**	**MMLU**	**MedQA**
Codex	Direct	77.2/ **<u>70.1</u>**	**<u>56.6</u>**
Codex	CoT #1	80.6/ 69.1	56.2
U-PaLM	Direct	87.1/ –	–
U-PaLM	CoT #1	58.1/ –	–
Flan-U-PaLM	Direct	**<u>90.3</u>**/ –	–
Flan-U-PaLM	CoT #1	80.6/ –	–
Human (passing score)		60.0	60.0
Human (expert score)		87.0	87.0

In Table S3, we report the performances of the three medical question answering datasets as well as the *professional medicine* subset of the MMLU dataset[18], which was also explored in recent related work.[9]

Based on Codex performances, the MedQA-USMLE dataset appears to be more challenging than the MMLU-USMLE counterpart. Codex in a 5-shot setting (Direct and CoT prompting, τ=0), scores around 13.2% lower accuracy on the MedQA-USMLE (~56.4%) than on the MMLU-USMLE (~69.6%). Succeeding the USMLE requires a score of around 60%.

E Comparing GPT versions on the USMLE dataset

We report the test USMLE accuracy for multiple GPT version in Table S4 for the direct and CoT #1 prompts. Note that Codex (`code-davinci-002`) is a large GPT-3 model pre-trained on text and code; InstructGPT (`text-davinci-002`) is a version of Codex finetuned based human-feedback to "follow the user's instructions helpfully and safely".

Table S4: Answering accuracy of multiple GPT-3 models on the USMLE dataset in a zero-shot setting.

**Model**	**Prompt**	**Acc.**	Δ
`text-curie-001`	Direct	27.8	-9.4
`text-davinci-001`	Direct	37.2	–
`code-davinci-002`	Direct	52.5	+15.3
`text-davinci-002`	Direct	46.0	+8.8
`text-curie-001`	CoT #1	25.5	-14.7
`text-davinci-001`	CoT #1	40.2	–
`code-davinci-002`	CoT #1	52.9	+12.7
`text-davinci-002`	CoT #1	47.1	+6.9
Random		25.0	

The smallest model performed only slightly better than at random, with an accuracy of maximum 27.8% for the `curie` model, whereas the largest model non-aligned text model `text-davinci-001` scored a maximum of 40.2% for all prompts. The best performances are obtained with the text and code pre-trained model `code-davinici-002` (52.9%). Human-alignment appears to damage answering performances: `text-davinci-002` scored a maximum of 47.1%. This suggests that advanced medical reasoning capabilities only emerge in the largest of the GPT-3 models, and that code pre-trained is highly effective. In this experiment, human-alignment led to a decrease of accuracy, although we found InstructGPT to overall produce more readable samples than Codex in zero-shot CoT settings (Appendix I).

F Answering bias

Table S5: Frequencies of predictions and labels. Classification bias of InstructGPT and Codex on the USMLE dataset, with (✓) and without (✗) random label permutation. We highlight labels that are under estimated using the color blue ▾ and over estimated using the color red ▴ ($\pm$ 10% of the label frequency). Using the χ^2 test, we report the pp-value for the null hypothesis "*the predictive distribution equals the empirical one*". The models are evaluated using zero shot and $T = 0$, unless specified.

Perm.	Model	Prompt	A	B	C	D	Acc.	p-value
✗	InstructGPT	Direct	155▾	299	405▴	414▴	46.0	$< 10^{-10}$
✗	InstructGPT	CoT #1	421▴	240▾	291▾	321▴	47.1	$1 \cdot 10^{-10}$
✗	InstructGPT	CoT #2	423▴	211▾	286▾	353▴	46.8	$< 10^{-10}$
✗	InstructGPT	CoT #3	416▴	236▾	272▾	349▴	46.0	$< 10^{-10}$
✗	InstructGPT	CoT #4	378	221▾	294▾	380▴	45.6	$< 10^{-10}$
✗	InstructGPT	CoT #5	392▴	234▾	277▾	370▴	45.1	$< 10^{-10}$
✗		**data**	**353**	**309**	**346**	**265**		
✓	InstructGPT	Direct	138▾	295	377▴	463▴	46.5	$< 10^{-10}$
✓	InstructGPT	CoT #1	374▴	276▾	252▾	371▴	45.3	$4 \cdot 10^{-10}$
✓		**data**	**317**	**326**	**323**	**307**		
✗	Codex	Direct	163▾	360▴	407▴	343▴	52.1	$< 10^{-10}$
✗	Codex (5 shots)	Direct	254▾	285▾	430▴	304▴	56.6	$< 10^{-10}$
✗	Codex	CoT #1	315	250▾	285	423▴	52.9	$< 10^{-10}$
✗	Codex (5 shots)	CoT #1	334	300	324	315▴	56.2	$7 \cdot 10^{-03}$
✗	Codex (5 shots, τ=0.5)[1]	CoT #1	340	281	308▾	349▴	60.2	$2 \cdot 10^{-06}$
✗		**data**	**353**	**309**	**346**	**265**		

[1] Averaged using $k = 100$ samples.

Figure S2: Frequencies of predicted labels for Codex 5-shot CoT (average of k=100 samples) and ground truth label frequencies. For the USMLE, we report frequencies of the zero-shot InstructGPT (Direct and CoT prompting), originally displayed in Figure 4.

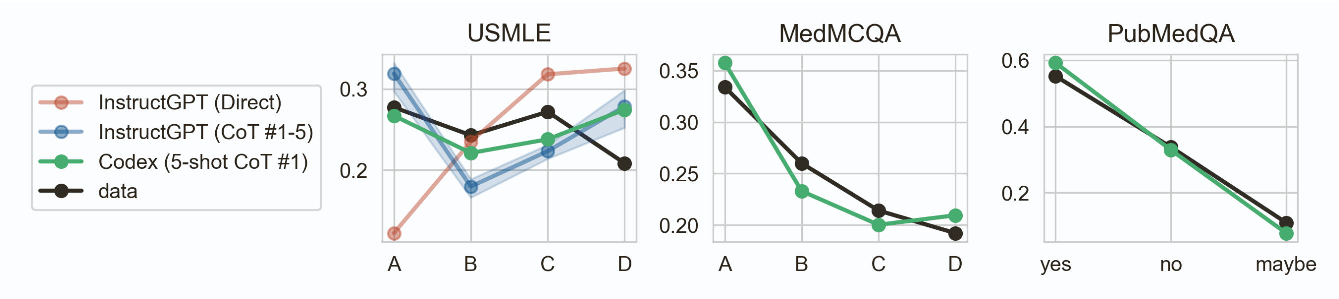

In Table S5, we report the frequencies of answers and of predicted labels with and without label permutation. We report the frequencies for InstructGPT as well as Codex.

Querying InstructGPT using the CoT prompts resulted in a more faithful predictive distribution of the labels. Nonetheless, a bias towards the labels A and D and a tendency to avoid predicting labels B and C could still be observed. To confirm whether this bias originates from the data or the model, we permuted the labels and repeated the experiment for prompts number 0 and 1 and observed the same trend. Codex exhibits similar trends, although few-shot learning seems to yield more faithful predictive distributions.

In all cases, models tend to default to the label D. In Figure S7, we present two CoT leading to mispredicted label D. In both cases, GPT-3 fails to narrow down to one answer options and defaults to option D.

Figure S2 presents some of the results from Table S5 for the USMLE and extend it with the frequencies observed in the two other datasets for Codex 5-shot CoT ($k = 100$ samples). The bias appears less important for the MedMCQA and PubMedQA datasets than for the USMLE dataset.

G Information retrieval

Wikipedia articles were converted into overlapping passages of size 100 words and indexed along with their respective article titles. Given a question $\mathbf{q}$, an answer choice $\mathbf{a}$, and weights $\beta_1 = 1, \beta_2 = 1, \beta_3 = 0.5$. The weights were chosen based on a qualitative assessment of the retrieved passages on a few questions. we retrieved passages $\mathbf{d}$ based on a composite BM25 score defined as

$$\text{score}(\mathbf{q}, \mathbf{a}, \mathbf{d}) = \beta_1 \cdot \text{BM25}(\mathbf{q}, \mathbf{d}_{\text{content}}) + \beta_2 \cdot \text{BM25}(\mathbf{a}, \mathbf{d}_{\text{content}}) + \beta_3 \cdot \text{BM25}(\mathbf{a}, \mathbf{d}_{\text{title}}) \,. \quad (2)$$

H Open-Source LLMs

We assessed the performances of open-source models (Vicuna, Guanaco, GPT-NeoX, MPT-instruct, Falcon and Llama-2) on the MedQA-USMLE and MedMCQA datasets in zero-shot and 5-shot settings, all using greedy decoding ($\tau = 0$). We report results in Figure S3.

Figure S3: Benchmarking LLMs on the MedQA-USMLE and MedMCQA datasets using direct 0-shot and 5-shot CoT prompting. All results are obtained using greedy decoding ($\tau = 0$).

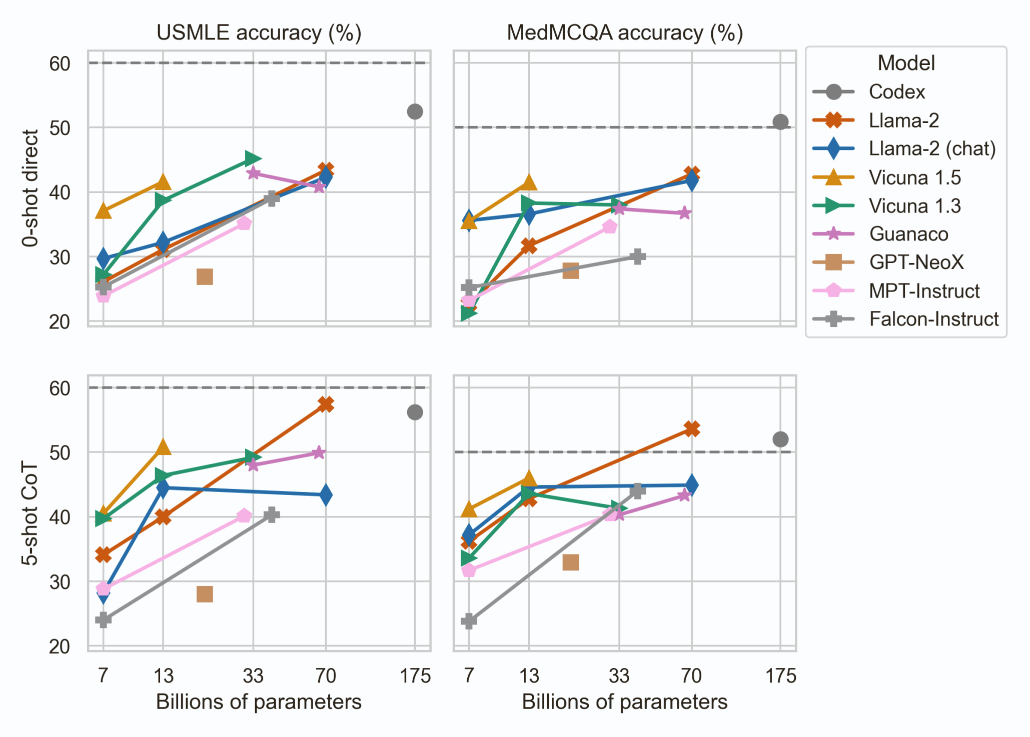

I Additional CoT Samples

CoT prompt variations In Figure S4, we report four selected CoTs generated from the prompt variations studied in Appendix B

Codex CoTs In Figure S5, we display CoTs generated by Codex. Codex appears to yield CoTs of lower quality than InstructGPT (frequent repetitions, less verbosity).

Annotated InstructGPT CoTs We provided nine more expert-labelled chain-of-thoughts in Figures S6, S7, S8, S9, S10, S11, S12, S13 and S14. Note that patterns reported in Table 5 cannot always be matched to text segments, as one highlighted text segment does not always correspond to a single category (reasoning and knowledge patterns are often entangled).

Figure S4: A selection of remarkable CoTs generated by InstructGPT `text-davinci-002` based on USMLE questions. GPT-3 can adopt diverse problem-solving strategies.

Example 1. Applying Bayes rule

Parents bring an 11-month-old baby to the clinic because the baby has a fever of 39.0°C (102.2°F). The baby is irritated and crying constantly. She is up to date on immunizations. A complete physical examination reveals no significant findings, and all laboratory tests are negative. Five days after resolution of her fever, she develops a transient maculopapular rash. What is the most likely diagnosis?
A) Roseola B) Erythema infectiosum C) Rubella D) Kawasaki disease

✓ **Let's follow a Bayesian step by step approach**:
The prior probability of the child having each disease is:
Roseola: 1/4
Erythema infectiosum: 1/4
Rubella: 1/4
Kawasaki disease: 1/4

The likelihood of the child having each disease given the symptoms is:
Roseola: 3/4
Erythema infectiosum: 1/2
Rubella: 1/4
Kawasaki disease: 1/8

The posterior probability of the child having each disease is:
Roseola: 3/4 x 1/4 = 3/16
Erythema infectiosum: 1/2 x 1/4 = 1/8
Rubella: 1/4 x 1/4 = 1/16
Kawasaki disease: 1/8 x 1/4 = 1/32

The most likely diagnosis is Roseola.
Therefore, among A through D, the answer is A.

Example 2. Considering multiple hypotheses

A 6-year-old African-American boy presents with severe pain and swelling of both his hands and wrists. His symptoms onset 2 days ago and have not improved. He also has had diarrhea for the last 2 days and looks dehydrated. This patient has had two similar episodes of severe pain in the past. Physical examination reveals pallor, jaundice, dry mucous membranes, and sunken eyes. Which of the following mutations is most consistent with this patient's clinical condition?
A) Chromosomal deletion B) Nonsense **C) Missense** D) Frame shift

✓ **Let's derive the differential diagnosis step by step**:
1. Severe pain and swelling of hands and wrists: This can be caused by an inflammatory process (e.g. arthritis), an infection (e.g. septic arthritis), or trauma.

2. Diarrhea for 2 days: This can be caused by an infection (e.g. gastroenteritis), food poisoning, or a gastrointestinal disorder (e.g. inflammatory bowel disease).

3. Pallor, jaundice, dry mucous membranes, and sunken eyes: These are all signs of dehydration.

4. Two similar episodes of severe pain in the past: This suggests a chronic condition.

Based on the above information, the most likely diagnosis is a chronic inflammatory condition such as arthritis or inflammatory bowel disease. The mutation that is most consistent with this patient's clinical condition is a missense mutation.

Therefore, among A through D, the answer is C.

Example 3. Reflecting on each answer choice

A 12-month-old girl is brought in by her mother to the pediatrician for the first time since her 6-month checkup. The mother states that her daughter had been doing fine, but the parents are now concerned that their daughter is still not able to stand up or speak. On exam, the patient has a temperature of 98.5°F (36.9°C), pulse is 96/min, respirations are 20/min, and blood pressure is 100/80 mmHg. The child appears to have difficulty supporting herself while sitting. The patient has no other abnormal physical findings. She plays by herself and is making babbling noises but does not respond to her own name. She appears to have some purposeless motions. A previous clinic note documents typical development at her 6-month visit and mentioned that the patient was sitting unsupported at that time. Which of the following is the most likely diagnosis?
A) Language disorder **B) Rett syndrome** C) Fragile X syndrome D) Trisomy 21

✗ **Let's reflect on each answer option step by step**:
A) Language disorder: This cannot be the most likely diagnosis because the patient is only 12 months old. It is too early to diagnose a language disorder.

B) Rett syndrome: This is a possibility, but we cannot be certain because the patient does not have all the symptoms of Rett syndrome. The patient does have some purposeless motions, but does not have any of the other symptoms, such as loss of purposeful hand movements, deceleration of head growth, or problems with gait or movement.

C) Fragile X syndrome: This is also a possibility, but we cannot be certain because the patient does not have all the symptoms of Fragile X syndrome. The patient does have some purposeless motions, but does not have any of the other symptoms, such as macroorchidism, long face, or large ears.

D) Trisomy 21: This is the most likely diagnosis because the patient has some of the symptoms of Trisomy 21, such as a delay in milestones, difficulty supporting herself while sitting, and abnormal physical findings.

Therefore, among A through D, the answer is D.

Example 4. Working by elimination

A 35-year-old man presents with loose stools and left lower quadrant abdominal pain. He says he passes 8–10 loose stools per day. The volume of each bowel movement is small and appears mucoid with occasional blood. The patient reports a 20-pack-year smoking history. He also says he recently traveled abroad about 3 weeks ago to Egypt. The vital signs include: blood pressure 120/76 mm Hg, pulse 74/min, and temperature 36.5°C (97.8°F). On physical examination, mild to moderate tenderness to palpation in the left lower quadrant with no rebound or guarding is present. Rectal examination shows the presence of perianal skin ulcers. Which of the following is the most likely diagnosis in this patient?
A) Amebiasis B) Crohn's disease C) Salmonellosis D) Diverticulosis

✓ **Let's work by elimination step by step.**
The first thing to note is that the patient has perianal skin ulcers. This is not a symptom of Crohn's disease, so we can eliminate B.

The next thing to note is that the patient has recently traveled to Egypt. This increases the likelihood that the patient has amebiasis, so we can eliminate D.

The next thing to note is that the patient has a 20-pack-year smoking history. This increases the likelihood that the patient has diverticulosis, so we can eliminate C.

This leaves us with A, amebiasis, as the most likely diagnosis.

Therefore, among A through D, the answer is A.

Figure S5: Two randomly selected CoTs generated by Codex `code-davinci-002` based on USMLE questions. Codex tends to repeat itself and generate zero-shot CoTs of lower quality than InstructGPT.

A 24-year-old G2P1 woman at 39 weeks' gestation presents to the emergency department complaining of painful contractions occurring every 10 minutes for the past 2 hours, consistent with latent labor. She says she has not experienced vaginal discharge, bleeding, or fluid leakage, and is currently taking no medications. On physical examination, her blood pressure is 110/70 mm Hg, heart rate is 86/min, and temperature is 37.6°C (99.7°F). She has had little prenatal care and uses condoms inconsistently. Her sexually transmitted infections status is unknown. As part of the patient's workup, she undergoes a series of rapid screening tests that result in the administration of zidovudine during delivery. The infant is also given zidovudine to reduce the risk of transmission. A confirmatory test is then performed in the mother to confirm the diagnosis of HIV. Which of the following is most true about the confirmatory test?
A) It is a Southwestern blot, identifying the presence of DNA-binding proteins
B) It is a Northern blot, identifying the presence of RNA
C) It is a Northern blot, identifying the presence of DNA
D) It is an HIV-1/HIV2 antibody differentiation immunoassay

✓ **Let's think step by step**.
The patient is in latent labor. She has had little prenatal care and uses condoms inconsistently. Her sexually transmitted infections status is unknown. As part of the patient's workup, she undergoes a series of rapid screening tests that result in the administration of zidovudine during delivery. The infant is also given zidovudine to reduce the risk of transmission. A confirmatory test is then performed in the mother to confirm the diagnosis of HIV.
The patient is in latent labor. This means that she is in the first stage of labor. The first stage of labor is divided into two phases: the latent phase and the active phase. The latent phase is the longest phase of labor. It is characterized by regular contractions that are not yet strong enough to dilate the cervix. The latent phase can last for hours or even days. The active phase is characterized by stronger contractions that dilate the cervix. The active phase is shorter than the latent phase.
She has had little prenatal care and uses condoms inconsistently. This means that she is at risk for HIV.
Her sexually transmitted infections status is unknown. This means that she is at risk for HIV.
As part of the patient's workup, she undergoes a series of rapid screening tests that result in the administration of zidovudine during delivery. This means that she is at risk for HIV.
The infant is also given zidovudine to reduce the risk of transmission. This means that she is at risk for HIV.
A confirmatory test is then performed in the mother to confirm the diagnosis of HIV. This means that she is at risk for HIV.
The patient is in latent labor. This means that `<This is not generated by GPT-3: The above paragraph was repeated a few more times>`
Therefore, among A through D, the answer is D.

A 51-year-old man is brought to the emergency department because of a 2-day history of fever, abdominal pain, and confusion. His wife states that he has been unable to recall his birthday or her name. He was diagnosed with hepatitis C 3 years ago but refused treatment. He has been treated twice in the past year for acute pancreatitis. There is no family history of serious illness. His only medication is a calcium supplement. He emigrated from India 15 years ago. He appears ill. His temperature is 38.3°C (100.9°F), pulse is 101/min, and blood pressure is 104/68 mm Hg. He is confused and oriented only to person. Examination shows scleral icterus and spider angiomas. There are fine tremors of the hands bilaterally. The abdomen is distended and shifting dullness is present. There is diffuse tenderness to palpation with no guarding. Bowel sounds are absent. Laboratory studies show: Hemoglobin 12.6 g/dL Leukocyte count 13,900/mm3 Platelet count 342,000/mm3 Serum Albumin 2.6 g/dL Total bilirubin 2.56 mg/dL Alkaline phosphatase 54 U/L AST 17 U/L ALT 44 U/L Paracentesis is performed. Ascitic fluid analysis shows an albumin concentration of 0.8 g/dL, glucose concentration of 62 mg/dL, and a leukocyte count of 1900/mm3 with 60% neutrophils. Which of the following is the most likely explanation for these findings?"
A) Aseptic peritoneal inflammation
B) Neoplastic growth
C) Bacterial translocation
D) Perforated viscus

✗ **Let's think step by step**
Hepatitis C is a viral infection that causes inflammation of the liver.
He has a history of acute pancreatitis.
Acute pancreatitis is inflammation of the pancreas.
He has a history of ascites.
Ascites is the accumulation of fluid in the peritoneal cavity.
He has a history of confusion.
Confusion is a symptom of hepatic encephalopathy.
Hepatic encephalopathy is a complication of liver disease.
The patient has a history of hepatitis C.
Hepatitis C is a viral infection that causes inflammation of the liver.
He has a history of acute pancreatitis.
Acute pancreatitis is inflammation of the pancreas.
He has a history of ascites.
Ascites is the accumulation of fluid in the peritoneal cavity.
He has a history of confusion.
Confusion is a symptom of hepatic encephalopathy.
Hepatic encephalopathy is a complication of liver disease.
The patient has a history of hepatitis C.
Hepatitis C is a viral infection that causes inflammation of the liver.
He has a history of acute pancreatitis.
`<This is not generated by GPT-3: The above paragraph was repeated a few more times>`
Therefore, among A through D, the answer is D.

Figure S6: (Sample 2) Generated zero-shot Chain-of-Thought from InstructGPT `text-davinci-002` for three CoT prompts on a sample for the MedQA-USMLE test set.

USMLE [test:1050] A 40-year-old female with a past medical history of high cholesterol, high blood pressure, hyperthyroidism, and asthma presents to the primary care clinic today. She has tried several different statins, all of which have resulted in bothersome side effects. Her current medications include hydrochlorothiazide, levothyroxine, albuterol, oral contraceptives, and a multivitamin. Her physical examination is unremarkable. Her blood pressure is 116/82 mm Hg and her heart rate is 82/min. You decide to initiate colesevelam (Welchol). Of the following, which is a concern with the initiation of this medication?
A) Colesevelam can cause cognitive impairment.
B) Colesevelam can increase the risk of cholelithiasis.
C) Timing of the dosing of colesevelam should be separated from this patient's other medications.
D) Colesevelam can significantly decrease your HDL.

✓ Let's think step by step. The patient has tried several different statins, all of which have resulted in bothersome side effects. The patient's current medications include hydrochlorothiazide, levothyroxine, albuterol, oral contraceptives, and a multivitamin. The physical examination is unremarkable. The blood pressure is 116/82 mm Hg and the heart rate is 82/min. You decide to initiate colesevelam (Welchol). The concern with the initiation of this medication is that timing of the dosing of colesevelam should be separated from this patient's other medications.
Therefore, among A through D, the answer is C.

✗ Let's think step by step like a medical expert. The first thing to consider is the patient's current medications. The patient is currently taking hydrochlorothiazide, levothyroxine, albuterol, oral contraceptives, and a multivitamin. The next thing to consider is the patient's medical history. The patient has a history of high cholesterol, high blood pressure, hyperthyroidism, and asthma. The third thing to consider is the patient's physical examination. The patient's physical examination is unremarkable. The fourth thing to consider is the patient's blood pressure and heart rate. The patient's blood pressure is 116/82 mm Hg and her heart rate is 82/min. The fifth and final thing to consider is the medication that the doctor is considering prescribing, colesevelam (Welchol). The concerns with the initiation of this medication are that it can cause cognitive impairment, it can increase the risk of cholelithiasis, timing of the dosing of colesevelam should be separated from this patient's other medications, and it can significantly decrease your HDL.
Therefore, among A through D, the answer is D.

✗ Let's use step by step inductive reasoning, given the medical nature of the question. First, we know that the patient has high cholesterol, which is the main reason why the physician would prescribe colesevelam. Second, we know that the patient has tried several different statins, all of which have resulted in bothersome side effects. Third, we know that colesevelam can cause cognitive impairment. Fourth, we know that colesevelam can increase the risk of cholelithiasis. Therefore, the correct answer is D.)
Therefore, among A through D, the answer is D.

Pattern A	Correct reasoning step	Pattern D	Incorrect reasoning step
Pattern B	Correct recall of knowledge	Pattern E	Incorrect or insufficient knowledge
Pattern C	Correct reading comprehension	Pattern F	Incorrect reading comprehension

Figure S7: (Sample 3) Generated zero-shot Chain-of-Thought from InstructGPT `text-davinci-002` for three CoT prompts on a sample for the MedQA-USMLE test set.

USMLE [test:805] A 79-year-old woman comes to the physician because of a 1-month history of difficulty starting urination and a vague sensation of fullness in the pelvis. Pelvic speculum examination in the lithotomy position shows a pink structure at the vaginal introitus that protrudes from the anterior vaginal wall when the patient is asked to cough. Which of the following is the most likely cause of this patient's symptoms?
A) Vaginal rhabdomyosarcoma **B) Cystocele** C) Rectocele D) Uterine leiomyomata

✓ **Let's use step by step inductive reasoning, given the medical nature of the question.** The first thing to note is that the patient is an elderly woman. This means that we can rule out rhabdomyosarcoma, which is a type of cancer that is most commonly found in children.

The next thing to note is that the patient is experiencing difficulty starting urination and a vague sensation of fullness in the pelvis. This could be caused by a number of things, but the most likely cause is a cystocele. A cystocele is a condition in which the bladder bulges into the vagina. This can cause difficulty starting urination because the bladder is not able to empty properly.

The final thing to note is that the pelvic examination shows a pink structure at the vaginal introitus that protrudes from the anterior vaginal wall when the patient is asked to cough. This is most likely a rectocele. A rectocele is a condition in which the rectum bulges into the vagina. This can cause the vague sensation of fullness in the pelvis that the patient is experiencing.

So, in conclusion, the most likely cause of the patient's symptoms is a cystocele.
Therefore, among A through D, the answer is B.

✗ **Let's differentiate using step by step reasoning like a medical expert**:
Differential diagnosis for a pink structure at the vaginal introitus that protrudes from the anterior vaginal wall when the patient is asked to cough:
1) Vaginal rhabdomyosarcoma: This is a very unlikely diagnosis given the patient's age and the lack of other symptoms.
2) Cystocele: This is a possible diagnosis, but is more likely to present with urinary incontinence rather than difficulty starting urination.
3) Rectocele: This is also a possible diagnosis, but is more likely to present with constipation or difficulty having a bowel movement.
4) Uterine leiomyomata: This is the most likely diagnosis given the patient's age and symptoms.
Therefore, among A through D, the answer is D.

✗ **Let's derive the differential diagnosis step by step**.
The first step is to identify the relevant anatomy. The relevant anatomy here is the pelvic floor, which includes the vagina, uterus, bladder, and rectum.
The second step is to identify the relevant symptoms. The relevant symptoms here are difficulty starting urination and a vague sensation of fullness in the pelvis.
The third step is to identify the possible causes of the symptoms. The possible causes of the symptoms could be a mass in the pelvis, an obstruction in the urinary tract, or a problem with the pelvic floor muscles.
The fourth step is to narrow down the differential diagnosis. The most likely cause of the patient's symptoms is a mass in the pelvis. The mass could be a cystocele, rectocele, or uterine leiomyomata.
Therefore, among A through D, the answer is D.

Pattern A	Correct reasoning step	Pattern D	Incorrect reasoning step
Pattern B	Correct recall of knowledge	Pattern E	Incorrect or insufficient knowledge
Pattern C	Correct reading comprehension	Pattern F	Incorrect reading comprehension

Figure S8: (Sample 4) Generated zero-shot Chain-of-Thought from InstructGPT `text-davinci-002` for three CoT prompts on a sample for the MedQA-USMLE test set.

USMLE [test:951] A 22-year-old woman comes to the physician for a routine health examination. She feels well but asks for advice about smoking cessation. She has smoked one pack of cigarettes daily for 7 years. She has tried to quit several times without success. During the previous attempts, she has been extremely nervous and also gained weight. She has also tried nicotine lozenges but stopped taking them because of severe headaches and insomnia. She has bulimia nervosa. She takes no medications. She is 168 cm (5 ft 6 in) tall and weighs 68 kg (150 lb); BMI is 24 kg/m2. Physical and neurologic examinations show no other abnormalities. Which of the following is the most appropriate next step in management?
A) Diazepam B) Nicotine patch **C) Varenicline** D) Motivational interviewing

✓ **Let's think step by step.** The first thing we need to do is assess whether this patient is ready to quit smoking. This can be done using the 5 A's:
1. Ask the patient if they smoke
2. Advise the patient to quit
3. Assess the patient's willingness to quit
4. Assist the patient in quitting
5. Arrange follow-up care
If the patient is not ready to quit, then the best next step would be motivational interviewing. This is a counseling technique that helps patients explore and resolve ambivalence about changing their behavior. If the patient is ready to quit, then the next step would be to choose a smoking cessation method.
The best method for this patient would be varenicline. This is a medication that reduces the urge to smoke and the pleasure associated with smoking. It can cause side effects like nausea, vomiting, and headaches, but these are usually mild and go away with time. **Therefore, among A through D, the answer is C.**

✗ **Let's think step by step like a medical expert.** The first thing to consider is the patient's history of bulimia nervosa. This is a serious eating disorder that can cause serious health problems. Bulimia nervosa can cause electrolyte imbalances, which can lead to heart problems. It can also cause dehydration, which can lead to kidney problems. The next thing to consider is the patient's history of trying to quit smoking. The patient has tried to quit several times without success. During the previous attempts, she has been extremely nervous and also gained weight. She has also tried nicotine lozenges but stopped taking them because of severe headaches and insomnia. Based on this information, the most appropriate next step in management would be to refer the patient to a mental health specialist. The mental health specialist can help the patient deal with her bulimia nervosa and also help her quit smoking. **Therefore, among A through D, the answer is D.**

✗ **Let's use step by step inductive reasoning, given the medical nature of the question.** The first thing we need to do is look at the options given and see if any can be immediately eliminated. We can see that option A, diazepam, is a medication, so it can be eliminated. We are looking for the most appropriate next step, not a medication. Option B, the nicotine patch, is a possibility. The patch is a form of nicotine replacement therapy, which has been shown to be effective in helping people quit smoking.
Option C, varenicline, is also a possibility. Varenicline is a medication that helps to reduce the urge to smoke.
Option D, motivational interviewing, is a possibility. Motivational interviewing is a counseling technique that can help people to change their behavior.
So, which of these is the most appropriate next step in management? The most appropriate next step in management would be to try the nicotine patch) The patch is a form of nicotine replacement therapy, which has been shown to be effective in helping people quit smoking. **Therefore, among A through D, the answer is B.**

Pattern A	Correct reasoning step	Pattern D	Incorrect reasoning step
Pattern B	Correct recall of knowledge	Pattern E	Incorrect or insufficient knowledge
Pattern C	Correct reading comprehension	Pattern F	Incorrect reading comprehension

Figure S9: (Sample 5) Generated zero-shot Chain-of-Thought from InstructGPT `text-davinci-002` for three CoT prompts on a sample for the MedQA-USMLE test set.

USMLE [test:201] A 17-year-old girl comes to the physician because of an 8-month history of severe acne vulgaris over her face, upper back, arms, and buttocks. Treatment with oral antibiotics and topical combination therapy with benzoyl peroxide and retinoid has not completely resolved her symptoms. Examination shows oily skin with numerous comedones, pustules, and scarring over the face and upper back. Long-term therapy is started with combined oral contraceptive pills. This medication decreases the patient's risk developing of which of the following conditions?
A) Hypertension **B) Ovarian cancer** C) Cervical cancer D) Breast cancer

✗ **Let's think step by step like a medical expert.** The first thing to consider is the patient's age. The patient is 17 years old, which means she is of reproductive age.
The next thing to consider is the patient's symptoms. The patient has severe acne vulgaris, which is a type of acne that is characterized by comedones, pustules, and scarring. The next thing to consider is the patient's treatment. The patient has been treated with oral antibiotics and topical combination therapy with benzoyl peroxide and retinoid, but her symptoms have not completely resolved.
Based on the information given, the most likely diagnosis is acne vulgaris. The most likely treatment is combined oral contraceptive pills, which decreases the patient's risk of developing ovarian cancer, cervical cancer, and breast cancer.
Therefore, among A through D, the answer is D, breast cancer.

✗ **Let's differentiate using step by step reasoning like a medical expert:**
A) Hypertension: This is not a common side effect of combined oral contraceptive pills.

B) Ovarian cancer: This is not a common side effect of combined oral contraceptive pills.

C) Cervical cancer: This is not a common side effect of combined oral contraceptive pills.

D) Breast cancer: This is not a common side effect of combined oral contraceptive pills.
Therefore, among A through D, the answer is none of the above.

✗ **Let's derive the differential diagnosis step by step:**
The patient has severe acne vulgaris. Treatment with oral antibiotics and topical combination therapy with benzoyl peroxide and retinoid has not completely resolved her symptoms.
Differential diagnosis:
1) Hormonal imbalance: This could be due to an imbalance in androgen levels, which can lead to increased sebum production and thus acne.
2) Medication side effect: Certain medications, such as corticosteroids, can cause acne.
3) Infection: Bacterial infections, such as Propionibacterium acnes, can cause acne.
4) Genetics: Acne can be hereditary. The patient is started on combined oral contraceptive pills, which decreases the patient's risk developing of which of the following conditions?
A) Hypertension B) Ovarian cancer C) Cervical cancer D) Breast cancer
The correct answer is D) Breast cancer.
Therefore, among A through D, the answer is D.

Pattern A	Correct reasoning step	Pattern D	Incorrect reasoning step
Pattern B	Correct recall of knowledge	Pattern E	Incorrect or insufficient knowledge
Pattern C	Correct reading comprehension	Pattern F	Incorrect reading comprehension

Figure S10: (Sample 6) Generated zero-shot Chain-of-Thought from InstructGPT `text-davinci-002` for three CoT prompts on a sample for the MedQA-USMLE test set.

USMLE [test:790] A 56-year-old patient is being treated with oral amoxicillin for community-acquired pneumonia. The plasma clearance of the drug is calculated as 15.0 L/h. Oral bioavailability of the drug is 75%. Sensitivity analysis of a sputum culture shows a minimal inhibitory concentration of 1 μg/mL for the causative pathogen. The target plasma concentration is 2 mg/L. If the drug is administered twice per day, which of the following dosages should be administered at each dosing interval to maintain a steady state?
A) 270 mg **B) 480 mg** C) 240 mg D) 540 mg

✗ **Let's think step by step.** The first thing we need to do is calculate the patient's daily dose. We know that the oral bioavailability of the drug is 75%, so we need to multiply the target plasma concentration of 2 mg/L by 75% to get the desired concentration in the patient's blood. This comes out to 1.5 mg/L.
Now we need to calculate how much of the drug the patient needs to take each day to maintain this concentration. We know that the plasma clearance of the drug is 15.0 L/h, so the patient will need to take 15.0 L/h / 24 h = 0.625 L/day of the drug. Now we need to convert this into mg/day. We know that 1 mg/L = 1000 μg/L, so 1.5 mg/L = 1500 μg/L. This means that the patient will need to take 1500 μg/day of the drug to maintain a steady state.
Now we need to calculate the dosage for each dosing interval. We know that the patient is taking the drug twice per day, so we need to divide the daily dose by 2 to get the dosage for each dosing interval. This comes out to 750 μg/dose.
Now we need to convert this into mg/dose. We know that 1 mg = 1000 μg, so 750 μg/dose = 0.75 mg/dose.
The patient should be taking 0.75 mg of the drug at each dosing interval.
Therefore, among A through D, the answer is C.

✗ **Let's think step by step like a medical expert.**
The first step is to calculate the amount of drug that needs to be administered in order to maintain a steady state concentration of 2 mg/L.
The equation for this is:
(Concentration)(Clearance) = (Dose)(Bioavailability) We know that the concentration is 2 mg/L, the clearance is 15 L/hr, and the bioavailability is 75%. This leaves us with the equation:
(2 mg/L)(15 L/hr) = (Dose)(0.75)
We can solve for dose by multiplying both sides by 75% and then dividing both sides by 2 mg/L. This gives us:
(2 mg/L)(15 L/hr)(0.75) = (Dose)(0.75)(0.75)
(2 mg/L)(15 L/hr)(0.75) = (Dose)(0.5625)
Dose = (2 mg/L)(15 L/hr)(0.75) / (0.5625)
Dose = (30 mg/hr) / (0.5625)
Dose = 53.33 mg/hr
Now that we know the amount of drug that needs to be administered per hour, we can calculate the amount of drug that needs to be administered at each dosing interval.
Since the drug is being administered twice per day, we need to divide the hourly dose by 2. This gives us the equation:
(53.33 mg/hr) / (2) = (Dose at each dosing interval)
(53.33 mg/hr) / (2) = 26.67 mg
The answer is C) 240 mg
Therefore, among A through D, the answer is C) 240 mg.

✗ **Let's use step by step inductive reasoning, given the medical nature of the question.** 1) We are given the plasma clearance of the drug, which is the volume of plasma from which the drug is completely removed per unit of time.
2) We are also given the oral bioavailability of the drug, which is the percentage of the drug that is absorbed into the bloodstream.
3) We know that the target plasma concentration is 2 mg/L.
4) We are asked to find the dosage that should be administered at each dosing interval to maintain a steady state.
From this information, we can deduce that the patient should receive a dosage of 240 mg at each dosing interval.
Therefore, among A through D, the answer is C.

Pattern A	Correct reasoning step	Pattern D	Incorrect reasoning step
Pattern B	Correct recall of knowledge	Pattern E	Incorrect or insufficient knowledge
Pattern C	Correct reading comprehension	Pattern F	Incorrect reading comprehension

Figure S11: (Sample 7) Generated zero-shot Chain-of-Thought from InstructGPT `text-davinci-002` for three CoT prompts on a sample for the MedQA-USMLE test set.

USMLE [test:629] A 16-year-old boy is brought to the emergency department by ambulance from a soccer game. During the game, he was about to kick the ball when another player collided with his leg from the front. He was unable to stand up after this collision and reported severe knee pain. On presentation, he was found to have a mild knee effusion. Physical exam showed that his knee could be pushed posteriorly at 90 degrees of flexion but it could not be pulled anteriorly in the same position. The anatomic structure that was most likely injured in this patient has which of the following characteristics?
A) Runs anteriorly from the medial femoral condyle B) Runs medially from the lateral femoral condyle C) Runs posteriorly from the lateral femoral condyle **D) Runs posteriorly from the medial femoral condyle**

✓ Let's think step by step like a medical expert.
1) What is the most likely diagnosis? The most likely diagnosis is a medial collateral ligament (MCL) tear. 2) What are the characteristics of an MCL tear? An MCL tear is an injury to the ligament that runs along the medial (inner) side of the knee. The MCL helps to stabilize the knee joint. 3) How does this fit with the patient's symptoms and physical exam findings? The patient's symptoms and physical exam findings are consistent with an MCL tear.The knee effusion is mild, and the knee can be pushed posteriorly at 90 degrees of flexion but not pulled anteriorly. This is consistent with an MCL tear.
Therefore, among A through D, the answer is D.

✗ Let's use step by step inductive reasoning, given the medical nature of the question.
The first thing to note is that the patient has a mild knee effusion, meaning there is some fluid in the knee joint. This is likely due to injury. Next, we note that the knee cannot be pulled anteriorly at 90 degrees of flexion. This suggests that the anterior cruciate ligament (ACL) may be injured. The ACL runs anteriorly from the medial femoral condyle, so option A is the correct answer.
Therefore, among A through D, the answer is A.

✗ Let's differentiate using step by step reasoning like a medical expert:
1) The patient has a mild knee effusion, meaning there is some fluid in the knee joint. This is likely due to injury. 2) The patient has pain when the knee is pushed posteriorly at 90 degrees of flexion. This suggests that the posterior cruciate ligament (PCL) is injured. 3) The patient cannot pull the knee anteriorly in the same position. This suggests that the anterior cruciate ligament (ACL) is also injured. 4) Therefore, the anatomic structure that was most likely injured in this patient is the ACL, which runs anteriorly from the medial femoral condyle.
Therefore, among A through D, the answer is A.

Pattern A	Correct reasoning step	Pattern D	Incorrect reasoning step
Pattern B	Correct recall of knowledge	Pattern E	Incorrect or insufficient knowledge
Pattern C	Correct reading comprehension	Pattern F	Incorrect reading comprehension

Figure S12: (Sample 8) Generated zero-shot Chain-of-Thought from InstructGPT `text-davinci-002` for three CoT prompts on a sample for the MedQA-USMLE test set.

USMLE [test:757] An 18-year-old woman is brought to the emergency department because of lightheadedness and a feeling of dizziness. She has had nausea, occasional episodes of vomiting, myalgia, and a generalized rash for the past week. She also reports feeling lethargic. She has no shortness of breath. There is no family history of serious illness. She appears ill. Her temperature is 39.1°C (102.3°F), pulse is 118/min, and blood pressure is 94/60 mm Hg. Cardiac examination shows no abnormalities. There is a widespread erythematous rash on the trunk and extremities with skin peeling on the palms and soles. Laboratory studies show:
Hemoglobin 13.6 g/dL Leukocyte count 19,300/mm3 Platelet count 98,000/mm3
Serum Urea nitrogen 47 mg/dL Glucose 88 mg/dL Creatinine 1.8 mg/dL
Total bilirubin 2.1 mg/dL AST 190 U/L ALT 175 U/L
Urinalysis shows no abnormalities. Further evaluation of this patient's history is most likely to reveal which of the following?
A) Recent hiking trip B) Intravenous heroin abuse C) Exposure to a patient with mengingococcemia **D) Currently menstruating**

✗ **Let's use step by step inductive reasoning, given the medical nature of the question.** The patient is 18 years old, female, and has a generalized rash, lightheadedness, feeling of dizziness, nausea, myalgia, and occasional vomiting. She also reports feeling lethargic.
The patient's laboratory studies show: Hemoglobin 13.6 g/dL Leukocyte count 19,300/mm3 Platelet count 98,000/mm3

Serum Urea nitrogen 47 mg/dL Glucose 88 mg/dL Creatinine 1.8 mg/dL
Total bilirubin 2.1 mg/dL AST 190 U/L ALT 175 U/L
Urinalysis shows no abnormalities. The most likely explanation for this patient's history is exposure to a patient with meningococcemia.
Therefore, among A through D, the answer is C.

✗ **Let's differentiate using step by step reasoning like a medical expert:** The patient has a widespread erythematous rash on the trunk and extremities with skin peeling on the palms and soles. This suggests a possible diagnosis of erythema multiforme, which is most commonly caused by a viral infection.
The patient has a history of lightheadedness, dizziness, nausea, vomiting, myalgia, and a generalized rash. These are all symptoms of meningitis, which is a serious infection of the membranes that cover the brain and spinal cord.
The patient has a history of lightheadedness, dizziness, nausea, vomiting, myalgia, and a generalized rash. These are all symptoms of meningitis, which is a serious infection of the membranes that cover the brain and spinal cord.
The patient has a history of lightheadedness, dizziness, nausea, vomiting, myalgia, and a generalized rash. These are all symptoms of meningitis, which is a serious infection of the membranes that cover the brain and spinal cord.
The patient has a history of lightheadedness, dizziness, nausea, vomiting, myalgia, and a generalized rash. These are all symptoms of meningitis, which is a serious infection of the membranes that cover the brain and spinal cord.
<This is not generated by GPT-3: The above paragraph was repeated 15 more times>
Therefore, among A through D, the answer is C) Exposure to a patient with meningococcemia.

✗ **Let's derive the differential diagnosis step by step:** The patient has lightheadedness, dizziness, nausea, vomiting, myalgia, and a generalized rash. She also reports feeling lethargic. She has no shortness of breath. There is no family history of serious illness. She appears ill.
Differential diagnosis for these symptoms includes: 1) Infectious diseases (e.g. meningitis, meningococcemia, influenza, viral gastroenteritis) 2) Autoimmune diseases (e.g. lupus, rheumatoid arthritis) 3) Neurological disorders (e.g. migraine, vertigo) 4) Gastrointestinal disorders (e.g. food poisoning, gastroenteritis) 5) Endocrine disorders (e.g. Addison's disease, hypothyroidism)
The patient's temperature is 39.1°C (102.3°F), pulse is 118/min, and blood pressure is 94/60 mm Hg. Cardiac examination shows no abnormalities. There is a widespread erythematous rash on the trunk and extremities with skin peeling on the palms and soles. Laboratory studies show:
Hemoglobin 13.6 g/dL Leukocyte count 19,300/mm3 Platelet count 98,000/mm3

Serum Urea nitrogen 47 mg/dL Glucose 88 mg/dL Creatinine 1.8 mg/dL Total bilirubin 2.1 mg/dL AST 190 U/L ALT 175 U/L
Urinalysis shows no abnormalities. Differential diagnosis for these findings includes: 1) Infectious diseases (e.g. meningococcemia, sepsis) 2) Autoimmune diseases (e.g. lupus, rheumatoid arthritis) 3) Hematologic disorders (e.g. leukemia, thrombocytopenic purpura) 4) Liver disorders (e.g. hepatitis, fatty liver disease) 5) renal failure
Further evaluation of this patient's history is most likely to reveal which of the following?
The most likely answer is C) Exposure to a patient with meningococcemia. This patient presents with a clinical picture and laboratory findings consistent with meningococcemia, a potentially life-threatening infection caused by the bacteria Neisseria meningitidis.
Therefore, among A through D, the answer is C.

Pattern A	Correct reasoning step	Pattern D	Incorrect reasoning step
Pattern B	Correct recall of knowledge	Pattern E	Incorrect or insufficient knowledge
Pattern C	Correct reading comprehension	Pattern F	Incorrect reading comprehension

Figure S13: (Sample 9) Generated zero-shot Chain-of-Thought from InstructGPT `text-davinci-002` for three CoT prompts on a sample for the MedQA-USMLE test set.

USMLE [test:286] A 27-year-old HIV positive female gave birth to a 7lb 2oz (3.2 kg) baby girl. The obstetrician is worried that the child may have been infected due to the mother's haphazard use of her anti-retroviral medications. Which of the following assays would a hospital use detect the genetic material of HIV if the child had been infected?
A) Enzyme-linked immunosorbent assay (ELISA) B) Rapid HIV antibody test **C) Polymerase chain reaction** D) Southern blot

✓ Let's use step by step inductive reasoning, given the medical nature of the question.

The first step is to understand the question. In this case, the question is asking which of the following assays would a hospital use to detect the genetic material of HIV if the child had been infected.

The second step is to understand the options. In this case, the options are enzyme-linked immunosorbent assay (ELISA), rapid HIV antibody test, polymerase chain reaction, and Southern blot.

The third step is to understand what each option is. Enzyme-linked immunosorbent assay (ELISA) is a test that is used to detect antibodies in the blood. Rapid HIV antibody test is a test that is used to detect HIV antibodies in the blood. Polymerase chain reaction is a test that is used to amplify DNA. Southern blot is a test that is used to detect DNA.

The fourth step is to understand what the question is asking. In this case, the question is asking which of the following assays would a hospital use to detect the genetic material of HIV if the child had been infected.

The fifth step is to understand what each option would do. Enzyme-linked immunosorbent assay (ELISA) would not detect the genetic material of HIV if the child had been infected. Rapid HIV antibody test would not detect the genetic material of HIV if the child had been infected. Polymerase chain reaction would detect the genetic material of HIV if the child had been infected. Southern blot would detect the genetic material of HIV if the child had been infected.

The sixth and final step is to choose the best option. In this case, the best option is polymerase chain reaction.

Therefore, among A through D, the answer is C.

✓ Let's differentiate using step by step reasoning like a medical expert.

The first step is to understand what the question is asking. In this case, the question is asking which assay would a hospital use to detect the genetic material of HIV if the child had been infected.

The second step is to understand what each option is.

Option A is the ELISA test, which is a test that looks for antibodies in the blood.

Option B is the Rapid HIV antibody test, which is a test that looks for antibodies in the blood and can give results in as little as 20 minutes.

Option C is the Polymerase Chain Reaction test, which is a test that looks for the genetic material of the virus in the blood.

Option D is the Southern blot test, which is a test that looks for the genetic material of the virus in the blood and can be used to confirm the results of the PCR test.

The third step is to understand what we are looking for. In this case, we are looking for the genetic material of HIV.

The fourth step is to eliminate the options that are not related to what we are looking for. In this case, we are looking for the genetic material of HIV, so we can eliminate options A and B because they are both tests that look for antibodies in the blood.

This leaves us with options C and D. The fifth step is to eliminate the option that is not the best answer. In this case, option C is the best answer because it is a test that looks for the genetic material of the virus in the blood.

Therefore, among A through D, the answer is C.

✓ Let's derive the differential diagnosis step by step.

The first step is to consider if the child was infected in utero, during delivery, or postnatally. If the child was infected in utero or during delivery, then the child's blood would test positive for HIV RNA. If the child was infected postnatally, then the child's blood would test positive for HIV antibodies.

The second step is to consider which test would be most appropriate to confirm the diagnosis. ELISA and rapid HIV antibody tests are used to detect HIV antibodies. Polymerase chain reaction is used to detect HIV RNA. Southern blot is used to detect HIV DNA.

In this case, the most appropriate test to confirm the diagnosis would be polymerase chain reaction, since the child was likely infected in utero or during delivery.

Therefore, among A through D, the answer is C.

Pattern A	Correct reasoning step	Pattern D	Incorrect reasoning step
Pattern B	Correct recall of knowledge	Pattern E	Incorrect or insufficient knowledge
Pattern C	Correct reading comprehension	Pattern F	Incorrect reading comprehension

Figure S14: (Sample 10) Generated zero-shot Chain-of-Thought from InstructGPT `text-davinci-002` for three CoT prompts on a sample for the MedQA-USMLE test set.

USMLE [test:714] A 28-year-old male presents to the emergency department with chest pain. He reports that one hour ago he was climbing the stairs in his home when he suddenly developed excruciating chest pain. He describes the pain as ripping and radiating to the back. The patient's past medical history is significant for a heart murmur that is being followed by his outpatient cardiologist. He takes no medications. The patient works as a carpenter and reports being monogamous with his wife. He denies any history of intravenous drug use or recent travel. In the emergency department, the patient's temperature is 99.4°F (37.4°C), blood pressure is 94/56 mmHg, pulse is 121/min, and respirations are 14/min. On physical exam, the patient appears to be in moderate distress and is clutching his chest. He is tall and has joint hypermobility. His breath sounds are equal and full bilaterally and has weak distal pulses.

Which of the following is most likely to be found in this patient?

A) Holosystolic, high-pitched blowing murmur at the lower left sternal border **B) Late systolic crescendo murmur at the apex with mid-systolic click** C) Mid-diastolic murmur at the left lower sternal border D) Systolic ejection murmur at the right second intercostal space

✗ **Let's think step by step.** The patient has chest pain, so we're thinking about the heart. The patient has a heart murmur, so we're thinking about valvular disease. The patient is tall and has joint hypermobility, so we're thinking about Marfan syndrome. The patient has weak distal pulses, so we're thinking about aortic dissection.

The most likely diagnosis is aortic dissection. Aortic dissection is a tear in the aorta that can cause chest pain. Aortic dissection can be caused by Marfan syndrome, which is a condition that affects the connective tissue. Marfan syndrome can cause joint hypermobility and aortic aneurysms.

Therefore, among A through D, the answer is D, systolic ejection murmur at the right second intercostal space.

✗ **Let's think step by step like a medical expert.**

The first thing to consider is the patient's symptoms. The patient has chest pain that is described as "ripping" and radiating to the back.This suggests that the pain is originating from the heart.

The second thing to consider is the patient's past medical history. The patient has a heart murmur that is being followed by an outpatient cardiologist. This suggests that the patient may have a heart condition that is causing the chest pain.

The third thing to consider is the patient's medications. The patient is not taking any medications. This suggests that the chest pain is not being caused by a medication side effect.

The fourth thing to consider is the patient's occupation. The patient is a carpenter. This suggests that the chest pain may be caused by physical activity.

The fifth thing to consider is the patient's travel history. The patient denies any recent travel. This suggests that the chest pain is not being caused by travel.

The sixth thing to consider is the patient's intravenous drug use history. The patient denies any history of intravenous drug use. This suggests that the chest pain is not being caused by drug use.

The seventh thing to consider is the patient's physical examination. The patient appears to be in moderate distress and is clutching his chest. He has weak distal pulses. This suggests that the chest pain is causing the patient to feel distressed and that the patient's heart may not be pumping blood effectively.

Based on the above information, the most likely cause of the patient's chest pain is a systolic ejection murmur at the right second intercostal space.

Therefore, among A through D, the answer is D.

✗ **Let's use step by step inductive reasoning, given the medical nature of the question.** The first thing to note is that the patient has a heart murmur. This is significant because it means that the patient is at risk for developing heart problems.

The next thing to note is that the patient is experiencing chest pain. This is significant because it is a symptom of a heart attack.

The third thing to note is that the patient is tall and has joint hypermobility. This is significant because it means that the patient is at risk for developing aortic dissection.

Based on the information given, the most likely diagnosis is aortic dissection.

Therefore, among A through D, the answer is D.

Pattern A	Correct reasoning step	Pattern D	Incorrect reasoning step
Pattern B	Correct recall of knowledge	Pattern E	Incorrect or insufficient knowledge
Pattern C	Correct reading comprehension	Pattern F	Incorrect reading comprehension

References

S1 Robinson, J., Rytting, C.M., Wingate, D.. Leveraging Large Language Models for Multiple Choice Question Answering. Preprint at arXiv 2022;https://doi.org/10.48550/arXiv.2210.12353.

S2 Nori, H., King, N., McKinney, S.M., Carignan, D., Horvitz, E.. Capabilities of GPT-4 on Medical Challenge Problems. Preprint at arXiv 2023;https://doi.org/10.48550/arXiv.2303.13375.

S3 Black, S., Biderman, S., Hallahan, E., Anthony, Q., Gao, L., Golding, L., et al. Gpt-neox-20b: An open-source autoregressive language model. Preprint at arXiv 2022;https://doi.org/10.48550/arXiv.2204.06745.

S4 Team, M.N.. Introducing mpt-30b: Raising the bar for open-source foundation models. www.mosaicml.com/blog/mpt-30b; 2023. Accessed: 2023-06-22.

S5 Almazrouei, E., Alobeidli, H., Alshamsi, A., Cappelli, A., Cojocaru, R., Debbah, M., et al. Falcon-40B: an open large language model with state-of-the-art performance. https://falconllm.tii.ae; 2023.

S6 Dettmers, T., Pagnoni, A., Holtzman, A., Zettlemoyer, L.. QLoRA: Efficient Finetuning of Quantized LLMs. Preprint at arXiv 2023;https://doi.org/10.48550/arXiv.2305.14314.

S7 Zheng, L., Chiang, W., Sheng, Y., Zhuang, S., Wu, Z., Zhuang, Y., et al. Judging LLM-as-a-judge with MT-bench and Chatbot Arena. Preprint at arXiv 2023;https://doi.org/10.48550/arXiv.2306.05685.

S8 Touvron, H., Martin, L., Stone, K., Albert, P., Almahairi, A., Babaei, Y., et al. Llama 2: Open Foundation and Fine-tuned Chat Models. Preprint at arXiv 2023;https://doi.org/10.48550/arXiv.2307.09288.

S9 Chung, H.W., Hou, L., Longpre, S., Zoph, B., Tay, Y., Fedus, W., et al. Scaling Instruction-finetuned Language Models. Preprint at arXiv 2022;https://doi.org/10.48550/arXiv.2210.11416.

S10 Singhal, K., Tu, T., Gottweis, J., Sayres, R., Wulczyn, E., Hou, L., et al. Towards Expert-level Medical Question Answering with Large Language Models. Preprint at arXiv 2023;https://doi.org/10.48550/arXiv.2305.09617.

S11 Gu, Y., Tinn, R., Cheng, H., Lucas, M., Usuyama, N., Liu, X., et al. Domain-specific language model pretraining for biomedical natural language processing. ACM Transactions on Computing for Healthcare (HEALTH) 2021;3(1):1–23.

S12 Pal, A., Umapathi, L.K., Sankarasubbu, M.. Medmcqa: A large-scale multi-subject multi-choice dataset for medical domain question answering. PMLR 2022;174:248–260.

S13 Yasunaga, M., Leskovec, J., Liang, P.. LinkBERT: Pretraining Language Models with Document Links. Preprint at arXiv 2022;https://doi.org/10.48550/arXiv.2203.15827.

S14 Luo, R., Sun, L., Xia, Y., Qin, T., Zhang, S., Poon, H., et al. BioGPT: Generative Pre-trained Transformer for Biomedical Text Generation and Mining. Preprint at arXiv 2022;https://doi.org/10.48550/arXiv.2210.10341.

S15 Venigalla, A., Frankle, J., Carbin, M.. PubMed GPT: A domain-specific large language model for biomedical text. https://hai.stanford.edu/news/stanford-crfm-introduces-pubmedgpt-27b; 2022. Accessed: 2022-12-16.

S16 Taylor, R., Kardas, M., Cucurull, G., Scialom, T., Hartshorn, A., Saravia, E., et al. Galactica: A Large Language Model for Science. Preprint at arXiv 2022;https://doi.org/10.48550/arXiv.2211.09085.

S17 Jin, Q., Dhingra, B., Liu, Z., Cohen, W.W., Lu, X.. PubMedQA: A Dataset for Biomedical Research Question Answering. Preprint at arXiv 2019;https://doi.org/10.48550/arXiv.1909.06146.

S18 Hendrycks, D., Burns, C., Basart, S., Zou, A., Mazeika, M., Song, D., et al. Measuring Massive Multitask Language Understanding. Preprint at arXiv 2020;https://doi.org/10.48550/arXiv.2009.03300.

S19 Wang, X., Wei, J., Schuurmans, D., Le, Q.V., Chi, E.H., Zhou, D.. Self-consistency Improves Chain of Thought Reasoning in Language Models. Preprint at arXiv 2022;https://doi.org/10.48550/arXiv.2203.11171.

S20 Jin, D., Pan, E., Oufattole, N., Weng, W.H., Fang, H., Szolovits, P.. What disease does this patient have? a large-scale open domain question answering dataset from medical exams. Applied Sciences 2021;11(14):6421.
