# Supplementary material for: Can large language models reason about medical questions?
Source: Patterns (N Y). 2024 Mar 1;5(3):100943. doi: 10.1016/j.patter.2024.100943 (PMC10935498; doi:10.1016/j.patter.2024.100943)
Supplement: Document S2. Article plus supplemental information [file mmc2.pdf]

Article
Patterns

Can large language models reason about medical questions?

Graphical abstract

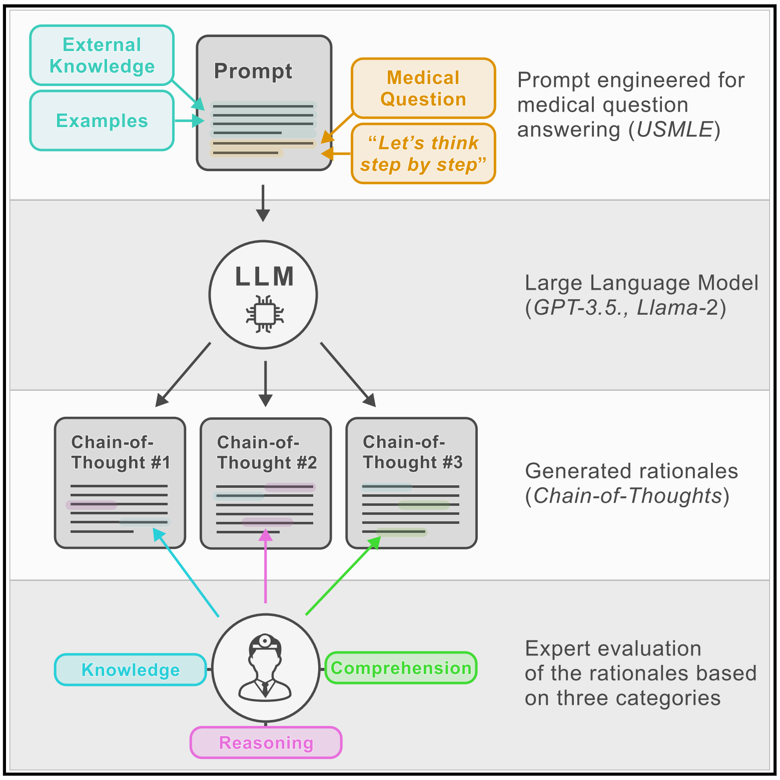

Highlights

- InstructGPT can mobilize expert medical knowledge and reasoning skills
- With adequate prompting, both closed- and open-source models pass the USMLE (MedQA)
- LLMs can quantify uncertainty when applied to medical exam multiple-choice questions
- LLMs are affected by the order of the answer options (positional bias)

Authors

Valentin Liévin,
Christoffer Egeberg Hother,
Andreas Geert Motzfeldt, Ole Winther

Correspondence

valentin.lievin@gmail.com

In brief

Large language models (LLMs) are powerful but their limits are unknown. This paper probes the capabilities of LLMs using medical exam questions (e.g., USMLE). Based on detailed answers generated by LLMs, we show that LLMs can often answer challenging medical questions by mobilizing expert knowledge and advanced reasoning capabilities. Both closed- and open-source LLMs can pass the USMLE (>60% accuracy). Our results support that future LLMs might be applicable to critical real-world applications such as supporting healthcare professionals.

Liévin et al., 2024, Patterns 5, 100943
March 8, 2024 © 2024 The Authors.
https://doi.org/10.1016/j.patter.2024.100943
CellPress

Patterns

CellPress
OPEN ACCESS

Article

Can large language models reason about medical questions?

Valentin Liévin,[1,2,6,*] Christoffer Egeberg Hother,[3] Andreas Geert Motzfeldt,[1] and Ole Winther[1,2,4,5]
[1]Section for Cognitive Systems, Technical University of Denmark, Anker Engelunds Vej 101, 2800 Kongens Lyngby, Denmark
[2]FindZebra, Rådvadsvej 36, 2400 Copenhagen, Denmark
[3]Department of Clinical Immunology, Copenhagen University Hospital, Rigshospitalet, Inge Lehmanns Vej 107, 2100 Copenhagen, Denmark
[4]Center for Genomic Medicine, Copenhagen University Hospital, Rigshospitalet, Ørestads Boulevard 5, 2300 Copenhagen, Denmark
[5]Bioinformatics Centre, Department of Biology, University of Copenhagen, Ole Maaløes Vej 5, 2200 Copenhagen, Denmark
[6]Lead contact
*Correspondence: valentin.lievin@gmail.com
https://doi.org/10.1016/j.patter.2024.100943

THE BIGGER PICTURE Foundation models have changed the way machine learning is practiced. Foundation models applied to text, so-called large language models (LLMs), have proven to be a disruptive technology. They might radically change the way we interact with computers.
In early 2022, it was clear that generalist LLMs can outperform domain-specific approaches in many domains. Benchmarks that reflect real-world scenarios were still needed, and today, it remains unclear how to best use and evaluate these models. This paper probes the capabilities of LLMs using medical exam questions (e.g., USMLE). Based on detailed step-by-step answers generated by LLMs, we show that LLMs can often answer challenging medical questions by mobilizing expert knowledge and advanced reasoning capabilities. Our results support that future LLMs might be applicable to critical real-world applications such as supporting healthcare professionals.

SUMMARY

Although large language models often produce impressive outputs, it remains unclear how they perform in real-world scenarios requiring strong reasoning skills and expert domain knowledge. We set out to investigate whether closed- and open-source models (GPT-3.5, Llama 2, etc.) can be applied to answer and reason about difficult real-world-based questions. We focus on three popular medical benchmarks (MedQA-US Medical Licensing Examination [USMLE], MedMCQA, and PubMedQA) and multiple prompting scenarios: chain of thought (CoT; think step by step), few shot, and retrieval augmentation. Based on an expert annotation of the generated CoTs, we found that InstructGPT can often read, reason, and recall expert knowledge. Last, by leveraging advances in prompt engineering (few-shot and ensemble methods), we demonstrated that GPT-3.5 not only yields calibrated predictive distributions but also reaches the passing score on three datasets: MedQA-USMLE (60.2%), MedMCQA (62.7%), and PubMedQA (78.2%). Open-source models are closing the gap: Llama 2 70B also passed the MedQA-USMLE with 62.5% accuracy.

INTRODUCTION

Self-supervised pre-training promises to turn vast quantity of raw data (e.g., text, images, audio) into general-purpose models. Language representations have transformed the field of natural language processing from simple word vectors to deep contextualized representations,[1–6] and language models are now ubiquitous in natural language processing. Notably, this ubiquity is thanks to the Transformer architecture and its compatibility with massively parallel computation hardware.[4]

Large language models (LLMs)

In recent years, tremendous resources have been allocated to scale Transformer-based language models to using hundreds of billions of parameters and to training on gigabytes of text.[7–16] This has so far translated to sustained gains[17] and enabled new ways to interact with language models. This progress made many of the past benchmarks obsolete and sparked a general interest for designing difficult enough benchmarks (e.g., BIG-bench).[18] Pre-train, prompt, and predict[19] is an emerging paradigm for applying LLMs to new problems without fine-tuning

This is an open access article under the CC BY license (http://creativecommons.org/licenses/by/4.0/).

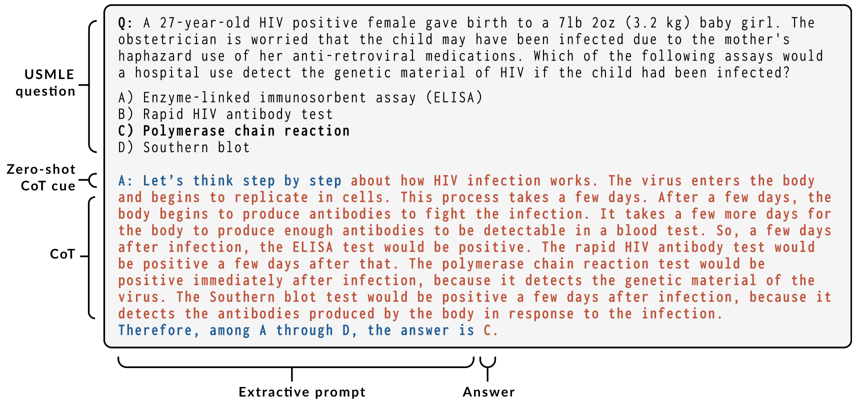

Figure 1. Answering a USMLE (US Medical Licensing Examination) question using zero-shot CoT prompting "Let's think step by step" and InstructGPT
Selected example.

the weights on the task. Prompt-based learning consists of augmenting the problem with instructions such that the model's completion of the prompt will correspond to a solution. This allows for LLMs to learn from a few examples (coined shots), which are simply incorporated into the prompts.[7]

Chain-of-thought (CoT) prompting

Initially, scaling language models up appeared to benefit more knowledge-intensive tasks than the reasoning-heavy ones.[8] Nevertheless, it was demonstrated that LLMs could be applied to system 2 problems by prompting the model to generate step-by-step solutions, coined CoT.[20] CoT prompting led to substantial improvements on many reasoning-intensive tasks,[20–23] allowing us to bridge the gap with human-level performances for most of the hard BIG-bench tasks.[24] As an alternative to writing reference step-by-step solutions, zero-shot CoT allows for generating CoTs using a single and domain-agnostic cue: "Let's think step by step"[25] (see example generated by InstructGPT[26] in Figure 1). The CoTs that result from that prompt not only appear to expose valid reasoning but also translate into superior zero-shot performances (see example in Figure 1).

LLMs and medical applications

Applying LLMs to real-life scenarios will require implementing additional safeguards. Language models may amplify the social biases present in the training data, may hallucinate incorrect facts, and may lack robustness,[27] for instance to adversarial attacks.[28] Therefore, deploying LLMs into sensitive areas such as healthcare must be operated with great care.[29,30] Nonetheless, LLMs are powerful tools and therefore have the potential to transform the field of machine intelligence. At the dawn of this research work, although LLMs had been tested on large benchmarks (MMLU,[31] BIG-bench[18]), studies applied to the medical domain were still needed. Specialized datasets such as the MedQA-US Medical Licensing Examination (USMLE) enable assessing the capabilities of LLMs in realistic clinical scenarios requiring specialized medical knowledge, advanced reasoning capabilities, and human-level reading comprehension skills.[32]

Related work

This article—written in three stages (v.1: July 2022, v.2: December 2022, and v.3: September 2023)—evolved along with the remaining of the field. December 2022 was a turning point in machine learning history; new records were achieved on medical benchmarks by the domain-specific Med-PaLM,[33,34] ChatGPT, and GPT-4.[35] ChatGPT sparked the interest of the public and the research community, which hastened to benchmark it against USMLE questions,[36,37] turning to self-curated data instead of the peer-reviewed MedQA benchmark. Involving human experts to evaluate the generated explanations on USMLE questions has also been explored in concurrent work.[33,37] Throughout the development of this research, significant progress happened in the open-source world (Llama 2[38]), and recently, there has been an investigation on both generalist and fine-tuned open-source LLMs applied to medical benchmarks.[39] CoT prompting and ensemble methods are now commonplace in the literature, whereas retrieval augmentation (grounding) remains less common.[33–35,39–41]

	Direct zero-shot	Zero-shot CoT
Question	Question: [Question]	Question: [Question]
CoT	∅	Answer: Let's think step by step <CoT>
Answer	Answer: among A through D, the answer is <answer>	Therefore, among A through D, the answer is <answer>
	**Direct zero-shot + grounding**	**One-shot CoT**
Shot	∅<br>∅<br>∅	Question: [Question]<br>Answer: Let's think step by step [Explanation]<br>Therefore, among A through D, the answer is [answer]
Context	Context: [Context]	∅
Question	Question: [Question]	Question: [Question]
CoT	∅	Answer: Let's think step by step <CoT>
Answer	Answer: among A through D, the answer is <answer>	Therefore, among A through D, the answer is* <answer>

Figure 2. Prompt templates
In the table, we use typewriter style and brackets to represent [provided data] such as the question, additional context, or the answer and <completions> generated by GPT-3. The symbol ∅ represents an empty string.

CellPress
OPEN ACCESS

Table 1. Answering accuracy of leading models against human performance on USMLE (test), MedMCQA (validation), and PubMedQA (test) datasets

Model	Date	USMLE	MedMCQA	PubMedQA
Codex 5-shot CoT[a]	2022	60.2	59.7	78.2
Llama 2 5-shot CoT[a]	2023	62.5	53.6	–
Fine-tuned SOTA	2022	50.3	52.9	78.2
GPT-4	2023	86.1	**73.7**	**81.2**
MedPalm v.2	2023	**86.5**	72.3	77.4
Human (passing score)	–	60.0	50.0	–
Human (expert score)	–	87.0	90.0	78.0

Find an overview of our results in supplemental information section A.
[a]Our best methods.

Contributions

This paper investigates the performances, interpretability, and limitations of CoT prompting for medical question answering. We utilized the GPT-3.5 series (InstructGPT and Codex). This research was conducted in three rounds; first, using InstructGPT, we investigated variations of zero-shot CoT prompting for medical reasoning (domain-specific CoT cues, retrieval augmentation), looking both at the answering performances and the limitations based on an expert evaluation. In the second round, thanks to the Codex beta program, we investigated how scaling inference-time compute could be applied to challenge both the human baseline and to quantify uncertainty. Last, we benchmarked a range of open-source models. Our contributions are as follows.

- We assess how GPT-3.5 performs on multiple-choice medical board exam question datasets (MedQA-USMLE and MedMCQA) and a medical reading comprehension dataset (PubMedQA) using prompt engineering. We explore zero-/few-shot, direct/CoT, domain-specific CoT cues and retrieval augmentation.
- We propose an evaluation protocol for evaluating generated CoTs (three main categories: reasoning, knowledge, and reading comprehension). A medical expert annotated a subset of CoTs generated by zero-shot InstructGPT and supports that InstructGPT, in many cases, can reason and exploit memorized expert knowledge.
- We demonstrate that scaling inference-time compute enables Codex 5-shot CoT to be well calibrated and to reach passing scores on the three medical datasets.
- We benchmark open-source LLMs on the MedQA-USMLE and MedMCQA.

Development

This article has evolved over three distinct versions, each exploring different facets of LLMs:

- v.1, July 2022: investigated InstructGPT (expert evaluation and benchmarking prompting strategies).
- v.2, December 2022: scaled experiments and passed the MedQA-USMLE using Codex.
- v.3, September 2023: evaluated open-source models Llama 2, Vicuna, Guanaco, Falcon, etc.

METHOD

This paper explores variations of prompt engineering for medical question answering. The prompt templates are summarized in Figure 2.

Zero shot

We studied two classes of prompts: the direct prompt and zero-shot CoT. The direct prompt triggers the model to generate the answer using a single completion step (i.e., ''The answer is''), whereas when applying the zero-shot CoT framework, we use a two-step prompting scheme: first, an initial reasoning prompt with a CoT cue (e.g., ''Let's think step by step''), the completion of which is the CoT, and second, an extractive prompt, the completion of which is the answer (e.g., ''Therefore the answer is''). In the zero-shot CoT setting, this corresponds to the setup described in Kojima et al.,[25] and the direct setting corresponds to Brown et al.[7]

Few-shot

We experimented with inserting examplars (or shots) of question-answer pairs and question-explanation-answers triplets in the prompts. We built each shot using the zero-shot template, replacing the output with the reference explanations and answers. In the few-shot CoT setting, our setup matches the one from Wei et al.[20]

Table 2. Summary of the medical question answering datasets

	MedQA-USMLE[32]	MedMCQA[44]	PubMedQA[45]
Answer options	A/B/C/D	A/B/C/D	yes/no/maybe
Questions (train/valid./test)	10,200/1,300/1,300	182,800/4,200/6,100	450/50/500
Words/question	116.6	12.7	253.3
Source (questions)	national medical board examination (US)	AIIMS and NEET PG entrance exams	expert-annotated PubMed abstracts
Words/explanation	41.6	66.2	43.2
Source (explanations)	5 human-written CoTs (sourced from MMLU[46])	detailed explanations (original dataset)	long answer (original dataset)

valid., validation.

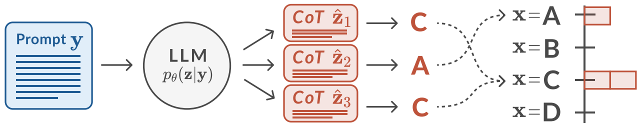

Figure 3. Generative process and answer likelihood (ensemble model, i.e., self-consistency)

Answer likelihood

We denote $\mathbf{x}$ the answer string, $\mathbf{y}$ a prompt, and $\mathbf{z}$ a completion generated from an LLM denoted p_θ. In the zero-shot setting, sampling $\hat{\mathbf{z}} \sim p_\theta(\mathbf{z}|\mathbf{y})$ is a two-step process (first generate the CoT, then extract the answer) pictured in Table 2. Using a sampling temperature τ, k completions $\hat{\mathbf{z}}_1, \ldots, \hat{\mathbf{z}}_k$ can be sampled from the generative LLMs. We aggregate the completions and estimate the marginal answer likelihood as (Figure 3)[42]:

$$p_\theta(\mathbf{x}|\mathbf{y}) \approx \frac{1}{k}\sum_{i=1}^{k} 1[\mathbf{x} \in \hat{\mathbf{z}}_i], \quad \hat{\mathbf{z}}_1, \ldots, \hat{\mathbf{z}}_k \sim p_\theta(\mathbf{z}|\mathbf{y}) \qquad \text{(Equation 1)}$$

where $1[\mathbf{x} \in \hat{\mathbf{z}}_i]$ takes value one when the answer $\mathbf{x}$ can be matched in the completion $\hat{\mathbf{z}}$ and otherwise takes zero. Sampling multiple completions may allow exploring multiple hypotheses. Combining multiple sampled CoTs (also known as self-consistency) has also been explored in past work, and showed improvements over single-sample CoT methods.[42,43]

Retrieval augmentation

LLMs memorize part of the knowledge embedded into the training data; nonetheless, models might fail to reuse this knowledge effectively during prediction. Conditioning the predictions on a knowledge base is an alternative research direction for improving language models.[47–49]

We investigated whether grounding the model with additional context could improve the answering accuracy. We experimented with a simple BM25 retriever and used Wikipedia as a knowledge base. Read more details in supplemental information section G.

EXPERIMENTS

This section is separated into three parts: (1) introducing the datasets and the GPT-3.5 models, (2) investigating zero-shot medical reasoning with InstructGPT, and (3) scaling inference-time compute with Codex (using longer few-shot prompts and sampling many completions per question).

Resources availability

Lead contact

Further information and requests for code and data should be directed to and will be fulfilled by the lead contact, Valentin Liévin (valentin.lievin@gmail.com).

Table 3. Selected domain-specific CoT cues

#1 – *Let's think step by step*
#2 – *Let's think step by step like a medical expert*
#3 – *Let's use step by step inductive reasoning, given the medical nature of the question*
#4 – *Let's differentiate using step by step reasoning like a medical expert*
#5 – *Let's derive the differential diagnosis*

Materials availability

This study did not generate new unique materials or reagents.

Data and code availability

Our source code is available on Github (https://github.com/vlievin/medical-reasoning).[50] A collection of generated CoTs, reusable for downstream tasks, are accessible through ToughtSource.[51] All our benchmark results are summarized in supplemental information section A and Table S2.

Datasets and models

Datasets

This study is centered around three medical multiple-choice question answering datasets: USMLE, which includes difficult real-world medical questions targeting medical professionals[32]; the MedMCQA, which gathers questions from medical school entrance exams[44]; and the PubMedQA, which includes reading comprehension questions about PubMed abstracts.[45] The three datasets are summarized in Table 2. For each dataset, we gathered questions with explanations (long answer), which we used as reference CoTs in few-shot learning scenarios. We present the three datasets in further details in supplemental information section C. Furthermore, we compare the MedQA-USMLE with the MMLU-USMLE dataset in supplemental information section D; we found the MedQA questions to be more challenging than the MMLU ones.[31]

Models

We study a collection of closed- and open-source models. The 175-billion parameter GPT-3.5 series: the human-aligned GPT-3 (InstructGPT, text-davinci-002[26]) and the code-fine-tuned GPT-3 (Codex, code-davinci-002).[52] A collection of open-source models ranging from 7 to 70 billion parameters: Llama 2,[38] Vicuna,[53] Guanaco,[54] Falcon,[55] MPT,[56] and GPT-NeoX.[57] We used greedy decoding (temperature $\tau = 0$) with $k = 1$ sample unless specified otherwise (e.g., ensemble methods).

In supplemental information section E, we report the test USMLE accuracy for four GPT-3 versions: a small GPT-3, the largest GPT-3 trained without human alignment, InstructGPT, and Codex. The smaller model text-curie-002 delivered close to random performances, with a maximum accuracy of 27.9%. The non-aligned largest GPT-3 text-davinci-001 scored 40.2%, whereas the largest code pre-trained Codex scored 52.9%, and the code-pre-trained and human-aligned InstructGPT scored 47.1%.

Investigating zero-shot reasoning with InstructGPT

In this section, we investigate whether the good generative capabilities of LLMs can be applied to answer medical questions in a zero-shot setting. We investigate variations of the zero-shot CoT framework: using domain-specific CoT cues and augmenting the prompt with Wikipedia passages.

In addition to the original zero-shot CoT cue "Let's think step by step," we tested 29 other domain-specific variations such as "Let's think step by step like a medical expert." The study is available in supplemental information section B. We selected five CoT cues displayed in Table 3. In supplemental information section I, we display CoT samples for more exotic cues such as "Let's follow a Bayesian step by step approach," "Let's work by elimination," and "Let's reflect on each answer option."

Zero-shot benchmark

In Table 4, we report the performances of InstructGPT for the direct prompt and the aggregated performances for the five domain-specific CoT cues (Table 3). We explored augmenting the prompts with retrieved Wikipedia passages (grounding) and report the performances of an ensemble model with majority voting.[42]

Zero-shot direct

InstructGPT outperformed the domain-specific and fine-tuned BERT baselines on the three datasets. Without BM25 grounding, InstructGPT scored +1.4% on the USMLE questions, +1.0% on the MedMCQA exam questions, and +1.1% on PubMedQA over the best BERT methods.

Zero-shot CoT

Without BM25 grounding, the direct prompt remained, on average, a better alternative to the CoT prompts. Performances were lower for each of the considered CoT cues, except in the case of the USMLE dataset, for which half of the CoT prompts resulted in small improvements over the direct prompt (+1.1% using CoT prompt #1 vs. using the direct prompt). Nonetheless, the

Table 4. Zero-shot answering accuracy of InstructGPT (text-davinci-002) on the MedQA-USMLE (test), MedMCQA (valid), and PubMedQA (test) datasets

Model	Grounding	Prompt	USMLE	MedMCQA	PubMedQA
InstructGPT	∅	direct	46.0	44.0	**73.2**
InstructGPT	∅	CoT #1–#5	46.1 ± 0.7	40.4 ± 2.2	59.9 ± 3.5
InstructGPT	BM25	direct	47.3	46.7	–
InstructGPT	BM25	CoT #1–#5	46.4 ± 0.7	42.5 ± 1.7	–
InstructGPT	∅	ensemble (n = 6)	50.0	42.4	70.4
InstructGPT	BM25	ensemble (n = 6)	49.3	**48.8**	–
InstructGPT	∅ + BM25	ensemble (n = 12)	**53.1**	47.6	–
Fine-tuned BERT	BM25, DPR, ∅	–	44.6	43.0	72.2
Human (passing score)	–	–	60.0	50.0	–
Human (expert score)	–	–	87.0	90.0	78.0

We report the best fine-tuned BERT-based methods. We tested 5 domain-specific CoT cues (#1–#5) and report the mean performances with standard deviations. Fine-tuned BERT, BioLinkBERT[58]; DPR, dense passage retrieval.[59] When multiple results are aggregated, we report the mean and standard deviation (±).

domain-specific CoT prompts #2–#5 did not significantly outperform the original CoT prompt #1.

Knowledge grounding

In an attempt to exploit the good reading comprehension skills of InstructGPT, we explored conditioning the completions on Wikipedia passages. When using the direct prompt, we recorded gains on the USMLE (+1.3%) and on the MedMCQA (+2.7%) datasets, suggesting that retrieval augmentation might be beneficial.

Ensemble

Combining the predictions of multiple prompts outperformed the single-prompt predictions, except in the case of the PubMedQA dataset, for which the direct prompt performed exceptionally well. The best performances on the USMLE and MedMCQA datasets were obtained by combining retrieval-augmented prompts and setting a maximum of 53.1% accuracy on the USMLE dataset and 48.8% validation accuracy on the MedMCQA dataset.

Expert evaluation of the generated CoTs

Protocol

InstructGPT delivered strong performances using zero-shot CoT prompting. In this section, we investigate whether the CoTs are sound and seek to understand better how the model fails and succeeds. We considered three general skills that we expect are required to be mastered to answer medical questions: (1) the ability to perform non-trivial reasoning steps, (2) the ability to recall knowledge that is not provided in the context, and (3) the ability to comprehend the question and the context. Based on the three skills, we defined three success patterns (A, B, C) and three failure patterns (D, E, F).

A subset of 50 CoTs generated based on USMLE questions were annotated by a medical expert (C.E.H.) using the six categories. For each category and each CoT, we reported a match if the pattern could be observed at least once. This means that a CoT can be labeled with both a correct and an incorrect pattern for the same skill. We showcase thirty annotated CoTs (three in Figure 9 and 27 in supplemental information section I).

Analysis

We report the frequencies of occurrence for the six patterns in Table 5. We found that most of the questions answered incorrectly triggered generating CoTs that contained reasoning errors (pattern D, 86%) and that exhibited a lack of knowledge (pattern E, 74%). Misunderstanding of the questions or the context was less frequently observed (pattern F, 50%). We observed that CoTs leading to questions answered correctly could still show failure patterns but we also observed that the CoTs leading to incorrect answers were not entirely incorrect, as 59% contained at least one correct reasoning step and 65% showed proper recall of knowledge. Furthermore, inspecting the CoTs leading to incorrect answers more closely, we found that 47% of those were inconclusive: the model could not narrow down the prediction to a single answer.

Answering bias

In Figure 4, we report the frequencies of the USMLE answers and the frequencies of predicted labels (zero-shot InstructGPT) for the direct and CoT prompts. Both prompting schemes led to biased predictive frequencies. Direct prompting led to overestimating labels C and D while underestimating label A. CoT prompting led to underestimating B and C while overestimating label D. We repeated the experiment using randomly permuted labels and observed similar patterns (see supplemental information section F).

Scaling inference-time compute with codex

In the second round of experiments, we investigated whether using more inference-time compute, thanks to the Codex beta program, could be utilized to obtain better performances and more interpretable outputs. Codex enables using longer prompts, so we used 5-shot prompts and experimented with sampling $k = 100$ completions with temperature $\tau = 0.5$ for each question.

Table 5. Frequency of observed patterns (A, B, C, D, E, F) identified among 50 CoTs generated by InstructGPT with temperature $\tau = 0$

	Pattern	Correct, % (16)	Incorrect, % (34)	Total, % (50)
**A**	correct reasoning step	94 (15)	59 (20)	**70** (35)
**B**	correct recall of knowledge	87 (14)	65 (22)	**72** (36)
**C**	correct reading comprehension	100 (16)	85 (29)	**90** (45)
**D**	incorrect reasoning step	12 (2)	86 (29)	**62** (31)
**E**	incorrect or insufficient knowledge	25 (4)	74 (25)	**58** (29)
**F**	Incorrect reading comprehension	6 (1)	50 (17)	**36** (18)

The CoTs are generated based on USMLE questions and using the CoT prompts #1–#5 (Table 3). We report the frequencies of CoTs leading to correct and incorrect predictions along with the total.

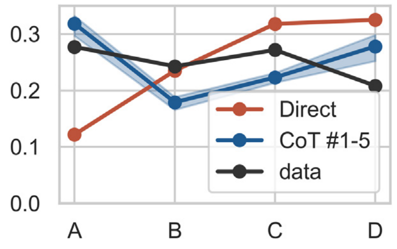

Figure 4. Frequencies of USMLE answers and InstructGPT (text-davinci-002) predictions for direct and CoT prompts (no grounding, zero-shot)

We report question-answering performances and results on uncertainty quantification.

Codex 5-shot CoT: Sampling and combining multiple CoTs

In Figure 5, we report the performances of Codex 5-shot CoT given subsets of $k' < k$ CoTs. We report the best fine-tuned models and the human baseline. Increasing the budget of samples yields better results.[42] Using an ensemble of the k samples, Codex 5-shot CoT reaches the passing score on the three tasks (see Table 1): the USMLE dataset (60.2% $\geq$ 60%), the MedMCQA dataset (62.7% $\geq$ 50%), and the PubMedQA dataset (78.2% $\geq$ 78%). Additional results, including performances in zero-shot settings, are available in Table S2 (supplemental information section A). Although Codex performed exceptionally well with 5 shots, Codex yield feeble performances with zero-shot CoT; inspecting the generated CoTs revealed lesser-quality samples (supplemental information section I).

Uncertainty quantification

We investigate the answering likelihood Equation 1 given by Codex 5-shot CoT with $k = 100$ samples. In Figure 6, we report the maximum probability assigned by the model for correctly vs. incorrectly answered questions along with the calibration plots for the three datasets. Codex 5-shot CoT appears to be overall calibrated, although the calibration is worse for the PubMedQA dataset.

Benchmarking open-source models

In the rapidly evolving landscape of LLMs, a prevalent question is the performance gap between open-source and closed-source models. Our study focused on the capabilities of InstructGPT and Codex. Given a budget of 2.000 GPU hours (NVIDIA A100), we benchmarked a range of open-source LLMs, with parameter sizes ranging from 7 to 70 billion, against the 175-billion-parameter Codex. In Figure 7, we report the predictive performances, calibration plots, and biases for Llama 2, Vicuna 1.5, and Codex using up to $k = 100$ CoT samples. We provided additional results in Figure 8 in supplemental information section H (zero- and 5-shot, MedQA-USMLE, and MedMCQA).

DISCUSSION

Zero-shot LLMs outperform fine-tuned BERT

Zero-shot InstructGPT and Codex outperformed fine-tuned BERT models on three challenging question-answering datasets (see zero-shot benchmark and supplemental information section A). In the case of the USMLE and the MedMCQA datasets, the retrieval-augmented BERT baselines were outperformed by several LLMs, regardless of augmenting the prompts with Wikipedia passages. This suggests that LLMs, without fine-tuning, can mobilize medical knowledge and problem-solving skills.

Zero-shot CoT prompting often yields sound and interpretable step-by-step solutions

For both InstructGPT and Codex, single-sample CoT prompting was not found to be competitive with direct prompting (see zero-shot benchmark and supplemental information section A). Nevertheless, CoTs are human readable and therefore interpretable. Our expert evaluation (see expert evaluation of the generated CoTs) revealed that CoTs are often sound: even though InstructGPT still does make mistakes, it was often able to reason, recall medical knowledge, and comprehend the given problem. In the section investigating zero-shot reasoning with InstructGPT and supplemental information section B, we explored domain-specific CoTs cues such as "Let's think step by step like a medical expert." Although such prompts, taken separately, did not outperform the original zero-shot CoT prompt (see Table S2 in supplemental information section A), more specific prompts appeared to trigger alternative strategies such as working by elimination or manipulating equations (see supplemental information sections B and I). Investigating whether a task-specific prompt could help solve specific tasks will be left for future research. A collection of generated CoT samples are presented in supplemental information section I, and many more samples are available on our GitHub page.

LLMs memorize some expert knowledge

The expert evaluation of the generated CoTs (see expert evaluation of the generated CoTs) and the good results obtained on the medical exam questions (see Table S2; supplemental information section A) suggest that GPT-3.5 memorizes domain knowledge. Nevertheless, despite the simplicity of the BM25 retriever and the small number of retrieved documents prepended in each prompt, grounding InstructGPT resulted in slight improvements (see Table 4). This suggests that InstructGPT is not omniscient, and so (1) using stronger retrievers such as commercial search engines or dense retrievers,[49] (2) using a more complete knowledge base,[48] or (3) leveraging inference-time compute by retrieving, reranking, and processing more passages[49] might improve performances.

Bias

In the section answering bias, we exposed the biases induced by the use of direct and CoT prompts. In the case of the direct

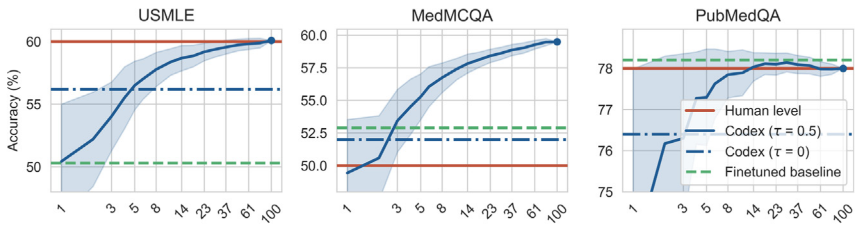

Figure 5. Sampling and combining multiple CoTs

Answering accuracy of Codex 5-shot CoT (code-davinci-002) on the USMLE (test), the MedMCQA (validatuin), and the PubMedQA (test) datasets for 100 CoTs sampled with temperature $\tau \in \{0, 0.5\}$. We report the average accuracy for ensemble models evaluated using random subsets of $k' = 1 \ldots 100$ CoTs. We report the mean and standard deviation. We display the performances of the best fine-tuned methods along with the lower human baselines.

CellPress
OPEN ACCESS

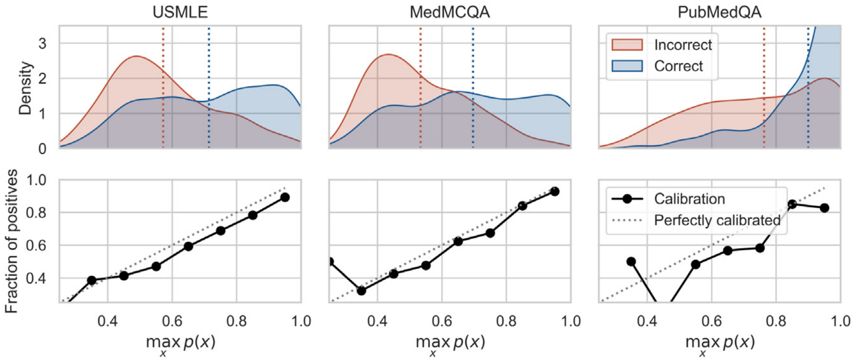

Figure 6. Uncertainty quantification

First row: distribution of the probability assigned to the correct label for correct predictions and incorrect predictions (see Equation 1). Second row: calibration plot. The probabilities are obtained using Codex 5-shot CoT and an ensemble of $k = 100$ predictions sampled with temperature $\tau = 0.5$.

prompt, answer D was most often selected, which might be due to its proximity to the generated answer. In the case of the CoT prompts, labels A and D were selected more often, which might be a result of often beginning CoTs with content related to option A. Based on an inspection of the CoTs, we speculate that GPT-3 defaults to this behavior when it cannot answer but still attempts to complete the prompt with a default answer (D or A). Shuffling the answer options might be one way to overcome this limitation; however, other forms of biases might still be present.

Generating and combining many CoTs bridges the gap with human-level performances

CoTs can be combined and/or filtered using human or automated feedback.[42,60] In the section scaling inference-time compute with Codex, we showed that sampling and combining up to $k = 100$ completions using Codex or Llama 2 with 5-shot CoT prompts was sufficient to pass both the MedMCQA and the challenging USMLE, although a large gap remains between our models and the human experts.

5-Shot CoT-prompted LLMs are close to well calibrated

In the sections uncertainty quantification and benchmarking open-source models, we looked at the probability assigned to correct and incorrect predictions using the ensemble model from Equation 1. We found Codex and Llama 2 to be close to well calibrated, corroborating the results that "language models (mostly) know what they know."[61]

Scale, code pre-training, human-alignment, and few-shot learning

In supplemental information section E, we compared multiple GPT-3 models in the zero-shot setting. Best performances are obtained using Codex, outperforming the human-aligned InstructGPT, which is a fine-tuned version of Codex. Human alignment might impair performances; Codex (without alignment) was not as robust as InstructGPT (with alignment) in zero-shot CoT setting (see performances in Table S2 in supplemental information section A and see CoT samples in supplemental information section I). Nevertheless, 5-shot prompting allowed us to bypass the zero-shot limitations of Codex. We observed a similar pattern when comparing the versions of LLama-2 70b: the base version outperformed the chat version (supplemental information section H). Instruction-fine-tuned models might lose in-context learning abilities.

Open-source models narrow the gap with proprietary counterparts

Open-source models, despite having fewer parameters, are approaching the performance of proprietary ones (Figures 7 and 8). For instance, Llama 2 outperforms Codex with just half the parameters.

Instruction-fine-tuned LLMs like Guanaco and Vicuna performed exceptionally well (Figure 8). Surprisingly, Vicuna 1.5 13B's superior performance to both Llama 2 versions underscores the significance of high-quality datasets for instruction-based fine-tuning.[62]

Conclusion

We applied zero-shot, few-shot direct, and CoT prompting to medical question answering with and without retrieval augmentation. Zero-shot InstructGPT significantly outperformed the fine-tuned BERT baselines. CoT prompting proved to be a powerful tool leading to better performances and more interpretable predictions. Our expert evaluation suggests that LLMs can mostly comprehend complex medical questions, can often recall expert-domain knowledge, and can often perform non-trivial reasoning steps.

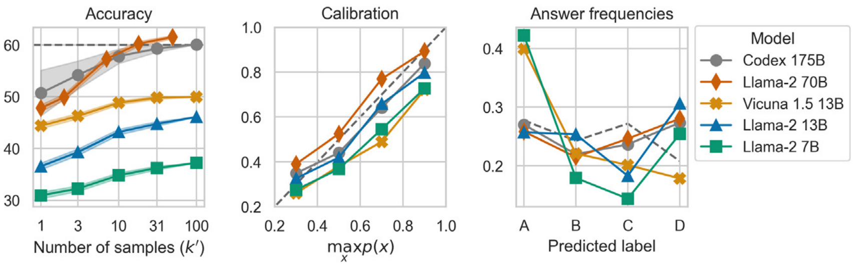

Figure 7. Comparing open-source LLMs against the closed-source Codex on the MedQA-USMLE benchmark ($\tau = 0.9$, up to $k = 100$ samples)

We report answering accuracy, model calibration, and answering bias.

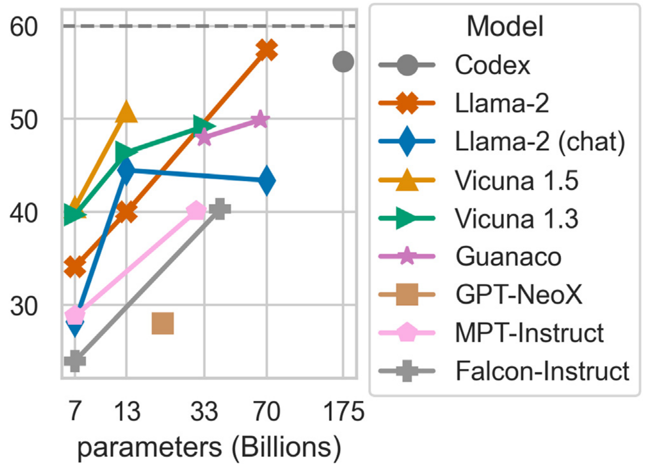

Figure 8. MedQA-USMLE accuracy vs. model size
All experiments were performed using a 5-shot CoT prompting strategy and greedy decoding ($\tau = 0$). Llama 2 70B outperforms Codex 175B (proprietary).

Although InstructGPT and Codex still make mistakes, we found that scaling inference-time compute by sampling many CoTs per question could overcome part of these limitations. With 100 samples, Codex 5-shot CoT delivered unprecedented performances on the three datasets, bridging the gap with human-level performances and virtually passing the USMLE by 0.2% points. Our exploration into open-source LLMs indicated their competitive stance in medical benchmarks. Llama 2 outperformed Codex by 2 points on the USMLE in spite of a much smaller parameter footprint.

However, deploying LLMs in real-life clinical scenarios will require the development of more robust techniques. We exposed one form of bias (ordering of the answer options affects the predictions), but many more might affect predictions, including those hidden in the training data (e.g., gender, race, …). Nevertheless, a lack of knowledge might be more easily compensated; our experiment with BM25, albeit limited, suggests that augmenting the prompt with factual data improves performances.

Since the completion of v.2 of this work, both GPT-4 and MedPalm 2 have achieved performance on USMLE around 85%.[35,63] This is not unexpected given the evolution the LLM field has witnessed recently. Although benchmark contamination in training sets for both proprietary and open-source LLMs is a valid concern, these results indicate that both open- and closed-source LLMs hold great potential for assisting human decision-making in medicine and beyond.

SUPPLEMENTAL INFORMATION

Supplemental information can be found online at https://doi.org/10.1016/j.patter.2024.100943.

ACKNOWLEDGMENTS

We thank OpenAI for granting access to the Codex beta program. We acknowledge the EuroHPC Joint Undertaking for awarding us access to MeluXina at LuxProvide, Luxembourg. V.L.'s work was funded in part by Google DeepMind through a PhD grant. O.W.'s work was funded in part by the Novo Nordisk Foundation through the Center for Basic Machine Learning Research in Life Science (NNF20OC0062606). V.L., A.G.M., and O.W. acknowledge support from the Pioneer Center for AI, DNRF grant number P1.

AUTHOR CONTRIBUTIONS

Conceptualization, V.L., C.E.H., and O.W.; methodology, V.L. and O.W.; software, V.L. and A.G.M.; investigation, V.L. and A.G.M.; writing – original draft, V.L.; writing – review & editing, all authors; data curation, C.E.H.; funding acquisition, O.W.; supervision, O.W. and V.L.

DECLARATION OF INTERESTS

The authors declare no competing interests.

DECLARATION OF GENERATIVE AI AND AI-ASSISTED TECHNOLOGIES IN THE WRITING PROCESS

The authors used GPT-3.5 and ChatGPT to help reformulate paragraphs in the writing process. After using generative technologies, the authors reviewed and edited the content as needed and take full responsibility for the content of the publication.

Received: December 29, 2022
Revised: March 18, 2023
Accepted: February 2, 2024
Published: March 1, 2024

REFERENCES

1. Mikolov, T., Sutskever, I., Chen, K., Corrado, G.S., and Dean, J. (2013). Distributed representations of words and phrases and their compositionality. Adv. Neural Inf. Process. Syst. *26*.
2. Pennington, J., Socher, R., and Manning, C. (2014). GloVe: Global vectors for word representation. In Proceedings of the 2014 Conference on Empirical Methods in Natural Language Processing (EMNLP) (Association for Computational Linguistics), pp. 1532–1543. https://doi.org/10.3115/v1/D14-1162.
3. Peters, M.E., Neumann, M., Iyyer, M., Gardner, M., Clark, C., Lee, K., and Zettlemoyer, L. (2018). Deep contextualized word representations. Preprint at arXiv. https://doi.org/10.48550/arXiv.1802.05365.
4. Vaswani, A., Shazeer, N., Parmar, N., Uszkoreit, J., Jones, L., Gomez, A.N., Kaiser, Ł., and Polosukhin, I. (2017). Attention is all you need. Adv. Neural Inf. Process. Syst. *30*.
5. Devlin, J., Chang, M., Lee, K., and Toutanova, K. (2018). BERT: Pre-training of Deep Bidirectional Transformers for Language Understanding. Preprint at arXiv. https://doi.org/10.04805/arXiv.1810.04805.
6. Radford, A., Narasimhan, K., Salimans, T., and Sutskever, I. (2018). Improving language understanding by generative pre-training. https://cdn.openai.com/research-covers/language-unsupervised/language_understanding_paper.pdf.
7. Brown, T., Mann, B., Ryder, N., Subbiah, M., Kaplan, J.D., Dhariwal, P., Neelakantan, A., Shyam, P., Sastry, G., Askell, A., Agarwal, S., et al. (2020). Language models are few-shot learners. Adv. Neural Inf. Process. Syst. *33*.
8. Rae, J.W., Borgeaud, S., Cai, T., Millican, K., Hoffmann, J., Song, H.F., Aslanides, J., Henderson, S., Ring, R., Young, S., Rutherford, E., et al. (2021). Scaling Language Models: Methods, Analysis & Insights from Training Gopher. Preprint at arXiv. https://doi.org/10.48550/arXiv.2112.11446.
9. Chowdhery, A., Narang, S., Devlin, J., Bosma, M., Mishra, G., Roberts, A., Barham, P., Chung, H.W., Sutton, C., Gehrmann, S., et al. (2023). Palm: Scaling language modeling with pathways. J. Mach. Learn. Res. *24*, 1–113.
10. Thoppilan, R., Freitas, D.D., Hall, J., Shazeer, N., Kulshreshtha, A., Cheng, H., Jin, A., Bos, T., Baker, L., Du, Y., et al. (2022). LaMDA: Language

USMLE [test:731] Three days after induction chemotherapy, a 9-year-old boy is admitted to the hospital for fever and neutropenia. He was initially diagnosed with t(9;22)-positive acute T cell lymphoblastic leukemia following workup for fatigue and pancytopenia. He is fully conscious. His blood pressure is 110/65 mm Hg, temperature is 39.5°C (103.1°F), pulse is 98/min, and respiratory rate is 14/min. Physical examination including examination of the central venous catheter site shows no source of infection. Broad-spectrum antibiotics are initiated. Which of the following factors about this patient most strongly indicates a poor prognosis?
A) Age **B) Cytogenetics** C) Fever and neutropenia D) Initial presentation

✓ **Let's use step by step inductive reasoning, given the medical nature of the question.**
The first thing that stands out is that the patient is 9 years old. In general, children have a better prognosis than adults when it comes to leukemia.
The next thing to consider is the patient's cytogenetics. The fact that the patient has t(9;22)-positive acute T cell lymphoblastic leukemia indicates that the patient has a specific chromosomal abnormality that is associated with a poor prognosis.
The next thing to consider is the patient's fever and neutropenia. Fever and neutropenia are both signs of infection, and infection is a serious complication in leukemia patients, particularly in those who are immunocompromised. Finally, we need to consider the patient's initial presentation. The fact that the patient was initially diagnosed with leukemia following workup for fatigue and pancytopenia indicates that the patient's leukemia was advanced at the time of diagnosis.
All of these factors indicate a poor prognosis for this patient.
Therefore, among A through D, the answer is B, cytogenetics.

✗ **Let's think step by step like a medical expert.** The first step is to gather information about the patient. In this case, we are given the following information: -The patient is 9 years old -The patient has t(9;22)-positive acute T cell lymphoblastic leukemia -The patient is currently experiencing fever and neutropenia
The next step is to analyze the information and look for any patterns or clues that could help us answer the question.
In this case, the most important factor that indicates a poor prognosis is the patient's age. Children under the age of 10 have a much higher chance of relapsing after treatment and a lower chance of achieving remission.
Therefore, among A through D, the answer is A.

✗ **Let's derive the differential diagnosis step by step**:
1) The patient has fever and neutropenia.
2) The patient is 9 years old.
3) The patient has t(9;22)-positive acute T cell lymphoblastic leukemia.
4) The patient's initial presentation was fatigue and pancytopenia.
The most likely cause of the patient's fever and neutropenia is infection. The patient's age, cytogenetics, and initial presentation are all risk factors for infection.
Therefore, the factor that most strongly indicates a poor prognosis is the patient's age.
Therefore, among A through D, the answer is A.

Pattern A	Correct reasoning step	Pattern D	Incorrect reasoning step
Pattern B	Correct recall of knowledge	Pattern E	Incorrect or insufficient knowledge
Pattern C	Correct reading comprehension	Pattern F	Incorrect reading comprehension

Figure 9. (Sample 1) Generated zero-shot CoT from InstructGPT text-davinci-002 for three CoT prompts on a sample for the MedQA-USMLE test set

Models for Dialog Applications. Preprint at arXiv. https://doi.org/10.48550/arXiv.2201.08239.

11. Hoffmann, J., Borgeaud, S., Mensch, A., Buchatskaya, E., Cai, T., Rutherford, E., Casas, D.D.L., Hendricks, L.A., Welbl, J., Clark, A., et al. (2022). Training Compute-optimal Large Language Models. Preprint at arXiv. https://doi.org/10.48550/arXiv.2203.15556.

12. Smith, S., Patwary, M., Norick, B., LeGresley, P., Rajbhandari, S., Casper, J., Liu, Z., Prabhumoye, S., Zerveas, G., Korthikanti, V., et al. (2022). Using DeepSpeed and Megatron to Train Megatron-turing NLG 530B, A Large-scale Generative Language Model. Preprint at arXiv. https://doi.org/10.48550/arXiv.2201.11990.

13. Zhang, S., Roller, S., Goyal, N., Artetxe, M., Chen, M., Chen, S., Dewan, C., Diab, M., Li, X., Lin, X.V., et al. (2022). OPT: Open Pre-trained Transformer Language Models. Preprint at arXiv. https://doi.org/10.48550/arXiv.2205.01068.

14. Lieber, O., Sharir, O., Lenz, B., and Shoham, Y. (2021). Jurassic-1: Technical details and evaluation. White Paper AI21 Labs *1*, 9.

15. Fedus, W., Zoph, B., and Shazeer, N. (2022). Switch transformers: Scaling to trillion parameter models with simple and efficient sparsity. J. Mach. Learn. Res. *23*, 5232–5270.

16. Laurençon, H., Saulnier, L., Wang, T., Akiki, C., Villanova del Moral, A., Le Scao, T., Von Werra, L., Mou, C., González Ponferrada, E., Nguyen, H., et al. (2022). The BigScience ROOTS Corpus: A 1.6TB composite multilingual dataset. Adv. Neural Inf. Process. Syst. *35*, 31809–31826.

17. Kaplan, J., McCandlish, S., Henighan, T., Brown, T.B., Chess, B., Child, R., Gray, S., Radford, A., Wu, J., et al. (2020). Scaling Laws for Neural

Language Models. Preprint at arXiv. https://doi.org/10.48550/arXiv.2001.08361.

18. Srivastava, A., Rastogi, A., Rao, A., Shoeb, A.A.M., Abid, A., Fisch, A., Brown, A.R., Santoro, A., Gupta, A., Garriga-Alonso, A., et al. (2022). Beyond the Imitation Game: Quantifying and extrapolating the capabilities of language models. Preprint at arXiv. https://doi.org/10.48550/arXiv.2206.04615.
19. Liu, P., Yuan, W., Fu, J., Jiang, Z., Hayashi, H., and Neubig, G. (2023). Pre-train, prompt, and predict: A systematic survey of prompting methods in natural language processing. ACM Comput. Surv. *55*, 1–35.
20. Wei, J., Wang, X., Schuurmans, D., Bosma, M., Xia, F., Chi, E., Le, Q.V., and Zhou, D. (2022). Chain-of-thought prompting elicits reasoning in large language models. Adv. Neural Inf. Process. Syst. *35*, 24824–24837.
21. Zhou, D., Schärli, N., Hou, L., Wei, J., Scales, N., Wang, X., Schuurmans, D., Cui, C., Bousquet, O., Le, Q., and Chi, E. (2022). Least-to-most Prompting Enables Complex Reasoning in Large Language Models. Preprint at arXiv. https://doi.org/10.48550/arXiv.2205.10625.
22. Drozdov, A., Schärli, N., Akyürek, E., Scales, N., Song, X., Chen, X., Bousquet, O., and Zhou, D. (2022). Compositional Semantic Parsing with Large Language Models. Preprint at arXiv. https://doi.org/10.48550/arXiv.2209.15003.
23. Nye, M.I., Andreassen, A.J., Gur-Ari, G., Michalewski, H., Austin, J., Bieber, D., Dohan, D., Lewkowycz, A., Bosma, M., Luan, D., et al. (2021). Show Your Work: Scratchpads for Intermediate Computation with Language Models. Preprint at arXiv. https://doi.org/10.48550/arXiv.2112.00114.
24. Suzgun, M., Scales, N., Schärli, N., Gehrmann, S., Tay, Y., Chung, H.W., Chowdhery, A., Le, Q.V., Chi, E.H., Zhou, D., and Wei, J. (2022). Challenging BIG-bench Tasks and Whether Chain-of-thought Can Solve Them. Preprint at arXiv. https://doi.org/10.48550/arXiv.2210.09261.
25. Kojima, T., Gu, S.S., Reid, M., Matsuo, Y., and Iwasawa, Y. (2022). Large language models are zero-shot reasoners. Adv. Neural Inf. Process. Syst. *35*, 22199–22213.
26. Ouyang, L., Wu, J., Jiang, X., Almeida, D., Wainwright, C., Mishkin, P., Zhang, C., Agarwal, S., Slama, K., Ray, A., Schulman, J., et al. (2022). Training language models to follow instructions with human feedback. Adv. Neural Inf. Process. Syst. *35*, 27730–27744.
27. Bender, E.M., Gebru, T., McMillan-Major, A., and Shmitchell, S. (2021). On the dangers of stochastic parrots: Can language models be too big? In Proceedings of the 2021 ACM conference on fairness, accountability, and transparency, pp. 610–623.
28. Wang, B., Xu, C., Wang, S., Gan, Z., Cheng, Y., Gao, J., Awadallah, A.H., and Li, B. (2021). Adversarial GLUE: A Multi-task Benchmark for Robustness Evaluation of Language Models. Preprint at arXiv. https://doi.org/10.48550/arXiv.2111.02840.
29. Korngiebel, D.M., and Mooney, S.D. (2021). Considering the possibilities and pitfalls of Generative Pre-trained Transformer 3 (GPT-3) in healthcare delivery. NPJ Digit. Med. *4*, 93.
30. Sezgin, E., Sirrianni, J., and Linwood, S.L. (2022). Operationalizing and Implementing Pretrained, Large Artificial Intelligence Linguistic Models in the US Health Care System: Outlook of Generative Pretrained Transformer 3 (GPT-3) as a Service Model. JMIR medical informatics, 10.
31. Hendrycks, D., Burns, C., Basart, S., Zou, A., Mazeika, M., Song, D., and Steinhardt, J. (2020). Measuring Massive Multitask Language Understanding. Preprint at arXiv. https://doi.org/10.48550/arXiv.2009.03300.
32. Jin, D., Pan, E., Oufattole, N., Weng, W.H., Fang, H., and Szolovits, P. (2021). What disease does this patient have? a large-scale open domain question answering dataset from medical exams. Appl. Sci. *11*, 6421.
33. Singhal, K., Azizi, S., Tu, T., Mahdavi, S.S., Wei, J., Chung, H.W., Scales, N., Tanwani, A., Cole-Lewis, H., Pfohl, S., et al. (2023). Large language models encode clinical knowledge. Nature *620*, 172–180.
34. Singhal, K., Tu, T., Gottweis, J., Sayres, R., Wulczyn, E., Hou, L., Clark, K., Pfohl, S., Cole-Lewis, H., Neal, D., et al. (2023). Towards Expert-level Medical Question Answering with Large Language Models. Preprint at arXiv. https://doi.org/10.48550/arXiv.2305.09617.
35. Nori, H., King, N., McKinney, S.M., Carignan, D., and Horvitz, E. (2023). Capabilities of GPT-4 on Medical Challenge Problems. Preprint at arXiv. https://doi.org/10.48550/arXiv.2303.13375.
36. Gilson, A., Safranek, C.W., Huang, T., Socrates, V., Chi, L., Taylor, R.A., and Chartash, D. (2023). How Does ChatGPT Perform on the United States Medical Licensing Examination? The Implications of Large Language Models for Medical Education and Knowledge Assessment. JMIR Med. Educ. *9*, e45312.
37. Kung, T.H., Cheatham, M., Medenilla, A., Sillos, C., De Leon, L., Elepaño, C., Madriaga, M., Aggabao, R., Diaz-Candido, G., Maningo, J., and Tseng, V. (2023). Performance of ChatGPT on USMLE: Potential for AI-assisted medical education using large language models. PLOS Digit. Health 2, e0000198.
38. Touvron, H., Martin, L., Stone, K., Albert, P., Almahairi, A., Babaei, Y., Bashlykov, N., Batra, S., Bhargava, P., Bhosale, S., et al. (2023). Llama 2: Open Foundation and Fine-tuned Chat Models. Preprint at arXiv. https://doi.org/10.48550/arXiv.2307.09288.
39. Chen, Z., Hernández-Cano, A., Romanou, A., Bonnet, A., Matoba, K., Salvi, F., Pagliardini, M., Fan, S., Köpf, A., Mohtashami, A., et al. (2023). MEDITRON-70B: Scaling Medical Pretraining for Large Language Models. Preprint at arXiv. https://doi.org/10.48550/arXiv.2311.16079.
40. Wang, Y., Ma, X., and Chen, W. (2023). Augmenting Black-box LLMs with Medical Textbooks for Clinical Question Answering. Preprint at arXiv. https://doi.org/10.48550/arXiv.2309.02233.
41. Liévin, V., Motzfeldt, A.G., Jensen, I.R., and Winther, O. (2023). Variational open-domain question answering. In International Conference on Machine Learning (PMLR), pp. 20950–20977.
42. Wang, X., Wei, J., Schuurmans, D., Le, Q.V., Chi, E.H., and Zhou, D. (2022). Self-consistency Improves Chain of Thought Reasoning in Language Models. Preprint at arXiv. https://doi.org/10.48550/arXiv.2203.11171.
43. Li, Y., Lin, Z., Zhang, S., Fu, Q., Chen, B., Lou, J., and Chen, W. (2022). On the Advance of Making Language Models Better Reasoners. Preprint at arXiv. https://doi.org/10.48550/arXiv.2206.02336.
44. Pal, A., Umapathi, L.K., and Sankarasubbu, M. (2022). Medmcqa: A large-scale multi-subject multi-choice dataset for medical domain question answering. PMLR *174*, 248–260.
45. Jin, Q., Dhingra, B., Liu, Z., Cohen, W.W., and Lu, X. (2019). PubMedQA: A Dataset for Biomedical Research Question Answering. Preprint at arXiv. https://doi.org/10.48550/arXiv.1909.06146.
46. Chung, H.W., Hou, L., Longpre, S., Zoph, B., Tay, Y., Fedus, W., et al. (2022). Scaling Instruction-finetuned Language Models. Preprint at arXiv. https://doi.org/10.48550/arXiv.2210.11416.
47. Lewis, P., Perez, E., Piktus, A., Petroni, F., Karpukhin, V., Goyal, N., Küttler, H., Lewis, M., Yih, W.T., Rocktäschel, T., et al. (2020). Retrieval-augmented generation for knowledge-intensive nlp tasks. Adv. Neural Inf. Process. Syst. *33*, 9459–9474.
48. Borgeaud, S., Mensch, A., Hoffmann, J., Cai, T., Rutherford, E., Millican, K., Van Den Driessche, G.B., Lespiau, J.B., Damoc, B., Clark, A., et al. (2022). Improving language models by retrieving from trillions of tokens. PMLR *162*, 2206–2240.
49. Lazaridou, A., Gribovskaya, E., Stokowiec, W., and Grigorev, N. (2022). Internet-augmented language models through few-shot prompting for open-domain question answering. Preprint at arXiv. https://doi.org/10.48550/arXiv.2203.05115.
50. Liévin, V. (2023). Vlievin/Medical-Reasoning: Official Release (Zenodo). https://doi.org/10.5281/zenodo.10301874.
51. Ott, S., Hebenstreit, K., Li'evin, V., Hother, C.E., Moradi, M., Mayrhauser, M., Praas, R., Winther, O., and Samwald, M. (2023). ThoughtSource: A central hub for large language model reasoning data. Sci. Data, 10.
52. Chen, M., Tworek, J., Jun, H., Yuan, Q., de Oliveira Pinto, H.P., Kaplan, J., Edwards, H., Burda, Y., Joseph, N., Brockman, G., et al. (2021). Evaluating

CellPress
OPEN ACCESS

Large Language Models Trained on Code. Preprint at arXiv. https://doi.org/10.48550/arXiv.2107.03374.

53. Zheng, L., Chiang, W., Sheng, Y., Zhuang, S., Wu, Z., Zhuang, Y., Lin, Z., Li, Z., Li, D., Xing, E., et al. (2023). Judging LLM-as-a-judge with MT-bench and Chatbot Arena. Preprint at arXiv. https://doi.org/10.48550/arXiv.2306.05685.

54. Dettmers, T., Pagnoni, A., Holtzman, A., and Zettlemoyer, L. (2023). QLoRA: Efficient Finetuning of Quantized LLMs. Preprint at arXiv. https://doi.org/10.48550/arXiv.2305.14314.

55. Almazrouei, E., Alobeidli, H., Alshamsi, A., Cappelli, A., Cojocaru, R., Debbah, M., Goffinet, E., Heslow, D., Launay, J., Malartic, Q., et al. (2023). Falcon-40B: an open large language model with state-of-the-art performance. https://falconllm.tii.ae.

56. Team, M.N. (2023). Introducing Mpt-30b: Raising the Bar for Open-Source Foundation Models. www.mosaicml.com/blog/mpt-30b. (Accessed 22 June 2023). Accessed.

57. Black, S., Biderman, S., Hallahan, E., Anthony, Q., Gao, L., Golding, L., He, H., Leahy, C., McDonell, K., Phang, J., et al. (2022). GPT-NeoX-20B: An Open-source Autoregressive Language Model. Preprint at arXiv. https://doi.org/10.48550/arXiv.2204.06745.

58. Yasunaga, M., Leskovec, J., and Liang, P. (2022). LinkBERT: Pretraining Language Models with Document Links. Preprint at arXiv. https://doi.org/10.48550/arXiv.2203.15827.

59. Karpukhin, V., Oguz, B., Min, S., Wu, L., Edunov, S., Chen, D., and Yih, W.T. (2020). Dense Passage Retrieval for Open-domain Question Answering. Preprint at arXiv. https://doi.org/10.48550/arXiv.2004.04906.

60. Cobbe, K., Kosaraju, V., Bavarian, M., Chen, M., Jun, H., Kaiser, L., Plappert, M., Tworek, J., Hilton, J., Nakano, R., et al. (2021). Training Verifiers to Solve Math Word Problems. Preprint at arXiv. https://doi.org/10.48550/arXiv.2110.14168.

61. Kadavath, S., Conerly, T., Askell, A., Henighan, T., Drain, D., Perez, E., Plappert, M., Tworek, J., Hilton, J., Nakano, R., et al. (2022). Language Models (Mostly) Know What They Know. Preprint at arXiv. https://doi.org/10.48550/arXiv.2207.05221.

62. Zhou, C., Liu, P., Xu, P., Iyer, S., Sun, J., Mao, Y., Ma, X., Efrat, A., Yu, P., Yu, L., et al. (2023). LIMA: Less Is More for Alignment. Preprint at arXiv. https://doi.org/10.48550/arXiv.2305.11206.

63. Singhal, K., Tu, T., Gottweis, J., Sayres, R., Wulczyn, E., Hou, L., Clark, K., Pfohl, S., Cole-Lewis, H., Neal, D., et al. (2023). Towards Expert-level Medical Question Answering with Large Language Models. Preprint at arXiv. https://doi.org/10.48550/arXiv.2305.09617.

Patterns, Volume *5*

Supplemental information

Can large language models

reason about medical questions?

Valentin Liévin, Christoffer Egeberg Hother, Andreas Geert Motzfeldt, and Ole Winther

A Summary of the Results

In Table S2, we summarize the performances of InstructGPT and Codex on the MMLU-USMLE, MedQA-USMLE, MedMCQA and PubMedQA datasets in zero-shot, few-shot, with and without grounding. All our results on the validation set of the MedMCQA are estimated using 1k samples. The results of the MedMCQA test set require submitting an official submission. We used a sampling temperature of $\tau = 0$ for all experiments except when drawing $k > 0$ samples and using majority voting (MV). For the majority voting model, we used $k = 100$ samples and $\tau = 0.5$ for Codex, $\tau = 0.9$ for Vicuna.

B Domain-specific CoT cues

Table S1: Validation performances for 30 CoT cues on a subset of 100 validation USMLE questions.

	CoT cue	Accuracy	F1	CoT length
0	*Let's derive the differential diagnosis step by step*	**48.0**	48.0	170
1	*Let's use step by step inductive reasoning, given the medical nature of the question*	**48.0**	**48.2**	157
2	*Let's differentiate using step by step reasoning like a medical expert*	47.0	46.3	183
3	*Let's think step by step using deductive reasoning*	47.0	46.4	148
4	*Let's differentiate using step by step reasoning*	45.0	45.0	166
5	*Let's think step by step to arrive at one of the options*	45.0	45.0	158
6	*Let's break the problem into multiple steps*	45.0	44.2	165
7	*Let's use step by step deductive reasoning, given the medical nature of the question*	44.0	44.0	174
8	*Let's think step by step like a doctor*	43.0	43.3	162
9	*Let's think step by step like a medical expert*	43.0	42.8	171
10	*Let's summarize the facts step by step*	42.0	42.1	183
11	*Let's think step by step using inductive reasoning*	42.0	42.6	143
12	*Let's think step by step using deductive reasoning like a medical expert*	42.0	42.3	173
13	*Let's be concise and think step by step*	42.0	42.4	130
14	*Let's differentiate using step by step deductive reasoning like a medical expert*	42.0	41.9	173
15	*Let's argue step by step*	42.0	42.2	149
16	*Let's think step by step like a clinician*	41.0	41.3	164
17	*Let's think step by step*	40.0	40.4	129
18	*Let's reflect on each answer option step by step*	40.0	37.2	194
19	*Let's reason and differentiate options step by step like a medical expert*	40.0	38.1	180
20	*Let's differentiate using step by step inductive reasoning like a medical expert*	40.0	39.5	161
21	$\varnothing$ *(Direct)*	39.0	38.4	0
22	*Let's think step by step given every option equal consideration*	39.0	39.2	177
23	*Let's think step by step like a scientist*	39.0	39.2	166
24	*Let's use step by step inductive reasoning*	37.0	36.1	165
25	*Let's work by elimination step by step*	36.0	35.2	154
26	*Let's use step by step deductive reasoning*	34.0	33.9	165
27	*Let's follow a Bayesian step by step approach*	33.0	31.4	193
28	*Let's reflect on each option from the least likely to the most likely*	31.0	27.9	166
29	*Let's use step by step Bayesian reasoning, given the medical nature of the question*	31.0	30.7	216

We composed an initial set of 30 zero-shot CoT prompt variations. In Table S1, we report the accuracy for each of the 30 prompts based on a subset of 100 USMLE validation questions. Given an estimated accuracy uncertainty of 5% (see the paragraph "uncertainty estimation" below), we concluded that the first half of the results are all reasonable candidates for the study.

Selected prompts For the remaining of this paper, we selected 5 prompts: the original "*Let's think step by step*", the medical variation "*Let's think step by step like a medical expert*" and the top-three CoT cues reported in Table S1.

Prompt diversity and agreement In Figure S1, we report the agreement rate for all 30 prompts on the 100 validation questions. Whereas most of the prompts followed a rather consistent pattern, with an agreement rate superior to 50%, a minority of the prompts seemed to agree less with the majority of the prompts, such as “Let’s reflect on each answer option step by step”, “Let’s follow a Bayesian step by step approach” or “Let’s work by elimination”. In Figure S4, we showcase four chain-of-thoughts selected to highlight the diversity of the completions and the ability of InstructGPT to adopt diverse problem-solving strategies. Yet, strategies are not always executed correctly: in Figure S4, example 2, GPT-3 ultimately finds the correct answer (Missense mutation) but identified the wrong diagnostic (the 6-year-old boy suffers from sickle cell disease).

Uncertainty estimation We model the outcome of answering a question using a $\mathrm{Bernoulli}$ model with parameter θ where 1 corresponds to the correct predicted answer, and 0 corresponds to predicting the wrong answer. The accuracy of the model corresponds to the mean outcome of the $\mathrm{Bernoulli}$ model ($\mathbb{E}\left[\mathrm{Bernoulli}(\theta)\right] = \theta$) that we approximate as $\theta = 0.5$. Given N=100 data points, the uncertainty of the accuracy estimate is about 5%, as given by the standard deviation of the mean estimator:

$$\sqrt{\mathrm{Var}_N\left[\mathrm{Bernoulli}(\theta)\right]} = \sqrt{\frac{\theta(1-\theta)}{N}} = 0.5^2/100 = 0.05\ (5\%)\ .$$

Figure S1: Rate of agreement for the 30 evaluated CoT prompts evaluated in Table S1.

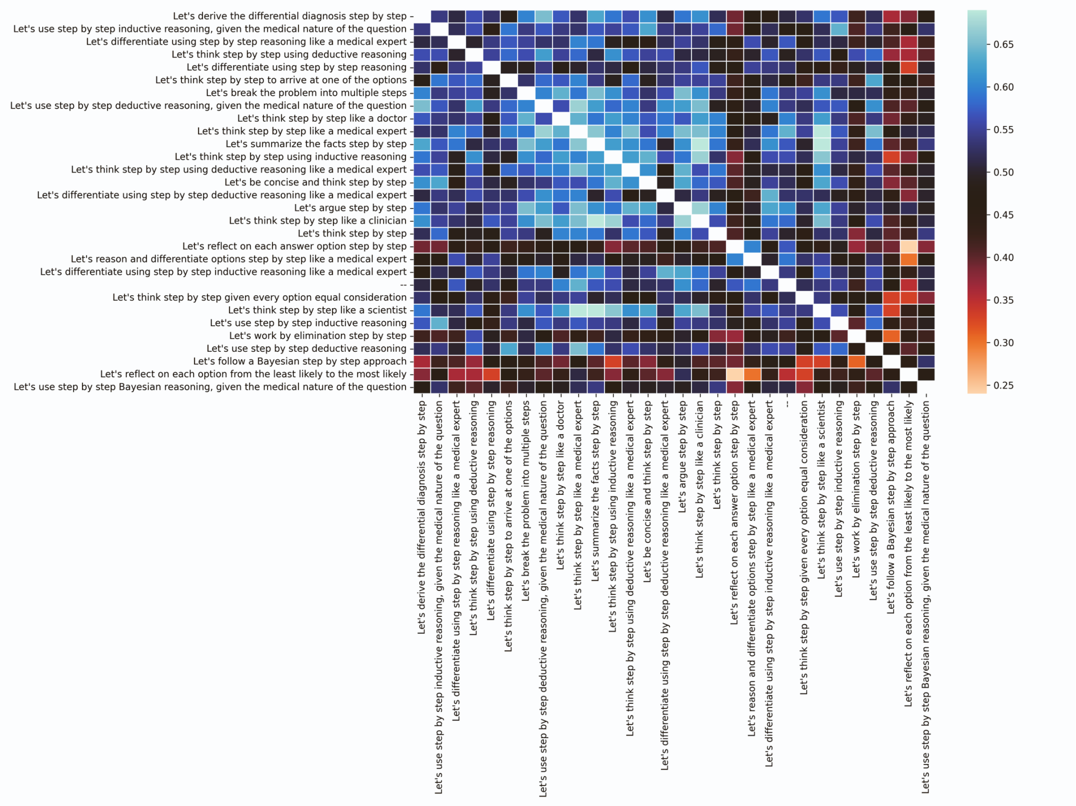

Table S2: Question answering accuracy on the MedQA-USMLE, MedMCQA (valid. 1k samples/test), and PubMedQA datasets. The CoTs cues #1–5 are defined in Table 3 (e.g., #2 = *Let's think step by step like a medical expert*). Results marked with ⋆ represent the pinnacles of our observations.

Model	Shots	Grounding	Prompt	MMLU⋆	USMLE	MedMCQA	PubMedQA
InstructGPT (175B)	0	✗	Direct	–/ –	46.0	44.0/ –	73.2
InstructGPT (175B)	0	✗	CoT #1	–/ –	47.1	40.8/ –	60.0
InstructGPT (175B)	0	✗	CoT #2	–/ –	46.8	43.3/ –	59.8
InstructGPT (175B)	0	✗	CoT #3	–/ –	46.0	38.8/ –	66.2
InstructGPT (175B)	0	✗	CoT #4	–/ –	45.6	37.1/ –	58.0
InstructGPT (175B)	0	✗	CoT #5	–/ –	45.1	42.1/ –	55.6
InstructGPT (175B)	0	✓	Direct	–/ –	47.3	46.7/ 49.0	–
InstructGPT (175B)	0	✓	CoT #1	–/ –	45.9	42.2/ 46.0	–
InstructGPT (175B)	0	✓	CoT #2	–/ –	47.0	45.8/ 46.0	–
InstructGPT (175B)	0	✓	CoT #3	–/ –	45.6	41.6/ 43.3	–
InstructGPT (175B)	0	✓	CoT #4	–/ –	45.9	41.3/ 45.0	–
InstructGPT (175B)	0	✓	CoT #5	–/ –	47.4	41.8/ 46.5	–
InstructGPT (175B)	0	✗	Ensemble (n=6)	–/ –	50.0	42.4/ –	70.4
InstructGPT (175B)	0	✓	Ensemble (n=6)	–/ –	49.3	48.8/ 49.5	–
InstructGPT (175B)	0	✗ + ✓	Ensemble (n=12)	–/ –	53.1	47.6/ –	–
Codex (external results)[1]	0	✗	Direct	–/ –	–	–/ 54.4	–
Codex (175B)	0	✗	Direct	74.2/ 70.6	52.5	50.9/ 51.1	73.2
Codex (175B)	0	✗	CoT #1	64.5/ 60.7	52.9	46.8/ –	53.4
Codex (175B)	0	✓	Direct	64.5/ 68.7	52.5	50.8/ 52.7	–
Codex (175B)	0	✓	CoT #1	77.4/ 55.1	47.2	43.9/ –	–
Codex (175B)	5	✗	Direct	77.2/ 70.1	56.6	56.6/ 56.9	73.0
Codex (175B)	5	✗	CoT #1	⋆80.6/ 68.4	56.2	52.0/ –	76.4
Codex (175B)	5	✗	CoT #1 + MV(k=20)▽	74.2/ ⋆**76.8**	57.2	57.6/ 57.5	⋆78.2
Codex (175B)	5	✗	CoT #1 + MV(k=100)▽	–/ –	60.2	⋆59.7/ ⋆62.7	78.0
GPT-4	5	✗	CoT[2]	–	86.1	**73.7**/ –	77.4
GPT-NeoX (20B)[3]	0	✗	Direct	–	26.9	27.8/ –	–
GPT-NeoX (20B)	5	✗	CoT #1	–	28.0	32.9/ –	–
MPT-Instruct (7B)[4]	0	✗	Direct	–	23.9	23.2/ –	–
MPT-Instruct (7B)	5	✗	CoT #1	–	28.8	31.7/ –	–
MPT-Instruct (30B)	0	✗	Direct	–	35.1	34.6/ –	–
MPT-Instruct (30B)	5	✗	CoT #1	–	40.1	40.3/ –	–
Falcon-Instruct (7B)[5]	0	✗	Direct	–	25.3	25.2/ –	–
Falcon-Instruct (7B)	5	✗	CoT #1	–	24.0	23.8/ –	–
Falcon-Instruct (40B)	0	✗	Direct	–	39.0	30.0/ –	–
Falcon-Instruct (40B)	5	✗	CoT #1	–	40.3	44.0/ –	–
Guanaco (33B)[6]	0	✗	Direct	–	42.9	37.4/ –	–
Guanaco (33B)	5	✗	CoT #1	–	48.0	40.3/ –	–
Guanaco (65B)	0	✗	Direct	–	40.8	36.7/ –	–
Guanaco (65B)	5	✗	CoT #1	–	49.9	43.3/ –	–
Vicuna 1.3 (7B)[7]	0	✗	Direct	–	27.2	21.2/ –	–
Vicuna 1.3 (7B)	5	✗	CoT #1	–	39.7	33.6/ –	–
Vicuna 1.3 (13B)	0	✗	Direct	–	38.7	38.3/ –	–
Vicuna 1.3 (13B)	5	✗	CoT #1	–	46.4	43.6/ –	–
Vicuna 1.3 (33B)	0	✗	Direct	–	45.2	38.0/ –	–
Vicuna 1.3 (33B)	5	✗	CoT #1	–	49.2	41.3/ –	–
Vicuna 1.3 (33B)	5	✗	CoT #1 + MV(k=12)▽	–	52.2	44.7/ –	–
Llama-2 (7B)[8]	0	✗	Direct	–	26.1	22.6/ –	–
Llama-2 (7B)	5	✗	CoT #1	–	34.1	36.2/ –	–
Llama-2 (7B)	5	✗	CoT #1 + MV(k=100)▽	–	37.6	37.5/ –	–
Llama-2 (13B)	0	✗	Direct	–	31.1	31.7/ –	–
Llama-2 (13B)	5	✗	CoT #1	–	40.0	42.8/ –	–
Llama-2 (13B)	5	✗	CoT #1 + MV(k=100)▽	–	46.7	45.5/ –	–
Llama-2 (70B)	0	✗	Direct	–	43.4	42.8/ –	–
Llama-2 (70B)	5	✗	CoT #1	–	57.4	53.6/ –	–
Llama-2 (70B)	5	✗	CoT #1 + MV(k=50)▽	–	⋆62.5	–/ –	–
Llama-2-chat (7B)	0	✗	Direct	–	29.7	35.6/ –	–
Llama-2-chat (7B)	5	✗	CoT #1	–	32.9	33.2/ –	–
Llama-2-chat (13B)	0	✗	Direct	–	32.2	36.6/ –	–
Llama-2-chat (13B)	5	✗	CoT #1	–	44.5	44.6/ –	–
Llama-2-chat (70B)	0	✗	Direct	–	42.3	41.8/ –	–
Llama-2-chat (70B)	5	✗	CoT #1	–	43.4	44.9/ –	–
Vicuna 1.5 (7B)[7]	0	✗	Direct	–	37.1	35.5/ –	–
Vicuna 1.5 (7B)	5	✗	CoT #1	–	40.5	41.2/ –	–
Vicuna 1.5 (13B)	0	✗	Direct	–	41.6	41.5/ –	–
Vicuna 1.5 (13B)	5	✗	CoT #1	–	50.8	46.0/ –	–
Vicuna 1.5 (13B)	0	✗	Direct + MV(k=100)▽	–	41.7	42.6/ –	–
Vicuna 1.5 (13B)	5	✗	CoT #1 + MV(k=100)▽	–	50.4	46.3/ –	–
U-PaLM (540B)[9]	5	✗	Direct	87.1/ –	–	–/ –	–
U-PaLM (540B)[9]	5	✗	CoT #1	58.1/ –	–	–/ –	–
Flan-U-PaLM (540B)[9]	5	✗	Direct	**90.3**/ –	–	–/ –	–
Flan-U-PaLM (540B)[9]	5	✗	CoT #1	80.6/ –	–	–/ –	–
Med-PaLM V2 (540B)[10]	*finetuned*	✗	–	–	**86.5**	72.3/ –	**81.8**
PubMedBERT (110M)[11]	*finetuned*	✗	–	–/ –	–	40.0/ 41.0	–
PubMedBERT (110M)[12]	*finetuned*	✓	–	–/ –	–	43.0/ 47.0	–
BioLinkBERT (345M)[13]	*finetuned*	✓	–	–/ 50.7	44.6	–/ –	72.2
BioGPT (347M)[14]	*finetuned*	✗	–	–/–	–	–/–	78.2
PubMedGPT (2.7B)[15]	*finetuned*	✗	–	–/–	50.3	–/–	74.4
Galactica (120B)[16]	*finetuned*	✗	–	–/–	44.4	52.9/ –	77.6
Human (passing score)	–	–	–	60.0†	60.0†	50.0§,⋄	–
Human (expert score)	–	–	–	87.0‡	87.0‡	90.0[12]	78.0[17]

† USMLE (passing score): https://www.usmle.org/scores-transcripts ‡ USMLE (expert score): 95th percentile ≈ 87%[18]
§ MedMCQA test (AIIMS): https://collegedunia.com/exams/aiims-mbbs/cutoff ⋆ *professional medicine subset* (USMLE)[18]
⋄ MedMCQA valid. (NEET PG): https://medicine.careers360.com/articles/neet-pg-cut-off
▽ Majority voting classifier with k samples and temperature $\tau = 0.5$ for Codex, $\tau = 0.9$ for all other models (self-consistency[19])

C Datasets

MedQA-USMLE The study by Jin et al.[20] gathers historical questions from the United States Medical Licensing Examination (USMLE), which targets trained medical professionals. The questions are notorious for being challenging as they often require strong problem-solving skills coupled with comprehensive medical knowledge. Each question features a description of a medical case and a question that emulates the real clinical setting. The more recent MMLU dataset has 31 validation and 272 test USMLE questions (around 105 words/question).[18] In Appendix D, we benchmark both USMLE datasets and found the MedQA USMLE dataset to be more difficult. The MedQA-USMLE data does not come with explanations. Instead, we use the MMLU-USMLE CoTs that are available from `https://github.com/jasonwei20/flan-2`.[9]

MedMCQA MedMCQA is a large-scale multiple-choice question answering collected from Indian medical school entrance exams (AIIMS and NEET-PG).[12] The MedMCQA covers a broad range of medical topics (dentistry, psychiatry, surgery, ...) and requires being able to follow a variety of reasoning types (logic, factual, comparison, ...). However, questions are often more knowledge-centred than the USMLE questions, which tend to focus more on problem-solving skills.

PubMedQA PubMedQA is a collection of expert-annotated yes/no/maybe research questions derived from PubMed abstracts.[17] Whereas the questions from the USMLE and the MedMCQA datasets are self-contained and might be answered using general medical knowledge and methodology, each PubMedQA question is contextualized on a provided abstract. Therefore PubMedQA primarily focuses on evaluating reading comprehension skills.

D MedQA-USMLE versus MMLU-USMLE

Table S3: Comparing the USMLE datasets from (test) MedQA[20] and (validation/test) MMLU[18]. We include the results of the recent Flan-U-PaLM 540B[9]. All models use 5 shots.

Model	Prompt	MMLU	MedQA
Codex	Direct	77.2 / **70.1**	**56.6**
Codex	CoT #1	80.6 / 69.1	56.2
U-PaLM	Direct	87.1 / –	–
U-PaLM	CoT #1	58.1 / –	–
Flan-U-PaLM	Direct	**90.3** / –	–
Flan-U-PaLM	CoT #1	80.6 / –	–
Human (passing score)		60.0	60.0
Human (expert score)		87.0	87.0

In Table S3, we report the performances of the three medical question answering datasets as well as the *professional medicine* subset of the MMLU dataset[18], which was also explored in recent related work.[9]

Based on Codex performances, the MedQA-USMLE dataset appears to be more challenging than the MMLU-USMLE counterpart. Codex in a 5-shot setting (Direct and CoT prompting, τ=0), scores around 13.2% lower accuracy on the MedQA-USMLE (∼56.4%) than on the MMLU-USMLE (∼69.6%). Succeeding the USMLE requires a score of around 60%.

E Comparing GPT versions on the USMLE dataset

We report the test USMLE accuracy for multiple GPT version in Table S4 for the direct and CoT #1 prompts. Note that Codex (code-davinci-002) is a large GPT-3 model pre-trained on text and code; InstructGPT (text-davinci-002) is a version of Codex finetuned based human-feedback to "follow the user's instructions helpfully and safely".

Table S4: Answering accuracy of multiple GPT-3 models on the USMLE dataset in a zero-shot setting.

**Model**	**Prompt**	**Acc.**	Δ
text-curie-001	Direct	27.8	-9.4
text-davinci-001	Direct	37.2	–
code-davinci-002	Direct	52.5	+15.3
text-davinci-002	Direct	46.0	+8.8
text-curie-001	CoT #1	25.5	-14.7
text-davinci-001	CoT #1	40.2	–
code-davinci-002	CoT #1	52.9	+12.7
text-davinci-002	CoT #1	47.1	+6.9
Random		25.0	

The smallest model performed only slightly better than at random, with an accuracy of maximum 27.8% for the curie model, whereas the largest model non-aligned text model text-davinci-001 scored a maximum of 40.2% for all prompts. The best performances are obtained with the text and code pre-trained model code-davinici-002 (52.9%). Human-alignment appears to damage answering performances: text-davinci-002 scored a maximum of 47.1%. This suggests that advanced medical reasoning capabilities only emerge in the largest of the GPT-3 models, and that code pre-trained is highly effective. In this experiment, human-alignment led to a decrease of accuracy, although we found InstructGPT to overall produce more readable samples than Codex in zero-shot CoT settings (Appendix I).

F Answering bias

Table S5: Frequencies of predictions and labels. Classification bias of InstructGPT and Codex on the USMLE dataset, with (✓) and without (✗) random label permutation. We highlight labels that are under estimated using the color blue ▾ and over estimated using the color red ▴ ($\pm$ 10% of the label frequency). Using the χ^2 test, we report the pp-value for the null hypothesis "*the predictive distribution equals the empirical one*". The models are evaluated using zero shot and $T = 0$, unless specified.

Perm.	Model	Prompt	A	B	C	D	Acc.	p-value
✗	InstructGPT	Direct	155▾	299	405▴	414▴	46.0	$< 10^{-10}$
✗	InstructGPT	CoT #1	421▴	240▾	291▾	321▴	47.1	$1 \cdot 10^{-10}$
✗	InstructGPT	CoT #2	423▴	211▾	286▾	353▴	46.8	$< 10^{-10}$
✗	InstructGPT	CoT #3	416▴	236▾	272▾	349▴	46.0	$< 10^{-10}$
✗	InstructGPT	CoT #4	378	221▾	294▾	380▴	45.6	$< 10^{-10}$
✗	InstructGPT	CoT #5	392▴	234▾	277▾	370▴	45.1	$< 10^{-10}$
✗		**data**	**353**	**309**	**346**	**265**		
✓	InstructGPT	Direct	138▾	295	377▴	463▴	46.5	$< 10^{-10}$
✓	InstructGPT	CoT #1	374▴	276▾	252▾	371▴	45.3	$4 \cdot 10^{-10}$
✓		**data**	**317**	**326**	**323**	**307**		
✗	Codex	Direct	163▾	360▴	407▴	343▴	52.1	$< 10^{-10}$
✗	Codex (5 shots)	Direct	254▾	285▾	430▴	304▴	56.6	$< 10^{-10}$
✗	Codex	CoT #1	315	250▾	285	423▴	52.9	$< 10^{-10}$
✗	Codex (5 shots)	CoT #1	334	300	324	315▴	56.2	$7 \cdot 10^{-03}$
✗	Codex (5 shots, τ=0.5)[1]	CoT #1	340	281	308▾	349▴	60.2	$2 \cdot 10^{-06}$
✗		**data**	**353**	**309**	**346**	**265**		

[1]Averaged using $k = 100$ samples.

Figure S2: Frequencies of predicted labels for Codex 5-shot CoT (average of k=100 samples) and ground truth label frequencies. For the USMLE, we report frequencies of the zero-shot InstructGPT (Direct and CoT prompting), originally displayed in Figure 4.

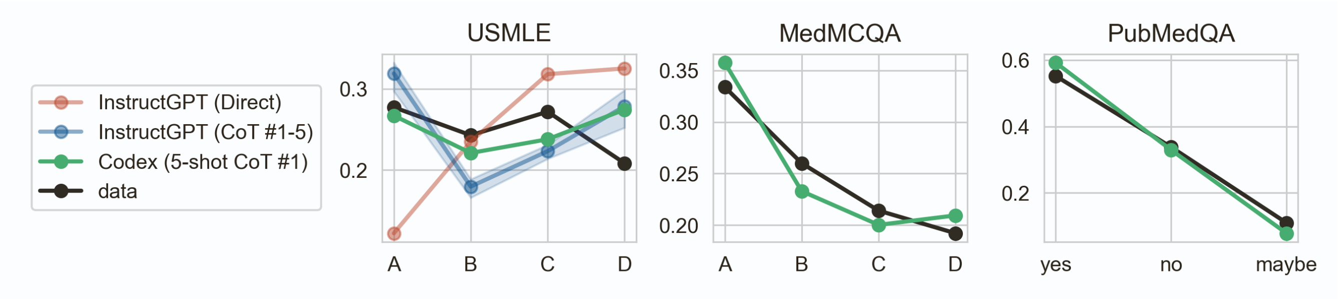

In Table S5, we report the frequencies of answers and of predicted labels with and without label permutation. We report the frequencies for InstructGPT as well as Codex.

Querying InstructGPT using the CoT prompts resulted in a more faithful predictive distribution of the labels. Nonetheless, a bias towards the labels A and D and a tendency to avoid predicting labels B and C could still be observed. To confirm whether this bias originates from the data or the model, we permuted the labels and repeated the experiment for prompts number 0 and 1 and observed the same trend. Codex exhibits similar trends, although few-shot learning seems to yield more faithful predictive distributions.

In all cases, models tend to default to the label D. In Figure S7, we present two CoT leading to mispredicted label D. In both cases, GPT-3 fails to narrow down to one answer options and defaults to option D.

Figure S2 presents some of the results from Table S5 for the USMLE and extend it with the frequencies observed in the two other datasets for Codex 5-shot CoT ($k = 100$ samples). The bias appears less important for the MedMCQA and PubMedQA datasets than for the USMLE dataset.

G Information retrieval

Wikipedia articles were converted into overlapping passages of size 100 words and indexed along with their respective article titles. Given a question $\mathbf{q}$, an answer choice $\mathbf{a}$, and weights $\beta_1 = 1, \beta_2 = 1, \beta_3 = 0.5$. The weights were chosen based on a qualitative assessment of the retrieved passages on a few questions. we retrieved passages $\mathbf{d}$ based on a composite BM25 score defined as

$$\text{score}(\mathbf{q}, \mathbf{a}, \mathbf{d}) = \beta_1 \cdot \text{BM25}(\mathbf{q}, \mathbf{d}_{\text{content}}) + \beta_2 \cdot \text{BM25}(\mathbf{a}, \mathbf{d}_{\text{content}}) + \beta_3 \cdot \text{BM25}(\mathbf{a}, \mathbf{d}_{\text{title}}) \,. \tag{2}$$

H Open-Source LLMs

We assessed the performances of open-source models (Vicuna, Guanaco, GPT-NeoX, MPT-instruct, Falcon and Llama-2) on the MedQA-USMLE and MedMCQA datasets in zero-shot and 5-shot settings, all using greedy decoding ($\tau = 0$). We report results in Figure S3.

Figure S3: Benchmarking LLMs on the MedQA-USMLE and MedMCQA datasets using direct 0-shot and 5-shot CoT prompting. All results are obtained using greedy decoding ($\tau = 0$).

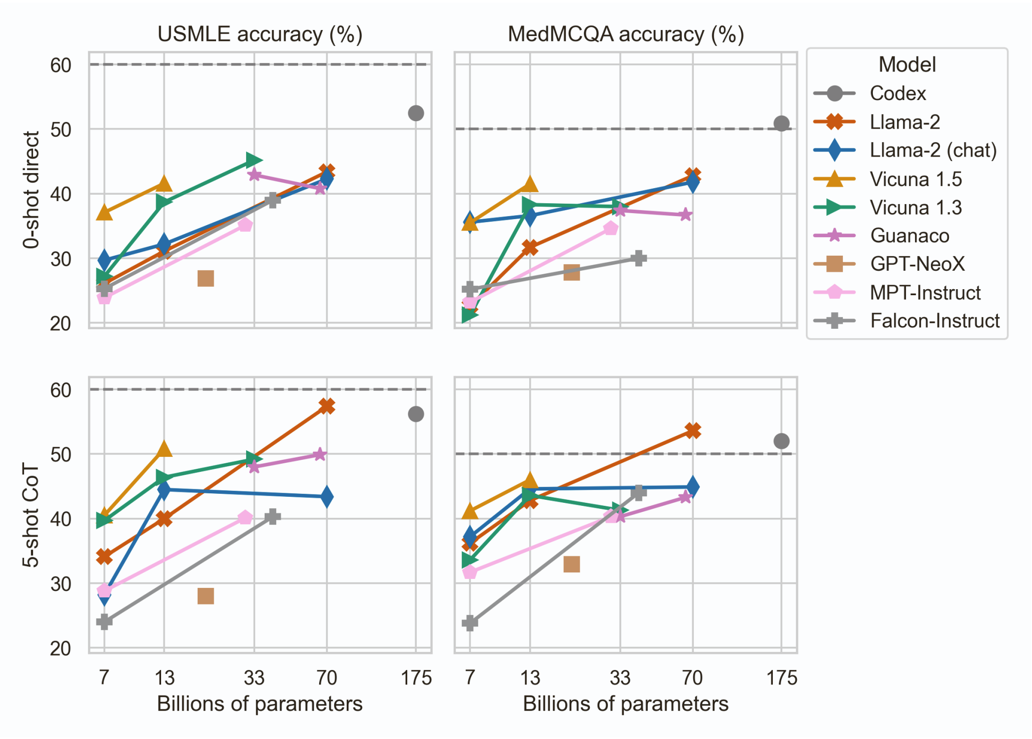

I Additional CoT Samples

CoT prompt variations In Figure S4, we report four selected CoTs generated from the prompt variations studied in Appendix B.

Codex CoTs In Figure S5, we display CoTs generated by Codex. Codex appears to yield CoTs of lower quality than InstructGPT (frequent repetitions, less verbosity).

Annotated InstructGPT CoTs We provided nine more expert-labelled chain-of-thoughts in Figures S6, S7, S8, S9, S10, S11, S12, S13 and S14. Note that patterns reported in Table 5 cannot always be matched to text segments, as one highlighted text segment does not always correspond to a single category (reasoning and knowledge patterns are often entangled).

Figure S4: A selection of remarkable CoTs generated by InstructGPT `text-davinci-002` based on USMLE questions. GPT-3 can adopt diverse problem-solving strategies.

Example 1. Applying Bayes rule

Parents bring an 11-month-old baby to the clinic because the baby has a fever of 39.0°C (102.2°F). The baby is irritated and crying constantly. She is up to date on immunizations. A complete physical examination reveals no significant findings, and all laboratory tests are negative. Five days after resolution of her fever, she develops a transient maculopapular rash. What is the most likely diagnosis?
A) Roseola B) Erythema infectiosum C) Rubella D) Kawasaki disease

✓ **Let's follow a Bayesian step by step approach**:
The prior probability of the child having each disease is:
Roseola: 1/4
Erythema infectiosum: 1/4
Rubella: 1/4
Kawasaki disease: 1/4

The likelihood of the child having each disease given the symptoms is:
Roseola: 3/4
Erythema infectiosum: 1/2
Rubella: 1/4
Kawasaki disease: 1/8

The posterior probability of the child having each disease is:
Roseola: 3/4 x 1/4 = 3/16
Erythema infectiosum: 1/2 x 1/4 = 1/8
Rubella: 1/4 x 1/4 = 1/16
Kawasaki disease: 1/8 x 1/4 = 1/32

The most likely diagnosis is Roseola.
Therefore, among A through D, the answer is A.

Example 2. Considering multiple hypotheses

A 6-year-old African-American boy presents with severe pain and swelling of both his hands and wrists. His symptoms onset 2 days ago and have not improved. He also has had diarrhea for the last 2 days and looks dehydrated. This patient has had two similar episodes of severe pain in the past. Physical examination reveals pallor, jaundice, dry mucous membranes, and sunken eyes. Which of the following mutations is most consistent with this patient's clinical condition?
A) Chromosomal deletion B) Nonsense **C) Missense** D) Frame shift

✓ **Let's derive the differential diagnosis step by step**:
1. Severe pain and swelling of hands and wrists: This can be caused by an inflammatory process (e.g. arthritis), an infection (e.g. septic arthritis), or trauma.

2. Diarrhea for 2 days: This can be caused by an infection (e.g. gastroenteritis), food poisoning, or a gastrointestinal disorder (e.g. inflammatory bowel disease).

3. Pallor, jaundice, dry mucous membranes, and sunken eyes: These are all signs of dehydration.

4. Two similar episodes of severe pain in the past: This suggests a chronic condition.

Based on the above information, the most likely diagnosis is a chronic inflammatory condition such as arthritis or inflammatory bowel disease. The mutation that is most consistent with this patient's clinical condition is a missense mutation.

Therefore, among A through D, the answer is C.

Example 3. Reflecting on each answer choice

A 12-month-old girl is brought in by her mother to the pediatrician for the first time since her 6-month checkup. The mother states that her daughter had been doing fine, but the parents are now concerned that their daughter is still not able to stand up or speak. On exam, the patient has a temperature of 98.5°F (36.9°C), pulse is 96/min, respirations are 20/min, and blood pressure is 100/80 mmHg. The child appears to have difficulty supporting herself while sitting. The patient has no other abnormal physical findings. She plays by herself and is making babbling noises but does not respond to her own name. She appears to have some purposeless motions. A previous clinic note documents typical development at her 6-month visit and mentioned that the patient was sitting unsupported at that time. Which of the following is the most likely diagnosis?
A) Language disorder **B) Rett syndrome** C) Fragile X syndrome D) Trisomy 21

✗ **Let's reflect on each answer option step by step**:
A) Language disorder: This cannot be the most likely diagnosis because the patient is only 12 months old. It is too early to diagnose a language disorder.

B) Rett syndrome: This is a possibility, but we cannot be certain because the patient does not have all the symptoms of Rett syndrome. The patient does have some purposeless motions, but does not have any of the other symptoms, such as loss of purposeful hand movements, deceleration of head growth, or problems with gait or movement.

C) Fragile X syndrome: This is also a possibility, but we cannot be certain because the patient does not have all the symptoms of Fragile X syndrome. The patient does have some purposeless motions, but does not have any of the other symptoms, such as macroorchidism, long face, or large ears.

D) Trisomy 21: This is the most likely diagnosis because the patient has some of the symptoms of Trisomy 21, such as a delay in milestones, difficulty supporting herself while sitting, and abnormal physical findings.

Therefore, among A through D, the answer is D.

Example 4. Working by elimination

A 35-year-old man presents with loose stools and left lower quadrant abdominal pain. He says he passes 8–10 loose stools per day. The volume of each bowel movement is small and appears mucoid with occasional blood. The patient reports a 20-pack-year smoking history. He also says he recently traveled abroad about 3 weeks ago to Egypt. The vital signs include: blood pressure 120/76 mm Hg, pulse 74/min, and temperature 36.5°C (97.8°F). On physical examination, mild to moderate tenderness to palpation in the left lower quadrant with no rebound or guarding is present. Rectal examination shows the presence of perianal skin ulcers. Which of the following is the most likely diagnosis in this patient?
A) Amebiasis B) Crohn's disease C) Salmonellosis D) Diverticulosis

✓ **Let's work by elimination step by step.**
The first thing to note is that the patient has perianal skin ulcers. This is not a symptom of Crohn's disease, so we can eliminate B.

The next thing to note is that the patient has recently traveled to Egypt. This increases the likelihood that the patient has amebiasis, so we can eliminate D.

The next thing to note is that the patient has a 20-pack-year smoking history. This increases the likelihood that the patient has diverticulosis, so we can eliminate C.

This leaves us with A, amebiasis, as the most likely diagnosis.

Therefore, among A through D, the answer is A.

Figure S5: Two randomly selected CoTs generated by Codex `code-davinci-002` based on USMLE questions. Codex tends to repeat itself and generate zero-shot CoTs of lower quality than InstructGPT.

A 24-year-old G2P1 woman at 39 weeks' gestation presents to the emergency department complaining of painful contractions occurring every 10 minutes for the past 2 hours, consistent with latent labor. She says she has not experienced vaginal discharge, bleeding, or fluid leakage, and is currently taking no medications. On physical examination, her blood pressure is 110/70 mm Hg, heart rate is 86/min, and temperature is 37.6°C (99.7°F). She has had little prenatal care and uses condoms inconsistently. Her sexually transmitted infections status is unknown. As part of the patient's workup, she undergoes a series of rapid screening tests that result in the administration of zidovudine during delivery. The infant is also given zidovudine to reduce the risk of transmission. A confirmatory test is then performed in the mother to confirm the diagnosis of HIV. Which of the following is most true about the confirmatory test?
A) It is a Southwestern blot, identifying the presence of DNA-binding proteins
B) It is a Northern blot, identifying the presence of RNA
C) It is a Northern blot, identifying the presence of DNA
D) It is an HIV-1/HIV2 antibody differentiation immunoassay

✓ **Let's think step by step**.
The patient is in latent labor. She has had little prenatal care and uses condoms inconsistently. Her sexually transmitted infections status is unknown. As part of the patient's workup, she undergoes a series of rapid screening tests that result in the administration of zidovudine during delivery. The infant is also given zidovudine to reduce the risk of transmission. A confirmatory test is then performed in the mother to confirm the diagnosis of HIV.
The patient is in latent labor. This means that she is in the first stage of labor. The first stage of labor is divided into two phases: the latent phase and the active phase. The latent phase is the longest phase of labor. It is characterized by regular contractions that are not yet strong enough to dilate the cervix. The latent phase can last for hours or even days. The active phase is characterized by stronger contractions that dilate the cervix. The active phase is shorter than the latent phase.
She has had little prenatal care and uses condoms inconsistently. This means that she is at risk for HIV.
Her sexually transmitted infections status is unknown. This means that she is at risk for HIV.
As part of the patient's workup, she undergoes a series of rapid screening tests that result in the administration of zidovudine during delivery. This means that she is at risk for HIV.
The infant is also given zidovudine to reduce the risk of transmission. This means that she is at risk for HIV.
A confirmatory test is then performed in the mother to confirm the diagnosis of HIV. This means that she is at risk for HIV.
The patient is in latent labor. This means that <This is not generated by GPT-3: The above paragraph was repeated a few more times>
Therefore, among A through D, the answer is D.

A 51-year-old man is brought to the emergency department because of a 2-day history of fever, abdominal pain, and confusion. His wife states that he has been unable to recall his birthday or her name. He was diagnosed with hepatitis C 3 years ago but refused treatment. He has been treated twice in the past year for acute pancreatitis. There is no family history of serious illness. His only medication is a calcium supplement. He emigrated from India 15 years ago. He appears ill. His temperature is 38.3°C (100.9°F), pulse is 101/min, and blood pressure is 104/68 mm Hg. He is confused and oriented only to person. Examination shows scleral icterus and spider angiomas. There are fine tremors of the hands bilaterally. The abdomen is distended and shifting dullness is present. There is diffuse tenderness to palpation with no guarding. Bowel sounds are absent. Laboratory studies show: Hemoglobin 12.6 g/dL Leukocyte count 13,900/mm3 Platelet count 342,000/mm3 Serum Albumin 2.6 g/dL Total bilirubin 2.56 mg/dL Alkaline phosphatase 54 U/L AST 17 U/L ALT 44 U/L Paracentesis is performed. Ascitic fluid analysis shows an albumin concentration of 0.8 g/dL, glucose concentration of 62 mg/dL, and a leukocyte count of 1900/mm3 with 60% neutrophils. Which of the following is the most likely explanation for these findings?"
A) Aseptic peritoneal inflammation
B) Neoplastic growth
C) Bacterial translocation
D) Perforated viscus

✗ **Let's think step by step**
Hepatitis C is a viral infection that causes inflammation of the liver.
He has a history of acute pancreatitis.
Acute pancreatitis is inflammation of the pancreas.
He has a history of ascites.
Ascites is the accumulation of fluid in the peritoneal cavity.
He has a history of confusion.
Confusion is a symptom of hepatic encephalopathy.
Hepatic encephalopathy is a complication of liver disease.
The patient has a history of hepatitis C.
Hepatitis C is a viral infection that causes inflammation of the liver.
He has a history of acute pancreatitis.
Acute pancreatitis is inflammation of the pancreas.
He has a history of ascites.
Ascites is the accumulation of fluid in the peritoneal cavity.
He has a history of confusion.
Confusion is a symptom of hepatic encephalopathy.
Hepatic encephalopathy is a complication of liver disease.
The patient has a history of hepatitis C.
Hepatitis C is a viral infection that causes inflammation of the liver.
He has a history of acute pancreatitis.
<This is not generated by GPT-3: The above paragraph was repeated a few more times>
Therefore, among A through D, the answer is D.

Figure S6: (Sample 2) Generated zero-shot Chain-of-Thought from InstructGPT `text-davinci-002` for three CoT prompts on a sample for the MedQA-USMLE test set.

USMLE [test:1050] A 40-year-old female with a past medical history of high cholesterol, high blood pressure, hyperthyroidism, and asthma presents to the primary care clinic today. She has tried several different statins, all of which have resulted in bothersome side effects. Her current medications include hydrochlorothiazide, levothyroxine, albuterol, oral contraceptives, and a multivitamin. Her physical examination is unremarkable. Her blood pressure is 116/82 mm Hg and her heart rate is 82/min. You decide to initiate colesevelam (Welchol). Of the following, which is a concern with the initiation of this medication?
A) Colesevelam can cause cognitive impairment.
B) Colesevelam can increase the risk of cholelithiasis.
C) Timing of the dosing of colesevelam should be separated from this patient's other medications.
D) Colesevelam can significantly decrease your HDL.

✓ Let's think step by step. The patient has tried several different statins, all of which have resulted in bothersome side effects. The patient's current medications include hydrochlorothiazide, levothyroxine, albuterol, oral contraceptives, and a multivitamin. The physical examination is unremarkable. The blood pressure is 116/82 mm Hg and the heart rate is 82/min. You decide to initiate colesevelam (Welchol). The concern with the initiation of this medication is that timing of the dosing of colesevelam should be separated from this patient's other medications.
Therefore, among A through D, the answer is C.

✗ Let's think step by step like a medical expert. The first thing to consider is the patient's current medications. The patient is currently taking hydrochlorothiazide, levothyroxine, albuterol, oral contraceptives, and a multivitamin. The next thing to consider is the patient's medical history. The patient has a history of high cholesterol, high blood pressure, hyperthyroidism, and asthma. The third thing to consider is the patient's physical examination. The patient's physical examination is unremarkable. The fourth thing to consider is the patient's blood pressure and heart rate. The patient's blood pressure is 116/82 mm Hg and her heart rate is 82/min. The fifth and final thing to consider is the medication that the doctor is considering prescribing, colesevelam (Welchol). The concerns with the initiation of this medication are that it can cause cognitive impairment, it can increase the risk of cholelithiasis, timing of the dosing of colesevelam should be separated from this patient's other medications, and it can significantly decrease your HDL.
Therefore, among A through D, the answer is D.

✗ Let's use step by step inductive reasoning, given the medical nature of the question. First, we know that the patient has high cholesterol, which is the main reason why the physician would prescribe colesevelam. Second, we know that the patient has tried several different statins, all of which have resulted in bothersome side effects. Third, we know that colesevelam can cause cognitive impairment. Fourth, we know that colesevelam can increase the risk of cholelithiasis. Therefore, the correct answer is D.)
Therefore, among A through D, the answer is D.

Pattern A	Correct reasoning step	Pattern D	Incorrect reasoning step
Pattern B	Correct recall of knowledge	Pattern E	Incorrect or insufficient knowledge
Pattern C	Correct reading comprehension	Pattern F	Incorrect reading comprehension

Figure S7: (Sample 3) Generated zero-shot Chain-of-Thought from InstructGPT `text-davinci-002` for three CoT prompts on a sample for the MedQA-USMLE test set.

USMLE [test:805] A 79-year-old woman comes to the physician because of a 1-month history of difficulty starting urination and a vague sensation of fullness in the pelvis. Pelvic speculum examination in the lithotomy position shows a pink structure at the vaginal introitus that protrudes from the anterior vaginal wall when the patient is asked to cough. Which of the following is the most likely cause of this patient's symptoms?
A) Vaginal rhabdomyosarcoma **B) Cystocele** C) Rectocele D) Uterine leiomyomata

✓ **Let's use step by step inductive reasoning, given the medical nature of the question.** The first thing to note is that the patient is an elderly woman. This means that we can rule out rhabdomyosarcoma, which is a type of cancer that is most commonly found in children.

The next thing to note is that the patient is experiencing difficulty starting urination and a vague sensation of fullness in the pelvis. This could be caused by a number of things, but the most likely cause is a cystocele. A cystocele is a condition in which the bladder bulges into the vagina. This can cause difficulty starting urination because the bladder is not able to empty properly.

The final thing to note is that the pelvic examination shows a pink structure at the vaginal introitus that protrudes from the anterior vaginal wall when the patient is asked to cough. This is most likely a rectocele. A rectocele is a condition in which the rectum bulges into the vagina. This can cause the vague sensation of fullness in the pelvis that the patient is experiencing.

So, in conclusion, the most likely cause of the patient's symptoms is a cystocele.

Therefore, among A through D, the answer is B.

✗ **Let's differentiate using step by step reasoning like a medical expert**:
Differential diagnosis for a pink structure at the vaginal introitus that protrudes from the anterior vaginal wall when the patient is asked to cough:

1) Vaginal rhabdomyosarcoma: This is a very unlikely diagnosis given the patient's age and the lack of other symptoms.

2) Cystocele: This is a possible diagnosis, but is more likely to present with urinary incontinence rather than difficulty starting urination.

3) Rectocele: This is also a possible diagnosis, but is more likely to present with constipation or difficulty having a bowel movement.

4) Uterine leiomyomata: This is the most likely diagnosis given the patient's age and symptoms.

Therefore, among A through D, the answer is D.

✗ **Let's derive the differential diagnosis step by step**.
The first step is to identify the relevant anatomy. The relevant anatomy here is the pelvic floor, which includes the vagina, uterus, bladder, and rectum.

The second step is to identify the relevant symptoms. The relevant symptoms here are difficulty starting urination and a vague sensation of fullness in the pelvis.

The third step is to identify the possible causes of the symptoms. The possible causes of the symptoms could be a mass in the pelvis, an obstruction in the urinary tract, or a problem with the pelvic floor muscles.

The fourth step is to narrow down the differential diagnosis. The most likely cause of the patient's symptoms is a mass in the pelvis. The mass could be a cystocele, rectocele, or uterine leiomyomata.

Therefore, among A through D, the answer is D.

Pattern A	Correct reasoning step	Pattern D	Incorrect reasoning step
Pattern B	Correct recall of knowledge	Pattern E	Incorrect or insufficient knowledge
Pattern C	Correct reading comprehension	Pattern F	Incorrect reading comprehension

Figure S8: (Sample 4) Generated zero-shot Chain-of-Thought from InstructGPT `text-davinci-002` for three CoT prompts on a sample for the MedQA-USMLE test set.

USMLE [test:951] A 22-year-old woman comes to the physician for a routine health examination. She feels well but asks for advice about smoking cessation. She has smoked one pack of cigarettes daily for 7 years. She has tried to quit several times without success. During the previous attempts, she has been extremely nervous and also gained weight. She has also tried nicotine lozenges but stopped taking them because of severe headaches and insomnia. She has bulimia nervosa. She takes no medications. She is 168 cm (5 ft 6 in) tall and weighs 68 kg (150 lb); BMI is 24 kg/m2. Physical and neurologic examinations show no other abnormalities. Which of the following is the most appropriate next step in management?
A) Diazepam B) Nicotine patch **C) Varenicline** D) Motivational interviewing

✓ **Let's think step by step.** The first thing we need to do is assess whether this patient is ready to quit smoking. This can be done using the 5 A's:
1. Ask the patient if they smoke
2. Advise the patient to quit
3. Assess the patient's willingness to quit
4. Assist the patient in quitting
5. Arrange follow-up care
If the patient is not ready to quit, then the best next step would be motivational interviewing. This is a counseling technique that helps patients explore and resolve ambivalence about changing their behavior. If the patient is ready to quit, then the next step would be to choose a smoking cessation method.
The best method for this patient would be varenicline. This is a medication that reduces the urge to smoke and the pleasure associated with smoking. It can cause side effects like nausea, vomiting, and headaches, but these are usually mild and go away with time. **Therefore, among A through D, the answer is C.**

✗ **Let's think step by step like a medical expert.** The first thing to consider is the patient's history of bulimia nervosa. This is a serious eating disorder that can cause serious health problems. Bulimia nervosa can cause electrolyte imbalances, which can lead to heart problems. It can also cause dehydration, which can lead to kidney problems. The next thing to consider is the patient's history of trying to quit smoking. The patient has tried to quit several times without success. During the previous attempts, she has been extremely nervous and also gained weight. She has also tried nicotine lozenges but stopped taking them because of severe headaches and insomnia. Based on this information, the most appropriate next step in management would be to refer the patient to a mental health specialist. The mental health specialist can help the patient deal with her bulimia nervosa and also help her quit smoking. **Therefore, among A through D, the answer is D.**

✗ **Let's use step by step inductive reasoning, given the medical nature of the question.** The first thing we need to do is look at the options given and see if any can be immediately eliminated. We can see that option A, diazepam, is a medication, so it can be eliminated. We are looking for the most appropriate next step, not a medication. Option B, the nicotine patch, is a possibility. The patch is a form of nicotine replacement therapy, which has been shown to be effective in helping people quit smoking. Option C, varenicline, is also a possibility. Varenicline is a medication that helps to reduce the urge to smoke. Option D, motivational interviewing, is a possibility. Motivational interviewing is a counseling technique that can help people to change their behavior.
So, which of these is the most appropriate next step in management? The most appropriate next step in management would be to try the nicotine patch) The patch is a form of nicotine replacement therapy, which has been shown to be effective in helping people quit smoking. **Therefore, among A through D, the answer is B.**

Pattern A	Correct reasoning step	Pattern D	Incorrect reasoning step
Pattern B	Correct recall of knowledge	Pattern E	Incorrect or insufficient knowledge
Pattern C	Correct reading comprehension	Pattern F	Incorrect reading comprehension

Figure S9: (Sample 5) Generated zero-shot Chain-of-Thought from InstructGPT `text-davinci-002` for three CoT prompts on a sample for the MedQA-USMLE test set.

USMLE [test:201] A 17-year-old girl comes to the physician because of an 8-month history of severe acne vulgaris over her face, upper back, arms, and buttocks. Treatment with oral antibiotics and topical combination therapy with benzoyl peroxide and retinoid has not completely resolved her symptoms. Examination shows oily skin with numerous comedones, pustules, and scarring over the face and upper back. Long-term therapy is started with combined oral contraceptive pills. This medication decreases the patient's risk developing of which of the following conditions?
A) Hypertension **B) Ovarian cancer** C) Cervical cancer D) Breast cancer

✗ **Let's think step by step like a medical expert.** The first thing to consider is the patient's age. The patient is 17 years old, which means she is of reproductive age.
The next thing to consider is the patient's symptoms. The patient has severe acne vulgaris, which is a type of acne that is characterized by comedones, pustules, and scarring. The next thing to consider is the patient's treatment. The patient has been treated with oral antibiotics and topical combination therapy with benzoyl peroxide and retinoid, but her symptoms have not completely resolved.
Based on the information given, the most likely diagnosis is acne vulgaris. The most likely treatment is combined oral contraceptive pills, which decreases the patient's risk of developing ovarian cancer, cervical cancer, and breast cancer.
Therefore, among A through D, the answer is D, breast cancer.

✗ **Let's differentiate using step by step reasoning like a medical expert:**
A) Hypertension: This is not a common side effect of combined oral contraceptive pills.

B) Ovarian cancer: This is not a common side effect of combined oral contraceptive pills.

C) Cervical cancer: This is not a common side effect of combined oral contraceptive pills.

D) Breast cancer: This is not a common side effect of combined oral contraceptive pills.
Therefore, among A through D, the answer is none of the above.

✗ **Let's derive the differential diagnosis step by step:** The patient has severe acne vulgaris. Treatment with oral antibiotics and topical combination therapy with benzoyl peroxide and retinoid has not completely resolved her symptoms.
Differential diagnosis:
1) Hormonal imbalance: This could be due to an imbalance in androgen levels, which can lead to increased sebum production and thus acne.
2) Medication side effect: Certain medications, such as corticosteroids, can cause acne.
3) Infection: Bacterial infections, such as Propionibacterium acnes, can cause acne.
4) Genetics: Acne can be hereditary. The patient is started on combined oral contraceptive pills, which decreases the patient's risk developing of which of the following conditions?
A) Hypertension B) Ovarian cancer C) Cervical cancer D) Breast cancer
The correct answer is D) Breast cancer.
Therefore, among A through D, the answer is D.

Pattern A	Correct reasoning step	Pattern D	Incorrect reasoning step
Pattern B	Correct recall of knowledge	Pattern E	Incorrect or insufficient knowledge
Pattern C	Correct reading comprehension	Pattern F	Incorrect reading comprehension

Figure S10: (Sample 6) Generated zero-shot Chain-of-Thought from InstructGPT `text-davinci-002` for three CoT prompts on a sample for the MedQA-USMLE test set.

USMLE [test:790] A 56-year-old patient is being treated with oral amoxicillin for community-acquired pneumonia. The plasma clearance of the drug is calculated as 15.0 L/h. Oral bioavailability of the drug is 75%. Sensitivity analysis of a sputum culture shows a minimal inhibitory concentration of 1 μg/mL for the causative pathogen. The target plasma concentration is 2 mg/L. If the drug is administered twice per day, which of the following dosages should be administered at each dosing interval to maintain a steady state?
A) 270 mg **B) 480 mg** C) 240 mg D) 540 mg

✗ Let's think step by step. The first thing we need to do is calculate the patient's daily dose. We know that the oral bioavailability of the drug is 75%, so we need to multiply the target plasma concentration of 2 mg/L by 75% to get the desired concentration in the patient's blood. This comes out to 1.5 mg/L.
Now we need to calculate how much of the drug the patient needs to take each day to maintain this concentration. We know that the plasma clearance of the drug is 15.0 L/h, so the patient will need to take 15.0 L/h / 24 h = 0.625 L/day of the drug. Now we need to convert this into mg/day. We know that 1 mg/L = 1000 μg/L, so 1.5 mg/L = 1500 μg/L. This means that the patient will need to take 1500 μg/day of the drug to maintain a steady state.
Now we need to calculate the dosage for each dosing interval. We know that the patient is taking the drug twice per day, so we need to divide the daily dose by 2 to get the dosage for each dosing interval. This comes out to 750 μg/dose.
Now we need to convert this into mg/dose. We know that 1 mg = 1000 μg, so 750 μg/dose = 0.75 mg/dose.
The patient should be taking 0.75 mg of the drug at each dosing interval.
Therefore, among A through D, the answer is C.

✗ Let's think step by step like a medical expert.
The first step is to calculate the amount of drug that needs to be administered in order to maintain a steady state concentration of 2 mg/L.
The equation for this is:
(Concentration)(Clearance) = (Dose)(Bioavailability) We know that the concentration is 2 mg/L, the clearance is 15 L/hr, and the bioavailability is 75%. This leaves us with the equation:
(2 mg/L)(15 L/hr) = (Dose)(0.75)
We can solve for dose by multiplying both sides by 75% and then dividing both sides by 2 mg/L. This gives us:
(2 mg/L)(15 L/hr)(0.75) = (Dose)(0.75)(0.75)
(2 mg/L)(15 L/hr)(0.75) = (Dose)(0.5625)
Dose = (2 mg/L)(15 L/hr)(0.75) / (0.5625)
Dose = (30 mg/hr) / (0.5625)
Dose = 53.33 mg/hr
Now that we know the amount of drug that needs to be administered per hour, we can calculate the amount of drug that needs to be administered at each dosing interval.
Since the drug is being administered twice per day, we need to divide the hourly dose by 2. This gives us the equation:
(53.33 mg/hr) / (2) = (Dose at each dosing interval)
(53.33 mg/hr) / (2) = 26.67 mg
The answer is C) 240 mg
Therefore, among A through D, the answer is C) 240 mg.

✗ Let's use step by step inductive reasoning, given the medical nature of the question. 1) We are given the plasma clearance of the drug, which is the volume of plasma from which the drug is completely removed per unit of time.
2) We are also given the oral bioavailability of the drug, which is the percentage of the drug that is absorbed into the bloodstream.
3) We know that the target plasma concentration is 2 mg/L.
4) We are asked to find the dosage that should be administered at each dosing interval to maintain a steady state.
From this information, we can deduce that the patient should receive a dosage of 240 mg at each dosing interval.
Therefore, among A through D, the answer is C.

Pattern A	Correct reasoning step	Pattern D	Incorrect reasoning step
Pattern B	Correct recall of knowledge	Pattern E	Incorrect or insufficient knowledge
Pattern C	Correct reading comprehension	Pattern F	Incorrect reading comprehension

Figure S11: (Sample 7) Generated zero-shot Chain-of-Thought from InstructGPT `text-davinci-002` for three CoT prompts on a sample for the MedQA-USMLE test set.

USMLE [test:629] A 16-year-old boy is brought to the emergency department by ambulance from a soccer game. During the game, he was about to kick the ball when another player collided with his leg from the front. He was unable to stand up after this collision and reported severe knee pain. On presentation, he was found to have a mild knee effusion. Physical exam showed that his knee could be pushed posteriorly at 90 degrees of flexion but it could not be pulled anteriorly in the same position. The anatomic structure that was most likely injured in this patient has which of the following characteristics?
A) Runs anteriorly from the medial femoral condyle B) Runs medially from the lateral femoral condyle C) Runs posteriorly from the lateral femoral condyle **D) Runs posteriorly from the medial femoral condyle**

✓ Let's think step by step like a medical expert.
1) What is the most likely diagnosis? The most likely diagnosis is a medial collateral ligament (MCL) tear. 2) What are the characteristics of an MCL tear? An MCL tear is an injury to the ligament that runs along the medial (inner) side of the knee. The MCL helps to stabilize the knee joint. 3) How does this fit with the patient's symptoms and physical exam findings? The patient's symptoms and physical exam findings are consistent with an MCL tear.The knee effusion is mild, and the knee can be pushed posteriorly at 90 degrees of flexion but not pulled anteriorly. This is consistent with an MCL tear.
Therefore, among A through D, the answer is D.

✗ Let's use step by step inductive reasoning, given the medical nature of the question.
The first thing to note is that the patient has a mild knee effusion, meaning there is some fluid in the knee joint. This is likely due to injury.
Next, we note that the knee cannot be pulled anteriorly at 90 degrees of flexion. This suggests that the anterior cruciate ligament (ACL) may be injured. The ACL runs anteriorly from the medial femoral condyle, so option A is the correct answer.
Therefore, among A through D, the answer is A.

✗ Let's differentiate using step by step reasoning like a medical expert:
1) The patient has a mild knee effusion, meaning there is some fluid in the knee joint. This is likely due to injury.
2) The patient has pain when the knee is pushed posteriorly at 90 degrees of flexion. This suggests that the posterior cruciate ligament (PCL) is injured.
3) The patient cannot pull the knee anteriorly in the same position. This suggests that the anterior cruciate ligament (ACL) is also injured. 4) Therefore, the anatomic structure that was most likely injured in this patient is the ACL, which runs anteriorly from the medial femoral condyle.
Therefore, among A through D, the answer is A.

Pattern A	Correct reasoning step	Pattern D	Incorrect reasoning step
Pattern B	Correct recall of knowledge	Pattern E	Incorrect or insufficient knowledge
Pattern C	Correct reading comprehension	Pattern F	Incorrect reading comprehension

Figure S12: (Sample 8) Generated zero-shot Chain-of-Thought from InstructGPT `text-davinci-002` for three CoT prompts on a sample for the MedQA-USMLE test set.

USMLE [test:757] An 18-year-old woman is brought to the emergency department because of lightheadedness and a feeling of dizziness. She has had nausea, occasional episodes of vomiting, myalgia, and a generalized rash for the past week. She also reports feeling lethargic. She has no shortness of breath. There is no family history of serious illness. She appears ill. Her temperature is 39.1°C (102.3°F), pulse is 118/min, and blood pressure is 94/60 mm Hg. Cardiac examination shows no abnormalities. There is a widespread erythematous rash on the trunk and extremities with skin peeling on the palms and soles. Laboratory studies show:
Hemoglobin 13.6 g/dL Leukocyte count 19,300/mm3 Platelet count 98,000/mm3
Serum Urea nitrogen 47 mg/dL Glucose 88 mg/dL Creatinine 1.8 mg/dL
Total bilirubin 2.1 mg/dL AST 190 U/L ALT 175 U/L
Urinalysis shows no abnormalities. Further evaluation of this patient's history is most likely to reveal which of the following?
A) Recent hiking trip B) Intravenous heroin abuse C) Exposure to a patient with mengingococcemia **D) Currently menstruating**

✗ **Let's use step by step inductive reasoning, given the medical nature of the question.** The patient is 18 years old, female, and has a generalized rash, lightheadedness, feeling of dizziness, nausea, myalgia, and occasional vomiting. She also reports feeling lethargic.
The patient's laboratory studies show:
Hemoglobin 13.6 g/dL Leukocyte count 19,300/mm3 Platelet count 98,000/mm3

Serum Urea nitrogen 47 mg/dL Glucose 88 mg/dL Creatinine 1.8 mg/dL
Total bilirubin 2.1 mg/dL AST 190 U/L ALT 175 U/L
Urinalysis shows no abnormalities. The most likely explanation for this patient's history is exposure to a patient with meningococcemia.
Therefore, among A through D, the answer is C.

✗ **Let's differentiate using step by step reasoning like a medical expert:** The patient has a widespread erythematous rash on the trunk and extremities with skin peeling on the palms and soles. This suggests a possible diagnosis of erythema multiforme, which is most commonly caused by a viral infection.
The patient has a history of lightheadedness, dizziness, nausea, vomiting, myalgia, and a generalized rash. These are all symptoms of meningitis, which is a serious infection of the membranes that cover the brain and spinal cord.
The patient has a history of lightheadedness, dizziness, nausea, vomiting, myalgia, and a generalized rash. These are all symptoms of meningitis, which is a serious infection of the membranes that cover the brain and spinal cord.
The patient has a history of lightheadedness, dizziness, nausea, vomiting, myalgia, and a generalized rash. These are all symptoms of meningitis, which is a serious infection of the membranes that cover the brain and spinal cord.
The patient has a history of lightheadedness, dizziness, nausea, vomiting, myalgia, and a generalized rash. These are all symptoms of meningitis, which is a serious infection of the membranes that cover the brain and spinal cord.
`<This is not generated by GPT-3: The above paragraph was repeated 15 more times>`
Therefore, among A through D, the answer is C) Exposure to a patient with meningococcemia.

✗ **Let's derive the differential diagnosis step by step**: The patient has lightheadedness, dizziness, nausea, vomiting, myalgia, and a generalized rash. She also reports feeling lethargic. She has no shortness of breath. There is no family history of serious illness. She appears ill.
Differential diagnosis for these symptoms includes: 1) Infectious diseases (e.g. meningitis, meningococcemia, influenza, viral gastroenteritis) 2) Autoimmune diseases (e.g. lupus, rheumatoid arthritis) 3) Neurological disorders (e.g. migraine, vertigo) 4) Gastrointestinal disorders (e.g. food poisoning, gastroenteritis) 5) Endocrine disorders (e.g. Addison's disease, hypothyroidism)
The patient's temperature is 39.1°C (102.3°F), pulse is 118/min, and blood pressure is 94/60 mm Hg. Cardiac examination shows no abnormalities. There is a widespread erythematous rash on the trunk and extremities with skin peeling on the palms and soles. Laboratory studies show:
Hemoglobin 13.6 g/dL Leukocyte count 19,300/mm3 Platelet count 98,000/mm3

Serum Urea nitrogen 47 mg/dL Glucose 88 mg/dL Creatinine 1.8 mg/dL Total bilirubin 2.1 mg/dL AST 190 U/L ALT 175 U/L
Urinalysis shows no abnormalities. Differential diagnosis for these findings includes: 1) Infectious diseases (e.g. meningococcemia, sepsis) 2) Autoimmune diseases (e.g. lupus, rheumatoid arthritis) 3) Hematologic disorders (e.g. leukemia, thrombocytopenic purpura) 4) Liver disorders (e.g. hepatitis, fatty liver disease) 5) renal failure
Further evaluation of this patient's history is most likely to reveal which of the following?
The most likely answer is C) Exposure to a patient with meningococcemia. This patient presents with a clinical picture and laboratory findings consistent with meningococcemia, a potentially life-threatening infection caused by the bacteria Neisseria meningitidis.
Therefore, among A through D, the answer is C.

Pattern A	Correct reasoning step	Pattern D	Incorrect reasoning step
Pattern B	Correct recall of knowledge	Pattern E	Incorrect or insufficient knowledge
Pattern C	Correct reading comprehension	Pattern F	Incorrect reading comprehension

Figure S13: (Sample 9) Generated zero-shot Chain-of-Thought from InstructGPT `text-davinci-002` for three CoT prompts on a sample for the MedQA-USMLE test set.

USMLE [test:286] A 27-year-old HIV positive female gave birth to a 7lb 2oz (3.2 kg) baby girl. The obstetrician is worried that the child may have been infected due to the mother's haphazard use of her anti-retroviral medications. Which of the following assays would a hospital use detect the genetic material of HIV if the child had been infected?
A) Enzyme-linked immunosorbent assay (ELISA) B) Rapid HIV antibody test **C) Polymerase chain reaction** D) Southern blot

✓ Let's use step by step inductive reasoning, given the medical nature of the question.

The first step is to understand the question. In this case, the question is asking which of the following assays would a hospital use to detect the genetic material of HIV if the child had been infected.
The second step is to understand the options. In this case, the options are enzyme-linked immunosorbent assay (ELISA), rapid HIV antibody test, polymerase chain reaction, and Southern blot.
The third step is to understand what each option is. Enzyme-linked immunosorbent assay (ELISA) is a test that is used to detect antibodies in the blood. Rapid HIV antibody test is a test that is used to detect HIV antibodies in the blood. Polymerase chain reaction is a test that is used to amplify DNA. Southern blot is a test that is used to detect DNA.
The fourth step is to understand what the question is asking. In this case, the question is asking which of the following assays would a hospital use to detect the genetic material of HIV if the child had been infected.
The fifth step is to understand what each option would do. Enzyme-linked immunosorbent assay (ELISA) would not detect the genetic material of HIV if the child had been infected. Rapid HIV antibody test would not detect the genetic material of HIV if the child had been infected. Polymerase chain reaction would detect the genetic material of HIV if the child had been infected. Southern blot would detect the genetic material of HIV if the child had been infected.
The sixth and final step is to choose the best option. In this case, the best option is polymerase chain reaction.
Therefore, among A through D, the answer is C.

✓ Let's differentiate using step by step reasoning like a medical expert.

The first step is to understand what the question is asking. In this case, the question is asking which assay would a hospital use to detect the genetic material of HIV if the child had been infected.
The second step is to understand what each option is.
Option A is the ELISA test, which is a test that looks for antibodies in the blood.
Option B is the Rapid HIV antibody test, which is a test that looks for antibodies in the blood and can give results in as little as 20 minutes.
Option C is the Polymerase Chain Reaction test, which is a test that looks for the genetic material of the virus in the blood.
Option D is the Southern blot test, which is a test that looks for the genetic material of the virus in the blood and can be used to confirm the results of the PCR test.
The third step is to understand what we are looking for. In this case, we are looking for the genetic material of HIV.
The fourth step is to eliminate the options that are not related to what we are looking for. In this case, we are looking for the genetic material of HIV, so we can eliminate options A and B because they are both tests that look for antibodies in the blood.

This leaves us with options C and D.
The fifth step is to eliminate the option that is not the best answer. In this case, option C is the best answer because it is a test that looks for the genetic material of the virus in the blood.
Therefore, among A through D, the answer is C.

✓ Let's derive the differential diagnosis step by step.

The first step is to consider if the child was infected in utero, during delivery, or postnatally. If the child was infected in utero or during delivery, then the child's blood would test positive for HIV RNA. If the child was infected postnatally, then the child's blood would test positive for HIV antibodies.
The second step is to consider which test would be most appropriate to confirm the diagnosis. ELISA and rapid HIV antibody tests are used to detect HIV antibodies. Polymerase chain reaction is used to detect HIV RNA. Southern blot is used to detect HIV DNA.
In this case, the most appropriate test to confirm the diagnosis would be polymerase chain reaction, since the child was likely infected in utero or during delivery.
Therefore, among A through D, the answer is C.

Pattern A	Correct reasoning step	Pattern D	Incorrect reasoning step
Pattern B	Correct recall of knowledge	Pattern E	Incorrect or insufficient knowledge
Pattern C	Correct reading comprehension	Pattern F	Incorrect reading comprehension

Figure S14: (Sample 10) Generated zero-shot Chain-of-Thought from InstructGPT `text-davinci-002` for three CoT prompts on a sample for the MedQA-USMLE test set.

USMLE [test:714] A 28-year-old male presents to the emergency department with chest pain. He reports that one hour ago he was climbing the stairs in his home when he suddenly developed excruciating chest pain. He describes the pain as ripping and radiating to the back. The patient's past medical history is significant for a heart murmur that is being followed by his outpatient cardiologist. He takes no medications. The patient works as a carpenter and reports being monogamous with his wife. He denies any history of intravenous drug use or recent travel. In the emergency department, the patient's temperature is 99.4°F (37.4°C), blood pressure is 94/56 mmHg, pulse is 121/min, and respirations are 14/min. On physical exam, the patient appears to be in moderate distress and is clutching his chest. He is tall and has joint hypermobility. His breath sounds are equal and full bilaterally and has weak distal pulses.
Which of the following is most likely to be found in this patient?
A) Holosystolic, high-pitched blowing murmur at the lower left sternal border **B) Late systolic crescendo murmur at the apex with mid-systolic click** C) Mid-diastolic murmur at the left lower sternal border D) Systolic ejection murmur at the right second intercostal space

✗ **Let's think step by step.** The patient has chest pain, so we're thinking about the heart. The patient has a heart murmur, so we're thinking about valvular disease. The patient is tall and has joint hypermobility, so we're thinking about Marfan syndrome. The patient has weak distal pulses, so we're thinking about aortic dissection.<br>The most likely diagnosis is aortic dissection. Aortic dissection is a tear in the aorta that can cause chest pain. Aortic dissection can be caused by Marfan syndrome, which is a condition that affects the connective tissue. Marfan syndrome can cause joint hypermobility and aortic aneurysms.<br>**Therefore, among A through D, the answer is D, systolic ejection murmur at the right second intercostal space.**	✗ **Let's think step by step like a medical expert.**<br>The first thing to consider is the patient's symptoms. The patient has chest pain that is described as "ripping" and radiating to the back.This suggests that the pain is originating from the heart.<br>The second thing to consider is the patient's past medical history. The patient has a heart murmur that is being followed by an outpatient cardiologist. This suggests that the patient may have a heart condition that is causing the chest pain.<br>The third thing to consider is the patient's medications. The patient is not taking any medications. This suggests that the chest pain is not being caused by a medication side effect.<br>The fourth thing to consider is the patient's occupation. The patient is a carpenter. This suggests that the chest pain may be caused by physical activity.<br>The fifth thing to consider is the patient's travel history. The patient denies any recent travel. This suggests that the chest pain is not being caused by travel.<br>The sixth thing to consider is the patient's intravenous drug use history. The patient denies any history of intravenous drug use. This suggests that the chest pain is not being caused by drug use.<br>The seventh thing to consider is the patient's physical examination. The patient appears to be in moderate distress and is clutching his chest. He has weak distal pulses. This suggests that the chest pain is causing the patient to feel distressed and that the patient's heart may not be pumping blood effectively.<br>Based on the above information, the most likely cause of the patient's chest pain is a systolic ejection murmur at the right second intercostal space.<br>**Therefore, among A through D, the answer is D.**	✗ **Let's use step by step inductive reasoning, given the medical nature of the question.** The first thing to note is that the patient has a heart murmur. This is significant because it means that the patient is at risk for developing heart problems.<br>The next thing to note is that the patient is experiencing chest pain. This is significant because it is a symptom of a heart attack.<br>The third thing to note is that the patient is tall and has joint hypermobility. This is significant because it means that the patient is at risk for developing aortic dissection.<br>Based on the information given, the most likely diagnosis is aortic dissection.<br>**Therefore, among A through D, the answer is D.**

Pattern A	Correct reasoning step	Pattern D	Incorrect reasoning step
Pattern B	Correct recall of knowledge	Pattern E	Incorrect or insufficient knowledge
Pattern C	Correct reading comprehension	Pattern F	Incorrect reading comprehension

References

S1 Robinson, J., Rytting, C.M., Wingate, D.. Leveraging Large Language Models for Multiple Choice Question Answering. Preprint at arXiv 2022;https://doi.org/10.48550/arXiv.2210.12353.

S2 Nori, H., King, N., McKinney, S.M., Carignan, D., Horvitz, E.. Capabilities of GPT-4 on Medical Challenge Problems. Preprint at arXiv 2023;https://doi.org/10.48550/arXiv.2303.13375.

S3 Black, S., Biderman, S., Hallahan, E., Anthony, Q., Gao, L., Golding, L., et al. Gpt-neox-20b: An open-source autoregressive language model. Preprint at arXiv 2022;https://doi.org/10.48550/arXiv.2204.06745.

S4 Team, M.N.. Introducing mpt-30b: Raising the bar for open-source foundation models. www.mosaicml.com/blog/mpt-30b; 2023. Accessed: 2023-06-22.

S5 Almazrouei, E., Alobeidli, H., Alshamsi, A., Cappelli, A., Cojocaru, R., Debbah, M., et al. Falcon-40B: an open large language model with state-of-the-art performance. https://falconllm.tii.ae; 2023.

S6 Dettmers, T., Pagnoni, A., Holtzman, A., Zettlemoyer, L.. QLoRA: Efficient Finetuning of Quantized LLMs. Preprint at arXiv 2023;https://doi.org/10.48550/arXiv.2305.14314.

S7 Zheng, L., Chiang, W., Sheng, Y., Zhuang, S., Wu, Z., Zhuang, Y., et al. Judging LLM-as-a-judge with MT-bench and Chatbot Arena. Preprint at arXiv 2023;https://doi.org/10.48550/arXiv.2306.05685.

S8 Touvron, H., Martin, L., Stone, K., Albert, P., Almahairi, A., Babaei, Y., et al. Llama 2: Open Foundation and Fine-tuned Chat Models. Preprint at arXiv 2023;https://doi.org/10.48550/arXiv.2307.09288.

S9 Chung, H.W., Hou, L., Longpre, S., Zoph, B., Tay, Y., Fedus, W., et al. Scaling Instruction-finetuned Language Models. Preprint at arXiv 2022;https://doi.org/10.48550/arXiv.2210.11416.

S10 Singhal, K., Tu, T., Gottweis, J., Sayres, R., Wulczyn, E., Hou, L., et al. Towards Expert-level Medical Question Answering with Large Language Models. Preprint at arXiv 2023;https://doi.org/10.48550/arXiv.2305.09617.

S11 Gu, Y., Tinn, R., Cheng, H., Lucas, M., Usuyama, N., Liu, X., et al. Domain-specific language model pretraining for biomedical natural language processing. ACM Transactions on Computing for Healthcare (HEALTH) 2021;3(1):1–23.

S12 Pal, A., Umapathi, L.K., Sankarasubbu, M.. Medmcqa: A large-scale multi-subject multi-choice dataset for medical domain question answering. PMLR 2022;174:248–260.

S13 Yasunaga, M., Leskovec, J., Liang, P.. LinkBERT: Pretraining Language Models with Document Links. Preprint at arXiv 2022;https://doi.org/10.48550/arXiv.2203.15827.

S14 Luo, R., Sun, L., Xia, Y., Qin, T., Zhang, S., Poon, H., et al. BioGPT: Generative Pre-trained Transformer for Biomedical Text Generation and Mining. Preprint at arXiv 2022;https://doi.org/10.48550/arXiv.2210.10341.

S15 Venigalla, A., Frankle, J., Carbin, M.. PubMed GPT: A domain-specific large language model for biomedical text. https://hai.stanford.edu/news/stanford-crfm-introduces-pubmedgpt-27b; 2022. Accessed: 2022-12-16.

S16 Taylor, R., Kardas, M., Cucurull, G., Scialom, T., Hartshorn, A., Saravia, E., et al. Galactica: A Large Language Model for Science. Preprint at arXiv 2022;https://doi.org/10.48550/arXiv.2211.09085.

S17 Jin, Q., Dhingra, B., Liu, Z., Cohen, W.W., Lu, X.. PubMedQA: A Dataset for Biomedical Research Question Answering. Preprint at arXiv 2019;https://doi.org/10.48550/arXiv.1909.06146.

S18 Hendrycks, D., Burns, C., Basart, S., Zou, A., Mazeika, M., Song, D., et al. Measuring Massive Multitask Language Understanding. Preprint at arXiv 2020;https://doi.org/10.48550/arXiv.2009.03300.

S19 Wang, X., Wei, J., Schuurmans, D., Le, Q.V., Chi, E.H., Zhou, D.. Self-consistency Improves Chain of Thought Reasoning in Language Models. Preprint at arXiv 2022;https://doi.org/10.48550/arXiv.2203.11171.

S20 Jin, D., Pan, E., Oufattole, N., Weng, W.H., Fang, H., Szolovits, P.. What disease does this patient have? a large-scale open domain question answering dataset from medical exams. Applied Sciences 2021;11(14):6421.
